# Cardiomyocyte S1PR1 promotes cardiac regeneration via AKT/mTORC1 signaling pathway

**DOI:** 10.7150/thno.103797

**Published:** 2025-01-02

**Authors:** Xiuxiang Liu, Jinnan Yue, Caixia Zhou, Yunhao Duan, Xiaoli Chen, Jie Liu, Shougang Zhuang, Yu Luo, Jinjin Wu, Yuzhen Zhang, Lin Zhang

**Affiliations:** 1 State Key Laboratory of Cardiovascular Diseases and Medical Innovation Center, Shanghai East Hospital, School of Medicine, Tongji University, Shanghai, 200120, China.; 2 Department of Medicine, Rhode Island Hospital and Warren Alpert Medical School of Brown University, Providence, Rhode Island, USA.; 3 Department of Nephrology, Shanghai East Hospital, Tongji University School of Medicine, Shanghai, 200120, China.; 4 Department of Cardiology, Zigong Fourth People's Hospital, Sichuan, 643099, China.; 5 Department of Cardiology, Shanghai Children's Medical Center, Shanghai Jiao Tong University School of Medicine, Shanghai, China.; 6 Clinical Center for Heart Disease Research, School of Medicine, Tongji University, Shanghai, China.

**Keywords:** sphingosine 1-phosphate receptor 1, mTORC1, cardiomyocytes, myocardial infarction, cardiac regeneration

## Abstract

**Aims:** Lower vertebrates and some neonatal mammals are known to possess the ability to regenerate cardiomyocyte and fully recover after heart injuries within a limited period. Understanding the molecular mechanisms of heart regeneration and exploring new ways to enhance cardiac regeneration hold significant promise for therapeutic intervention of heart failure. Sphingosine 1-phospahte receptor 1 (S1PR1) is highly expressed in cardiomyocytes and plays a crucial role in heart development and pathological cardiac remodeling. However, the effect of cardiomyocyte-expressing S1PR1 on heart regeneration has not yet been elucidated. This study aims to investigate the role of cardiomyocyte S1PR1 in cardiac regeneration following heart injuries.

**Methods and Results:** We generated cardiomyocyte (CM)-specific *S1pr1* knock-out mice and demonstrated that CM-specific *S1pr1* loss-of-function severely reduced cardiomyocyte proliferation and inhibited heart regeneration following apex resection in neonatal mice. Conversely, AAV9-mediated CM-specific* S1pr1* gain-of-function significantly enhanced cardiac regeneration. We identified that S1PR1 activated the AKT/mTORC1/CYCLIN D1 and BCL2 signaling pathways to promote cardiomyocyte proliferation and inhibit apoptosis. Moreover, CM-targeted gene delivery system via AAV9 to overexpress S1PR1 significantly increased cardiomyocyte proliferation and improved cardiac functions following myocardial infarction in adult mice, suggesting a potential method to enhance cardiac regeneration and improve cardiac function in the injured heart.

**Conclusions:** This study demonstrates that CM-S1PR1 plays an essential role in cardiomyocyte proliferation and heart regeneration. This research provides a potential strategy by CM-targeted S1PR1 overexpression as a new therapeutic intervention for heart failure.

## Introduction

Heart failure (HF) is a heterogenous and life-threatening syndrome characterized by ventricular dysfunctions. The predominant cause of heart failure is coronary heart diseases (CHD) 1, 2. Despite significant advancements in CHD management, which have markedly reduced the acute CHD-related mortality in recent decades, the incidence of heart failure continues to rise 2. Current clinical treatments for heart failure patients enhance cardiac functions and improve quality of life, yet these therapeutic interventions do not adequately compensate for the loss of functional cardiomyocytes 3. In case of end-stage heart failure, heart transplantation remains the gold standard treatment. However, the scarcity of suitable donors and the risk of immune rejection severely limit the availability of this option for eligible patients 4. Consequently, there is an urgent clinical need for a fundamental therapeutic approach that promotes cardiomyocyte regeneration and restores cardiac functions.

Adult mammalian cardiomyocytes (CMs) exhibit limited proliferative capacity, resulting in a permanent loss of functional cardiomyocytes following heart injuries, which contributes to the development of heart failure and cardiac dysfunctions 5, 6. In contrast, lower vertebrates, including zebrafish and xenograft, exhibit remarkable heart regenerative capacity after cardiac injuries 5, 6. The neonatal mammalian heart has significant regenerative capability following heart injuries within a short period after birth 6. In these instances, pre-existing CMs undergo dedifferentiation and re-enter the cell cycle, leading to cardiomyocyte proliferations and the formation of new CMs 6. Recent studies have demonstrated that newborn mice exhibit robust regenerative capabilities in injured myocardium within the first week after birth 6, 7. A noteworthy clinical case of a newborn who exhibited complete recovery of cardiac function following a severe myocardial infarction suggests that human myocardium may possess a similar regenerative potential as seen in other mammals 8. However, this regenerative capability diminishes progressively with age and becomes severely limited in adult hearts 6. Understanding the key molecular mechanisms regulating cardiac regeneration is crucial for developing innovative therapies for treating heart failure.

Sphingosine 1-phosphate (S1P), a biologically active lipid molecule, is a critical regulator in the development and functions of the cardiovascular system 9. S1P exerts its effects through a family of five G protein-coupled receptors (S1PR1-S1PR5), imparting S1P with diverse and multifaceted biological functions 10. During embryogenesis, S1P signaling is crucial for vascular maturation and the stabilization of nascent blood vessels 11. Studies have demonstrated that S1P, via S1PR1, promotes endothelial cell migration, proliferation, and the formation of adherens junctions, all of which are essential for the integrity and functionality of the vascular network 11, 12. Mice lacking S1PR1 exhibit defective vascular maturation, leading to embryonic lethality, indicating the indispensable role of S1PR1 in vascular development 11. It has been shown that S1P signaling regulated cardiac precursor cell (CPC) migration during zebrafish heart tube formation and that defects in S1P signaling disrupt endodermal integrity, leading to endodermal apoptosis and cardia bifida 13, 14, highlighting its importance in cardiac tissue development.

Our previous studies have identified the expression of three S1P receptors, including S1PR1, S1PR2 and S1PR3, in cardiac tissues 15. Notably, S1PR1 is predominantly expressed in cardiomyocytes, and prior studies have shown that cardiomyocyte-expressing S1PR1 influences heart development and the pathological processes of heart diseases 16-18. However, the role of CM-S1PR1 in the regulation of cardiac regeneration following heart injuries remains unclear.

Our findings demonstrated that cardiomyocyte (CM)-specific *S1pr1* loss-of-function impaired cardiomyocyte proliferation and inhibited heart regeneration. In contrast, *S1pr1* overexpression in CMs significantly enhanced cardiac regeneration and improved cardiac functions following heart injuries in neonatal mice. Mechanistically, S1P-S1PR1 signaling promoted cardiac regeneration and reduced cardiomyocyte apoptosis via the AKT/mTORC1/CYCLIN D1 and BCL2 pathway. Moreover, CM-targeted gene delivery of *S1pr1* to achieve CM-specific S1PR1 overexpression significantly boosted cardiomyocyte proliferation and improved cardiac functions after myocardial infarction in adult mice. This study suggests a promising CM-targeted therapy for myocardial infarction and heart failure by promoting cardiac regeneration through the S1PR1 signaling pathway.

## Methods

### Generation of cardiomyocyte-specific *S1pr1* knockout (*S1pr1^CMKO^*) mice

The conditional *S1pr1* knock-out (*S1pr1^flox/flox^*) mice were gained from Jax Mice (Stock number 019141). Cardiomyocyte-specific *S1pr1* loss-of-function mice were generated by crossing the conditional *S1pr1* loss-of-function (*S1pr1^flox/flox^*) mice with the M*yh6-Cre^ERT2^* (Cre^+/-^) mice 19, 20. All animals were housed under pathogen-free conditions with sufficient standard mouse chow and water. Before the experiment, animals were anaesthetized by intraperitoneal injection of pentobarbital sodium (50 mg/kg, *i.p*.). Mice were sacrificed by an overdose of anesthesia with pentobarbital sodium (150 mg/kg, *i.p.*) at the indicated time point, or euthanized by CO_2_ inhalation followed by cervical dislocation at the end of the experiments. All experiments were performed in accordance with the guidelines from the NIH Guide for the Care and Use of Laboratory Animals and approved by the University Committee on Animal Care of Tongji University with license number TJBB00420101.

### Tamoxifen administration

Tamoxifen (#T5648, Sigma) was dissolved in 90% peanut oil/10% alcohol, and administered at the first two days after birth (40 µg per day) in neonatal mice. 100 mg/kg tamoxifen was administered via *i.p.* injection every other day for a total of 2 injections in adult mice.

### Neonatal mouse apical resection model

The heart apical resection (AR) surgery of neonatal mouse was performed as described previously 21. It is well-established that the regenerative capacity of neonatal hearts diminishes rapidly 7 days after birth. P1 mice exhibit a robust regenerative response, and P3 mice display a partial regenerative potential 22, 23. For our study, apical resection was performed in P3 mice to accommodate the need for prior pharmacological treatment (tamoxifen) or virus injection (AAV9). Briefly, neonatal mouse pups of 3-day-old (P3) were anesthetized by placement on ice for 5 minutes to ensure that the pups are sufficiently anesthetized before proceeding with the following operation. We used fine forceps and surgical scissors to make a small incision in the mouse thoracic cavity to expose the apex of the mouse heart. The skin and muscle layers were retracted to avoid unnecessary tissue damage. The apex of heart was exposed and resected by iridectomy scissors to ensure that the resection was precise to minimize tissue damage and bleeding, and the thoracic cavity wall and the skin were then closed with an 8-0 suture. The pups were subsequently placed on a heating pad to gently warm them until they fully recovered from anesthesia. Once the pups have regained normal movements and responses, returned them to their home cages. The hearts of mice were collected at 7 days post-AR for analysis of cardiomyocyte proliferation and apoptosis because this time point is critical for detecting early cellular responses and proliferative activity, as established by previous studies 22-24. At 21 days post-AR, the hearts of mice were collected to assess longer-term tissue remodeling and scar formation, as performed in prior investigations 22-24.

### Adult mouse myocardial infarction model

Eight-week-old adult gender-matched mice were anesthetized with pentobarbital sodium (50 mg/kg, *i.p.*), and mechanically ventilated (isoflurane 1-2% vol/vol) with a rodent respirator device (ALC-V8S, ALCBIO). To ensure the appropriate depth of anesthesia, the absence of reflexes, including the pedal withdrawal reflex, was verified. Subsequently, the anesthetized mice were positioned supine and securely fixed to the surgical board for the procedure. A small incision was made in the left thoracic region using fine forceps and a surgical blade to gain access to the heart. Skin and muscle layers were retracted carefully to expose the thoracic cavity and the heart. Retractors were utilized to maintain the incision site open if necessary. Subsequently, the left anterior descending coronary artery (LAD) of the mouse was identified and permanently ligated with an 8-0 suture to induce myocardial infarction by restricting blood flow to the downstream myocardium. Retractors were carefully removed, and the thoracic cavity was then closed using a 6-0 suture with either interrupted or continuous sutures. The skin incision was closed with a 4-0 suture. The mice were subsequently placed on a heating pad to maintain body temperature during their recovery from anesthesia and then put them back to home cages. After recovery, repeat analgesic administration was given with carprofen (10 mg/kg) every 24 hours and buprenorphine (0.1 mg/kg) every 12 hours for 48 hours. The hearts of 8-week-old mice in the indicated groups were collected at 7 days post-MI for detecting early cellular responses and proliferative activity, as reported in previous studies 24, 25. At 28 days post-MI in 8-week-old mice in the indicated groups, morphological analyses, including quantification of scar tissue were conducted to assess longer-term tissue remodeling and scar formation, in accordance with prior research 24, 25.

### Construction and injection of AAV vectors in neonatal and adult mice *in vivo*

S1PR1 overexpressing adeno-associated virus 9 (AAV9) driven by cardiac-specific promoter *cTnT* (AAV9-*cTnT*-*S1pr1*-*GFP*) and control viral vectors (AAV9-*cTnT*-*GFP*) were constructed by Hanbio Biotechnology Co., Ltd. For the neonatal mouse experiments, the neonatal pups of 1-day-old (P1) were intraperitoneally (*i.p.*) injected with AAV9-*cTnT*-*S1pr1*-*GFP* or AAV9-*cTnT*-*GFP* at a dose of 8 × 10^9^ viral genome particles per mouse following AR operation at postnatal day 3, and their hearts were collected at 7 days post-AR for analysis of cardiomyocyte proliferation and apoptosis, and at 21 days post-AR for analysis of cardiac scars and cardiac function. For the adult mouse experiments* in vivo*, 8-week-old adult mice were intravenously (*i.v.*) injected with AAV9-*cTnT*-*S1pr1*-*GFP* or AAV9-*cTnT*-*GFP* at a dose of 4 × 10^11^ viral genome particles per mouse. The adult mice were infected with AAV9-*cTNT*-*S1pr1*-*GFP* or AAV9-*cTNT*-*GFP* following MI operation, and their hearts were collected at 7 days post-MI for analysis of cardiomyocyte proliferation and apoptosis, and at 28 days post-MI for analysis of cardiac scars and cardiac function. In pharmacological experiments, rapamycin, an mTORC1 antagonist (2 mg/kg, #S1842, Beyotime), and DMSO were administrated via intraperitoneal injection every day after AR or MI operations.

### Echocardiography analysis

As we previously described 26, the visual sonics high-resolution Vevo2100^TM^ ultrasound system (Visual Sonics Inc., Canada) with a 30-MHz linear array ultrasound transducer (MS-400, Visual Sonics Inc.) was used to perform echocardiography analysis of experimental animals. Briefly, mice were anesthetized under conditions of 1% isoflurane with the stable heart rate using an induction chamber. Once the mouse was anesthetized, anesthesia was maintained using a nose cone or mask. The anesthetized mouse was then placed in the supine position on a heating pad to prevent hypothermia. A small amount of ultrasound gel was applied to the mouse chest area to ensure a good contact between the transducer and the skin. Parasternal long-axis (PSLAX) and short-axis (PSSAX) views were initially acquired. The B-mode imaging was used to assess overall cardiac morphology and wall motion. M-mode imaging was employed to measure ventricular dimensions, wall thickness, left ventricular end-diastolic diameter (LVEDD) and left ventricular end-systolic diameter (LVESD). Doppler imaging was utilized to evaluate blood flow velocities across the mitral valve and aortic outflow. Additionally, tissue Doppler imaging (TDI) was optionally performed to assess myocardial velocities. Left ventricular ejection fraction (LVEF%) and fractional shortening (LVFS%) were calculated based on the end-diastolic and end-systolic dimensions.

### ECG measurement

We assessed heart rhythm by the electrocardiogram. Briefly, mice were anesthetized with 1% isoflurane. The anesthetized mouse was placed in a supine position on the heating pad to prevent hypothermia. Following the successful anesthesia of the animal, an electrode needle was inserted subcutaneously into each limb of the animal, according to the prescribed connection method: red for the right upper limb, yellow for the left upper limb, green for the left lower limb, and black for the right lower limb. Labchart Installers System Requirements (Version 8.1.10, Labchart) was stared to acquire baseline ECG traces. The ECG recording software was used to analyze key parameters, including heart rate, PR interval, QRS duration, QT interval, and ST-segment changes.

### RNA microarray

Sham-operated and apical resection (AR) models were established in 3-day-old neonatal mouse of both *WT* and *S1pr1^CMKO^*. Hearts were harvested 1 day after AR operation. The total RNA from hearts was extracted using Trizol reagent (Invitrogen). The quality and quantity of the total RNA were measured by a Nanodrop 2000. Microarray analysis experiments were performed by Genminix Informatics (Shanghai) using AffymetrixMTA1.0, which provided full coverage of mouse coding and non-coding transcripts. The original microarray data discussed in this study were submitted on a public database and can be accessed via the Gene Expression Omnibus series accession number: GSE241722.

### Histology

Mouse hearts were harvested and fixed with 4% paraformaldehyde at 4°C overnight. Serial sections of paraffin-embedded tissue, cut at 6 μm intervals, were stained with Masson's trichrome staining (Masson) (#60532ES74, Yeasen) for the measurement of cardiac scars. Wheat germ agglutinin (WGA) (#25500, AAT Bioquest) staining was applied for analysis of cardiomyocyte size. Biotinylated-isolectin B4 (IB4) (FL-1201, Vector Laboratories) staining was used for the measurement of cardiac capillary density. Immunofluorescence staining experiment was accomplished using primary antibodies (List see **Table S1**) and the corresponding secondary antibodies, including Alexa Fluor 488-conjugated donkey anti-mouse secondary antibodies (#Ab150113, Abcam), Alexa Fluor 488-conjugated donkey anti-rabbit secondary antibodies (#Ab150077, Abcam), Alexa Fluor 594-conjugated donkey anti-mouse secondary antibodies (#Ab150108, Abcam) and Alexa Fluor 594-conjugated donkey anti-rabbit secondary antibodies (#Ab150116, Abcam). Slides were mounted with mounting medium containing DAPI (#D9542, Sigma Aldrich). Images were collected using a Leica microscope (DM6000B, Leica, Germany) and a confocal microscope (STED 3X, Leica, Germany).

### Analysis of cardiac scars

In the neonatal apical resection model, the average percent of the scar area was calculated from the three largest ventricular longitudinal sections stained with Masson's trichrome, using ImageJ software. For the adult myocardial infarction model, five serial Masson-stained sections from the apex to the ligation site were analyzed to determine the average percent fibrotic area of the total left ventricle using ImageJ software.

### Analysis of Cardiomyocyte apoptosis by TUNEL staining

The TUNEL immunofluorescence staining was applied to evaluate and analyze the level of cell apoptosis *in vivo* and* in vitro*. *In vivo*, mouse hearts were harvested at 7 days after heart injuries and fixed with 4% paraformaldehyde at 4°C overnight. After dehydration and optimal cutting temperature compound (OCT) embedding, the hearts were sectioned into 6 μm -thick frozen slices, followed by removing OCT with PBS and rehydration. *In vitro,* primary neonatal mouse cardiomyocytes (NMCMs) were isolated and seeded in small round glass slides at a density of 2 × 10^5^ cells per well. After starvation for 12 hours, NMCMs or siRNA-transfected NMCMs were treated with or without, SEW2871 (200 nM) or LY294002 (25 μM) under the normoxic or hypoxic (95% N_2_/5% CO_2_) conditions for 24h 27-29, and then these cells were subjected to reoxygenation in a standard incubator (5% CO_2_/95% air) in normal medium for 12 hours. Then the cells fixed with 4% paraformaldehyde (PFA) for 15 minutes. The staining method for cardiac cryosections and primary NMCMs were performed using an In Situ Cell Death Detection kit (#12156792910, Roche) according to the manufacture's protocols. Briefly, cryosections and cells were permeabilized with 0.25% TritonX-100 for 1 hour, and blocked with 3% goat serum for 1 hour. After overnight incubation with α-sacromeric actinin (αSA) (#Ab9465, Abcam) at 4ºC, samples were incubated with corresponding secondary antibody and then stained with In Situ Cell Death Detection kit (#12156792910, Roche) as well as the nuclei stained with DAPI. At least 5 fields were photographed with Leica fluorescent microscope (Leica DMi8, Germany) and analyzed using ImageJ software for each individual sample. The ratio of cardiomyocyte apoptosis was represented by calculating the percentage of TUNEL-positive cardiomyocyte number to total αSA positive cardiomyocyte number.

### EdU incorporation assay* in vivo*

EdU (5-ethynyl-2'-deoxyuridine) was injected intraperitoneally (*i.p.*) at a dose of 50 mg/kg body weight into each animal. In neonatal mice, EdU was administered at 6.5-day post-AR in neonatal mice. In adult mice, EdU was injected daily for 3 consecutive days beginning from 4-day post-MI. For EdU staining, the heart sections were incubated with the reagents from the BeyoClick™ EdU-555 Kit (#C0075L, Beyotime) according to the manufacturer's instructions. Meanwhile, cardiomyocytes were stained with anti-αSA antibody (#Ab9465, Abcam) as well as the nuclei stained with DAPI. The number of α-SA positive and EdU positive cells was counted to calculate the percentage of EdU positive cells among α-SA positive cells for the assessment of cell proliferation rates.

### Isolation of mouse cardiac endothelial cells

Following the apical resection (AR) or sham surgical procedures on neonatal mice, hearts were harvested with great care to preserve structural integrity for subsequent cellular isolation. The extracted hearts were dissected and dissociated using a combination of trypsinization and collagenase (#LS004196, Worthington). The resulting cell suspension was filtered through a 70-μm nylon strainer to remove large debris and aggregates. Subsequently, single cells were labeled with an anti-mouse CD31 antibody (#553370, BD Pharmingen), which was conjugated to DynabeadsTM (#11035, Invitrogen), to isolate endothelial cells (ECs) according to the manufacturer's protocol. The ECs were subsequently collected for further experiments. The purity of isolated ECs was 94.62% ± 0.82% (mean ± SD), as confirmed by cytometric analysis using APC-CD31 antibody staining (#561814, BD Pharmingen) (**Figure S1C**).

### Isolation of mouse cardiac fibroblasts

Neonatal mice were euthanized humanely, adhering strictly to ethical guidelines, prior to harvesting their hearts. The hearts were carefully excised and dissociated using collagenase (#LS004196, Worthington). The cell suspension was passed through a 70-μm nylon strainer. The resulting single-cell suspension was labeled with an anti-mouse CD31 antibody (#553370, BD Pharmingen) conjugated to Dynabeads^TM^ (#11035, Invitrogen) for endothelial cell separation. The remaining cells were incubated with FITC-CD31 antibody (#561813, BD Pharmingen), PE-mouse lineage (CD3ε/GR1/CD11b/CD45) cocktails (#78035, Biolegend), and APC-PDGFα antibody (#135907, Biolegend). Lineage^-^CD31^-^PDGFα^+^ cardiac fibroblasts were enriched by flow cytometric sorting. The representative flow cytometric plots for a typical lineage^-^CD31^-^PDGFα^+^ cell sorting procedure are shown in **Figure S1A**, and the purity of isolated cardiac fibroblasts cells was 99% ± 0.89% (mean ± SD) (**Figure S1B**).

### Neonatal cardiomyocytes isolation and cultivation

Neonatal mouse cardiomyocytes (NMCMs) were isolated at ages P1-P3 using Neonatal Heart Dissociation Kit (Miltenyi Biotec, #130-098-373) according to the manufacturer's instructions, as reported previously 21. Briefly, neonatal rat or mouse hearts were harvested and transferred into a 10-cm dish containing DMEM supplemented with 10% FBS and 1% penicillin and 1% streptomycin. Residual blood was carefully squeezed out of the hearts using forceps, followed by the removal of vessels and connective tissue from the ventricles. The hearts were minced into tiny pieces with forceps, mixed with enzyme solution from the kit, and transferred to a gentle-MACS C Tube. The gentle-MACS Dissociator was used twice, followed by 15-min incubation at 37°C. To halt enzyme activity, 7.5 ml of cell culture medium with 10% FBS was added to the cell suspension. The single cell suspensions were then passed through a 70-μm cell strainer, centrifuged at 500 g for 10 minutes, and resuspended in DMEM supplemented with 10% FBS and 5-bromo-20-deoxyuridine (100 μM). Cells were seeded onto 6-cm plastic dishes for 2 hours at 37°C to remove fibroblasts, and subsequently plated on 1% gelatin-coated plastic culture dishes at an appropriate cell density. After 24 hours, the medium was changed to 2% serum medium, and the cells were cultured for an additional 48 hours before use. The purity of isolated neonatal cardiomyocytes cells was 95.71% ± 1.14% (mean ± SD), as confirmed by immunostaining for the cardiomyocyte marker α-SA (Abcam, #Ab9465) (**Figure S1D**).

### Isolation of cardiomyocytes from adult mice

Cardiomyocytes were isolated from adult mice as previously established protocol 30. Briefly, adult mice were anesthetized with 2% isoflurane and their chests were opened to fully expose the heart. The inferior vena was removed, and the heart was immediately perfused with 7 ml of Tyrode's buffer (NaCl, 130 mmol/L; KCl, 5 mmol/L; HEPES, 10 mmol/L; BDM, 10 mmol/L; NaH_2_PO_4_, 0.5 mmol/L; Taurine, 10 mmol/L; EDTA, 5 mmol/L; Glucose, 10 mmol/L; pH adjusted to 7.8 with NaOH) via the right ventricle. Reynold's forceps were used to clamp the ascending aorta, and the whole heart was placed in a fresh 10-cm dish containing Tyrode's buffer. The hearts were then mounted on a Langendorff perfusion system and digestion was achieved by sequential injection of 10 ml Tyrode's buffer, 3 ml perfusion buffer (NaCl, 130 mmol/L; KCl, 5 mmol/L; HEPES, 10 mmol/L; BDM, 10 mmol/L; NaH_2_PO_4_, 0.5 mmol/L; Taurine, 10 mmol/L; MgCl_2_, 1 mmol/L; Glucose, 10 mmol/L; pH adjusted to 7.8 with NaOH), and 30 ml collagenase buffer (Collagenase II, 0.5 mg/ml; Collagenase IV, 0.5 mg/ml; Protease XIV, 0.05 mg/ml; Dilution in Perfusion buffer and make fresh immediately before isolation) into the left ventricle (LV). The collagenase infusion was stopped by 5 ml stop buffer (EDTA buffer containing 5% sterile FBS) once the heart appeared adequately digested, and the clamp was removed. Heart tissues were subsequently minced into 1 mm^3^ sections using forceps and gently dissociated by pipetting. Cellular dissociation was further facilitated by gentle digestion. Then the cell suspension was filtered through a 100-μm cell strainer (BD Biosciences, Franklin Lakes, NJ, USA). Cardiomyocytes were collected by gravity settling in 3 sequential rounds (20 minutes/round). The cell pellet from each round was enriched with cardiomyocytes, ultimately yielding a highly pure cardiomyocyte fraction. Finally, this cell pellet was washed three times with PBS and then lysed using Trizol or RIPA lysis buffer for subsequent analysis of mRNA or protein expression levels.

### siRNA transfection in NMCMs

Small interfering RNAs (siRNAs) targeting *S1pr1*, *Bcl2*, *Raptor*, and *Tsc1* (GenePharma) were used to knockdown these gene expression. The specific siRNA sequences are listed in **Table S2.** The siRNA transfection cardiomyocytes were performed using Lipofectamine 3000 (Invitrogen, Waltham, MA, USA) according to the manufacturer's detailed instructions. Briefly, cardiomyocytes were cultured in appropriate dishes and grown to 70-80% confluence. The siRNA-Lipofectamine 3000 complexes were prepared by diluting the siRNAs (200 pmol) and Lipofectamine 3000 reagent (10 μl) in 1 ml Opti-MEM medium for 60-mm dish and incubating the mixture for 20 minutes at room temperature to facilitate complex formation. These complexes were then added to the NMCMs and incubated under standard culture conditions (37°C, 5% CO_2_). After 6-8 hours, the transfection medium was replaced with fresh complete medium or serum-free medium for subsequent experiments. Cells were harvested or treated with other reagents at 48 hours post-transfection. The efficiency of gene knockdown was assessed using western blot and quantitative real-time PCR (RT-qPCR).

### Immunofluorescence assay

For NMCMs cultured on the glass slides, cells were fixed with 4% paraformaldehyde for 15 minutes, permeabilized with PBS containing 0.25% Triton X-100 at room temperature for 1 hour, and then blocked with 3% BSA for 1 hour at room temperature. After overnight incubation with primary antibodies at 4ºC, cells were incubated with appropriate fluorescence-labeled secondary antibody. The following primary antibodies were used (**Table S1**): α-sacromeric actinin (αSA) (Abcam, #Ab9465, 1:200), Ki67 (Abcam, Ab15580, 1:200), phospho-histone H3 (PH3) (Abcam, #Ab80612, 1:200) and S1PR1 (Invitrogen, #PA1-1040, 1:100). The secondary antibodies conjugated to goat anti-mouse Alexa Fluor 488 (Abcam, #Ab150113, 1:1000), and goat anti-mouse Alexa Fluor 594 (Abcam, #Ab150116, 1:1000) were used. Cells were mounted with medium containing DAPI in dark. At least 5 fields were photographed with Leica fluorescent microscope (Leica DMi8, Germany) and analyzed using ImageJ software for each individual sample. The ratio of cardiomyocyte proliferation was represented by calculating the percentage of Ki67 or PH3 positive cardiomyocyte number to total αSA -positive cardiomyocyte number.

### Flow cytometric analysis for cardiomyocyte apoptosis* in vitro*

Primary neonatal mouse cardiomyocytes (NMCMs) were isolated and seeded at a density of 2 × 10^5^ cells per well. After starvation for 12h, NMCMs or siRNA-transfected NMCMs were treated with or without SEW2871 (200 nM) or LY294002 (25 μM) under the normoxic or hypoxic (95% N_2_/5% CO_2_) conditions for 24h 27-29, and then these cells were subjected to reoxygenation in a standard incubator (5% CO_2_/95% air) in normal medium for 12 hours. Following treatment, NMCMs were harvested and washed 3 times with pre-cooled PBS. Cells were then stained propidium iodide (PI) and FITC Annexin V according to the manufacturer's instructions using FITC Annexin V Apoptosis Detection Kit (#C1062M, Beyotime). Data were recorded with an LSR II flow cytometer and analyzed with FlowJo (Version 10.0).

### RNA purification and RT-qPCR

Total RNA from heart tissues, isolated neonatal and adult cardiomyocytes was extracted using Trizol. We treated RNA with DNase I for 30 minutes at 37 °C to remove residual DNA contamination. RNA quantity and quality were determined using Nanodrop ND-2000 spectrophotometer (Thermo Scientific, USA). The cDNA was synthesized using the SuperScript First Strand Synthesis System according to the manufacturer's instructions (#R223-01, Vazyme Biotech). RT-qPCR was then performed using PowerUp SYBR Green master mix (#Q511-02, Vazyme Biotech) on a QuantStudio 6 Flex Real-time PCR system. Transcript levels were normalized to *Gapdh* expression and analyzed using the 2^-DeltaDeltaCT^ method to determine relative gene expression. This method involves calculating the CT values for both the gene of interest and the housekeeping gene in treated and untreated samples. The ΔCT value is obtained by subtracting the CT value of the housekeeping gene from the CT value of the gene of interest for each sample. The ΔΔCT value is then calculated by subtracting the ΔCT of the untreated sample from the ΔCT of the treated sample, allowing for the determination of relative fold gene expression levels. Primer sequences are listed in **Table S3**.

### Western blot analysis

Tissue homogenates or cell pellets were lysed using RIPA buffer (#P0013C, Beyotime). The lysate was centrifuged at 2,500 g for 10 minutes at 4°C to collect the supernatant. Protein concentrations in the supernatant were determined using a BCA kit (#P0012S, Beyotime). Equal amounts of total protein were then separated by 10% SDS-PAGE and transferred onto polyvinylidene difluoride (PVDF) membranes. The membranes were blocked with 5% skim milk at room temperature for 1 hour to prevent non-specific binding. Following blocking, the membranes were incubated overnight at 4°C with the primary antibodies listed in **Table S1**. After three washes with Tris-buffered saline containing 0.05% Tween 20 (TBST), the membranes were incubated with the appropriate IRDye800CW-conjugated secondary antibodies listed in **Table S1** for 1 hour at room temperature. After another series of washes with TBST, the signals were visualized using the Odyssey® CLX Infrared Imaging System (LI-COR). Quantification of the bands was performed using ImageJ software, and the results were presented as bar graphs normalized to the levels of the corresponding loading controls.

### Sphingolipid measurement by mass spectrometry (LC-MS)

Fresh heart tissues were harvested one day post-AR or post-sham operation for lipidomic profiling analysis. Cellular lipids of tissues were extracted using the modified Bligh and Dyer method 31, incorporating 0.1 N HCl for phase separation. C17-S1P (1ng/mL), used as an internal standard, was added at the beginning of the extraction process. C17-S1P were prepared in a mixture of DMSO and concentrated hydrochloric acid (100:2, v/v) at a concentration of 100 µg/mL. These were diluted with methanol and stored at -20°C. For experiments, the stock solutions were further diluted with methanol to prepare working solutions. The extracted lipids were dissolved in methanol, and aliquots were taken to measure the total phospholipid content, as previously described 32. A Finnigan TSQ Quantum triple quadrupole mass spectrometer (Thermo Electron, San Jose, CA, USA) was used, with a Luna-RP C18 analytical column (150 mm length x 2 mm inner diameter, 5 µm particle size, and 100 Å pore size), equipped with a C18 guard column (4.0 mm length x 2.0 mm inner diameter) from Phenomenex (Torrance, CA, USA). The solvents and samples were separated using a Finnigan autosampler and mass spectrometer pump. The column temperature was set to 25°C. The mobile phase consisted of methanol-water (95:5, v/v), with 0.1% formic acid in the water. The flow rate was 0.2 mL/min, and the analysis time for each sample was 4 minutes. The ion source was an electrospray ionization source (ESI), with a heated capillary temperature set at 350°C. The sheath gas (N₂) pressure was 15 psi, and the auxiliary gas (N₂) pressure was 1 psi. The electrospray voltage was 3000 V. The collision gas pressure was 10 mTorr (1 Torr = 1333 Pa), and the ion source collision-induced dissociation (SCID) voltage was 10 eV. The detection mode was positive ion detection, and the scanning method was selected reaction monitoring (SRM) with a scan time of 0.1 seconds. The ion reactions used for quantifying the molecules were as follows: for S1P, m/z 380.1 → 264.2 with a collision energy of 18 V; and for C17-S1P, m/z 365.9 → 250.2 with a collision energy of 15 V.

### S1P measurement by enzyme linked immunosorbent assay (ELISA)

Heart tissues were weighed, homogenized, and sonicated in the ice-cold 20 mM lysis buffer (Tris buffer pH 7.4 containing 20% glycerol, 1 mM EDTA, 1% phosphatase inhibitor cocktail and protease inhibitor cocktail). Samples were centrifuged at 2,500 g for 10 min at 4°C to remove debris and supernatant was collected for measure of S1P concentrations using an S1P ELISA kit (K-1900, Echelon Biosciences) as according to the manufacturer's instruction. Finally, concentrations of S1P for all samples were normalized to the weight of heart tissues as relative S1P abundance.

### Mitochondrial morphology analysis

Mouse cardiomyocytes (MCMs) were seeded at a density of 2 × 10^5^ cells per well. After starvation for 12h, MCMs or siRNA-transfected MCMs were treated with or without SEW2871 (200 nM) or Rapamycin (50 nM) under the normoxic or hypoxic conditions for 24h, and then these cells were subjected to reoxygenation in a standard incubator (5% CO_2_/95% air) in normal medium for 12 hours. Cells were first fixed in 4% paraformaldehyde (PFA) solution for 15 minutes, then permeabilized with 0.1% Triton X-100 in PBS for 10 minutes. To examine mitochondrial morphology, cells were blocked in 1% goat serum in PBS for 1 hour before being incubated overnight at 4°C with the Tomm20 antibody (#ab186735, Abcam, UK). Then the secondary antibody was added for 1 hour at room temperature, followed by DAPI staining. Samples were then mounted with Fluoromount™ Aqueous Mounting Medium. Mitochondrial morphology of individual cells was visualized using a Leica SP8 confocal laser scanning microscope (Leica, USA). Mitochondrial length was analyzed by determining the average mitochondrial branch length (μm) in an unbiased manner with the Mitochondrial Network Analysis (MiNA, https://github.com/StuartLab/MiNA) ImageJ macro.

### JC-1 flow cytometric analysis

Mouse cardiomyocytes (MCMs) were seeded at a density of 2 × 10^5^ cells per well. After starvation for 12h, MCMs or *siRNA*-transfected MCMs were treated with or without SEW2871 (200 nM) or Rapamycin (50 nM) under the normoxic or hypoxic conditions for 24h, and then these cells were subjected to reoxygenation in a standard incubator (5% CO_2_/95% air) in normal medium for 12 hours. The cellular processing was conducted following the protocols and instructions provided by the manufacturer of the Enhanced Mitochondrial Membrane Potential Assay Kit with JC-1 (#C2003S, Beyotime, China). Subsequently, the fluorescence intensity of each sample was measured and quantified using a CytoFLEX flow cytometer (#A00-1- 110).

### Cytochrome C (CYCS) measurement by ELISA

Mouse cardiomyocytes (MCMs) were seeded at a density of 2 × 10^5^ cells per well. After starvation for 12h, MCMs or *siRNA*-transfected MCMs were treated with or without SEW2871 (200 nM) or Rapamycin (50 nM) under the normoxic or hypoxic conditions for 24h, and then these cells were subjected to reoxygenation in a standard incubator (5% CO_2_/95% air) in normal medium for 12 hours. Cell culture supernatant was collected after removing particulates by centrifugation and *in vitro* quantitative measurement of CYCS were conducted according to the protocols and instructions provided by the manufacturer of Mouse Cytochrome c (CYCS) ELISA Kit (RK07050, Abclonal, China).

### Statistical analysis

All continuous data were represented as means ± standard error of the mean (S.E.M.) for at least three independent assays unless otherwise noted. Student's t-test was performed for comparisons of two groups. One-way ANOVA or Two-way ANOVA were performed for multiple group comparisons. The results with P values less than 0.05 were considered statistically significant. All data were checked for normality and equal variance before using parametric tests. All analyses were performed with SPSS 11.0 (SPSS. Inc) for Windows.

## Results

### The expression of S1PR1 in neonatal hearts and its changes after cardiac injuries

**Figure 1:**
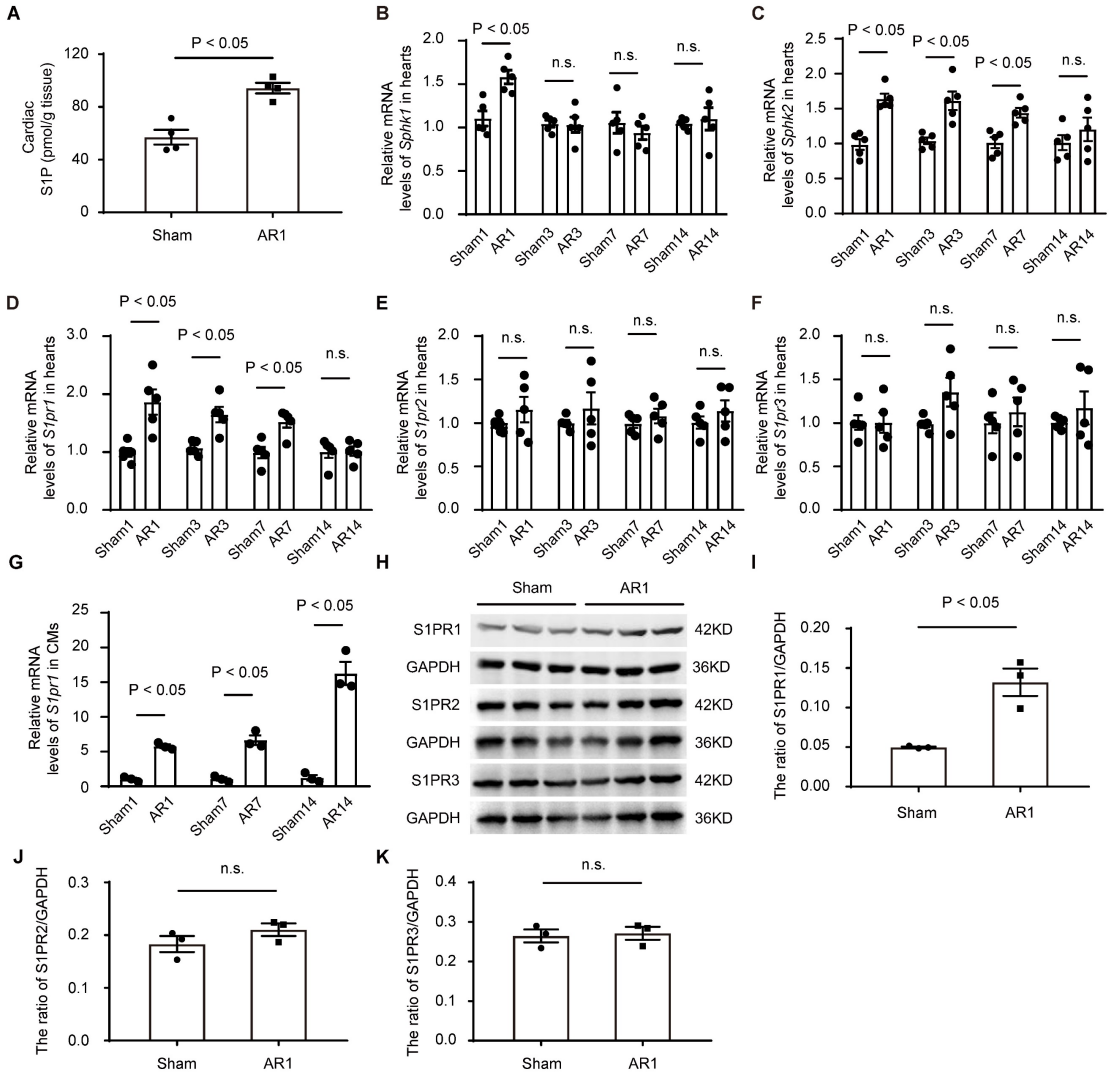
** The expression of S1PR1 in neonatal hearts and its changes after heart injury. A**. *C57BL/6J* neonatal mice underwent apical resection (AR) surgery at postnatal day 3 (P3). Cardiac sphingosine-1-phosphate (S1P) levels in heart tissues from sham-operated and 1-day post-AR mice were determined by liquid chromatography-mass spectrometry (LC-MS) (n = 4). **B**-**F**. Relative mRNA expression levels of *Sphk1* (**B**), *Sphk2* (**C**), *S1pr1* (**D**), *S1pr2* (**E**), and *S1pr3* (**F**) were determined by quantitative RT-qPCR in hearts tissues harvested from neonatal mice which underwent the sham operation or the AR operation at postnatal day 3 (P3). Samples were collected at various post-operation time points (1-day, 3-day, 7-day, and 14-day) (n = 5). **G**. Relative mRNA expression levels of *S1pr1* were determined by quantitative RT- qPCR in isolated cardiomyocytes (CMs) from neonatal mice which underwent the sham operation or the AR operation at postnatal day 3 (P3). Samples were collected at various post-operation time points (1-day, 7-day, and 14-day) (n = 3). **H**-**K**. Western blot analysis of S1PRs expression in cardiomyocytes (CMs) from the sham hearts or the AR hearts at 1 day after operation in neonatal mice which underwent sham or AR operation at postnatal day 3 (P3), as shown by the representative images of western blot (**H**) and quantifications of the ratio of S1PR1/GAPDH (**I**), S1PR2/GAPDH (**J**) and S1PR3/GAPDH (**K**) (n = 3). Sham1, 1-day post sham operation; Sham3, 3-day post sham operation; Sham7, 7-day post sham operation; Sham14, 14-day post sham operation; AR1, 1-day post AR; AR3, 3-day post AR; AR7, 7-day post AR; AR14, 14-day post AR; n.s., no significant statistical differences. Data are represented as means ± S.E.M. P < 0.05 indicates significant statistical differences. Unpaired Student's t-test (**A** and **I**-**K**), two-way ANOVA (**B-G**).

To investigate the potential role of S1P signaling in cardiac regeneration in neonatal mice, we first measured the levels of S1P in neonatal heart tissues after heart injuries using both mass spectrometry (**Figure 1A**) and ELISA (**Figure S1E**). The results indicated that S1P levels were elevated in the injured hearts induced by apex resection (AR), compared to the sham-operated control mice (**Figure 1A** and **Figure S1E**); However, S1P levels did not change at postnatal day 1, 7 and day 14 in normal neonatal mice (**Figure S1M**), suggesting that the increased S1P levels were due to neonatal heart injuries rather than postnatal heart development. Consistent with these findings and a recent study 33, we observed elevated expression of S1P synthesis enzymes, sphingosine kinase 1 (*Sphk1*) and sphingosine kinase 2 (*Sphk*2), with no changes in S1P-degrading enzymes, including sphingosine 1-phosphate phosphatase 1 (*Sgpp1*), sphingosine 1-phosphate phosphatase 2 (*Sgpp2*), and sphingosine 1-phosphate lyase (*Spl*), in neonatal hearts after AR (**Figure 1B-C**, and **Figure S1F-H**), suggesting that S1P/S1P receptor signaling might be involved in the heart regeneration process. Notably, S1P synthesis enzymes were significantly upregulated in cardiomyocytes not in non-cardiomyocytes, in AR hearts (**Figure S1I-L**).

To identify which S1P receptor subtypes might influence cardiac regeneration after heart injuries, we next measured the expression of S1P receptors in postnatal heart tissues. Our data revealed a significant increase in *S1pr1* expression in post-AR heart tissues compared to the sham group (**Figure 1D**), while no significant changes were observed in the expression of *S1pr2* and *S1pr3* between AR and sham groups (**Figure 1E-F**). Additionally, we did not detect *S1pr4* or *S1pr5* expression in neonatal heart tissues (data not shown). We further assessed the expression profile of *S1pr1* in cardiomyocytes and found that the *S1pr1* expression significantly increased in the neonatal cardiomyocytes, not in endothelial cells (ECs) nor cardiac fibroblasts (CFs), after AR compared to sham-operated controls (**Figure 1G** and **Figure S1O-P**). Consistent with these data, elevated protein levels of S1PR1 in neonatal cardiomyocytes after AR were confirmed by both western-blot and immunostaining (**Figure 1H-K** and **Figure S1N**). These findings suggest that increased S1P-S1PR1 signaling in cardiomyocytes may contribute to the regulation of cardiac regenerative capacity in neonatal mice.

### The cardiomyocyte-specific loss of S1PR1 inhibited cardiac regeneration following AR in neonatal mice

**Figure 2:**
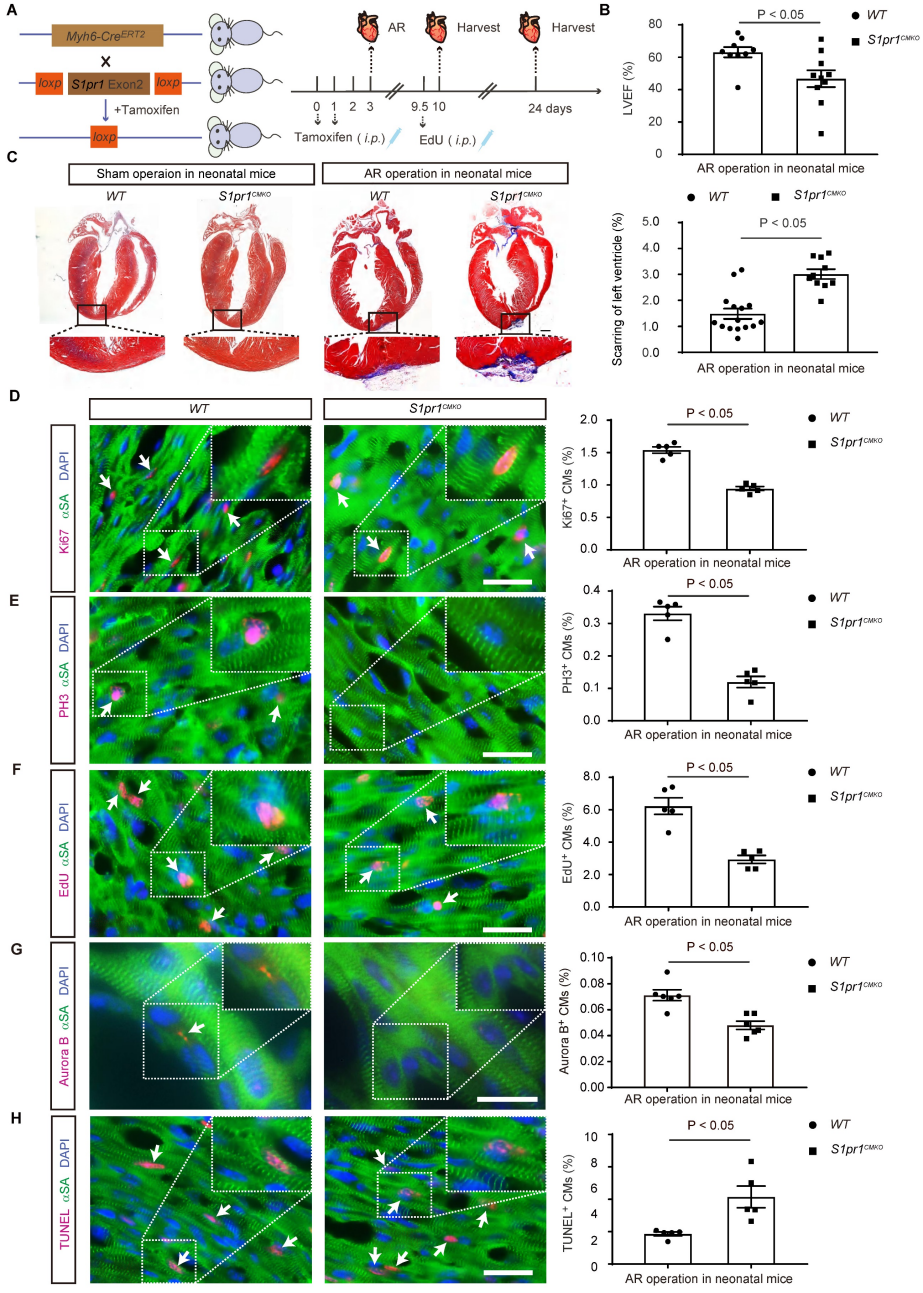
** The cardiomyocyte-specific loss of S1PR1 inhibits cardiac regeneration after AR in neonatal mice. A**. Schematic diagram of generation of cardiomyocyte-specific *S1pr1* knockout mice (*S1pr1^CMKO^*), and tamoxifen (dosed at 40 µg daily from postnatal day 0 to 1) were administered to neonatal mice to induce specific deletion of* S1pr1* in CMs following with apical resection (AR) at postnatal day 3 (P3). EdU were administered (50 mg/kg body weight,* i.p.*) at 6.5-day after AR. Hearts tissue from sham-operated and AR-operated neonatal mice were harvested at designated post-AR time points (7-day, 21-day). **B**. Quantitative assessment of left ventricle ejection fraction (LVEF%) in wild-type (*WT*) and *S1pr1^CMKO^* mice which underwent the sham operation or the AR operation at postnatal day 3 (P3) were performed at 21-day post AR using echocardiography (n = 9-10). **C**. Representative images of Masson's Trichrome staining in hearts from *WT* and *S1pr1^CMKO^* mice which underwent the sham operation or AR operation at postnatal day 3 (P3). Hearts sections were collected from these mice at 21-day post AR, and quantification of the percentage of cardiac scar area in left ventricle (n = 10-15). **D-H**. Representative immunostaining images on resection sections for Ki67 (**D**), PH3 (**E**), EdU (**F**), Aurora B (**G**) or TUNEL (**H**) and α-SA positive cardiomyocytes within the border zone of injured hearts from *WT* and *S1pr1^CMKO^* mice which underwent AR operation at postnatal day 3 (P3). Hearts sections were collected from these mice at 7-day post AR (n = 5-6). The arrows indicate α-SA (green) cardiomyocytes positive for Ki67 (magenta), PH3 (magenta), EdU (magenta), Aurora B (magenta) or TUNEL (magenta). DAPI, nuclear staining (blue). Quantification of the percentage of Ki67^+^α-SA^+^, PH3^+^α-SA^+^, EdU^+^α-SA^+^, Aurora B^+^α-SA^+^ and TUNEL^+^α-SA^+^ cardiomyocytes on the right. α-SA, α-sacromeric actinin. PH3, phospho-histone H3. EdU, 5-ethynyl-2'-deoxyuridine. Data are represented as means ± S.E.M. P < 0.05 indicates significant statistical differences. n.s., no significant statistical differences. Unpaired Student's t-test (**B**-**H**). Scale bars: **C**, 2 mm; **D**-**H**, 15 µm.

To investigate the effects of CM-expressing* S1pr1* on cardiac regeneration, we generated CM-specific *S1pr1* knock-out mice (*S1pr1^CMKO^*) by crossing* Myh6-Cre^ERT2^* mice with *S1pr1^flox/flox^* mice (**Figure 2A**). Since cardiomyocyte (CM)-specific *S1pr1* deletion impairs heart development 17, we administered tamoxifen postnatally to induce CM-specific *S1pr1* knock-out in neonatal mice without affecting embryo development. Expression levels of *S1pr1*/S1PR1 were significantly reduced in neonatal CMs of *S1pr1^CMKO^* mice after tamoxifen treatment, as confirmed by western-blotting and RT-qPCR (**Figure S2A-C**). We then examined the role of S1PR1 in heart regeneration by inducing cardiac injuries on post-natal day 3 (P3) in *S1pr1^CMKO^* and wild-type neonates via apex resection (AR) (**Figure 2A**). Although tamoxifen-induced *S1pr1* loss in CMs did not affect cardiac morphology and functions without heart injuries (**Figure 2C** and **Table S4**), CM-specific *S1pr1* deletion significantly reduced left ventricular ejection fraction (LVEF%) three weeks post-AR compared to control *WT* littermates (**Figure 2B** and **Table S4**). Our data demonstrated that *WT* neonatal mice exhibited myocardium regeneration post-AR at P3, with minimal fibrotic scarring around the injury site (**Figure 2C**), which is consistent with the previous study 34. However, CM-specific *S1pr1* deletion significantly increased the scar size with higher degree of fibrotic areas at 3 weeks after AR, in comparison with the control littermates (**Figure 2C**).

We performed histological analysis to assess the effect of CM-S1PR1 on cardiomyocyte hypertrophy. The ratio of heart weight to body weight of *S1pr1^CMKO^* mice was comparable to *WT* littermates (**Figure S2D**). Similarly, wheat germ agglutinin (WGA) immunostaining indicated that CM-specific *S1pr1* deletion induced by tamoxifen in neonates did not alter cardiomyocyte cross-sectional area following AR (**Figure S2E**), suggesting that the CM-S1PR1 deletion does not influence cardiac hypertrophy in our animal models. Additionally, isolectin-B4 (IB4) staining revealed no changes in capillary density in *S1pr1^CMKO^* neonates compared to *WT* littermates (**Figure S2F**), indicating that CM-expressing S1PR1 may not be involved in angiogenesis during cardiac regeneration.

Given that cardiomyocyte proliferation is essential for cardiac regeneration, we assessed the proliferative capacity of cardiomyocytes in *S1pr1^CMKO^* neonates. Immunostaining for proliferative markers, including Ki67 and phospho-histone H3 (PH3), showed significantly fewer Ki67-positive and PH3-positive cardiomyocytes in neonatal myocardium 7 days post-AR in CM-specific *S1pr1* deletion mice, in comparison with the *WT* littermates (**Figure 2D-E**). Pulse-chase labeling with EdU (administered at days 6.5 after AR) revealed numerous EdU-positive cardiomyocytes undergoing DNA replication in newly formed myocardium at day 7 after AR (**Figure 2F**). Our results showed that CM-specific *S1pr1* deletion mice had fewer EdU-positive cardiomyocytes than *WT* control littermates (**Figure 2F**). Furthermore, Aurora B immunostaining indicates that cardiomyocytes in *WT* neonates undergo cytokinesis at day 7 after AR, whereas significantly fewer in *S1pr1^CMKO^* neonates (**Figure 2G**). Additionally, we observed increased cardiomyocyte apoptosis in *S1pr1^CMKO^* mice hearts at 7 days post-AR, suggesting that CM-S1PR1 regulates cardiomyocyte apoptosis after heart injuries (**Figure 2H**). These findings underscore the critical role of CM-expressing S1PR1 in regulating cardiomyocyte proliferation and apoptosis during cardiac regeneration following heart injuries.

### The cardiomyocyte-specific loss of S1PR1 restrained heart regeneration and aggravated cardiac dysfunctions following myocardial infarction (MI) in adult mice

**Figure 3:**
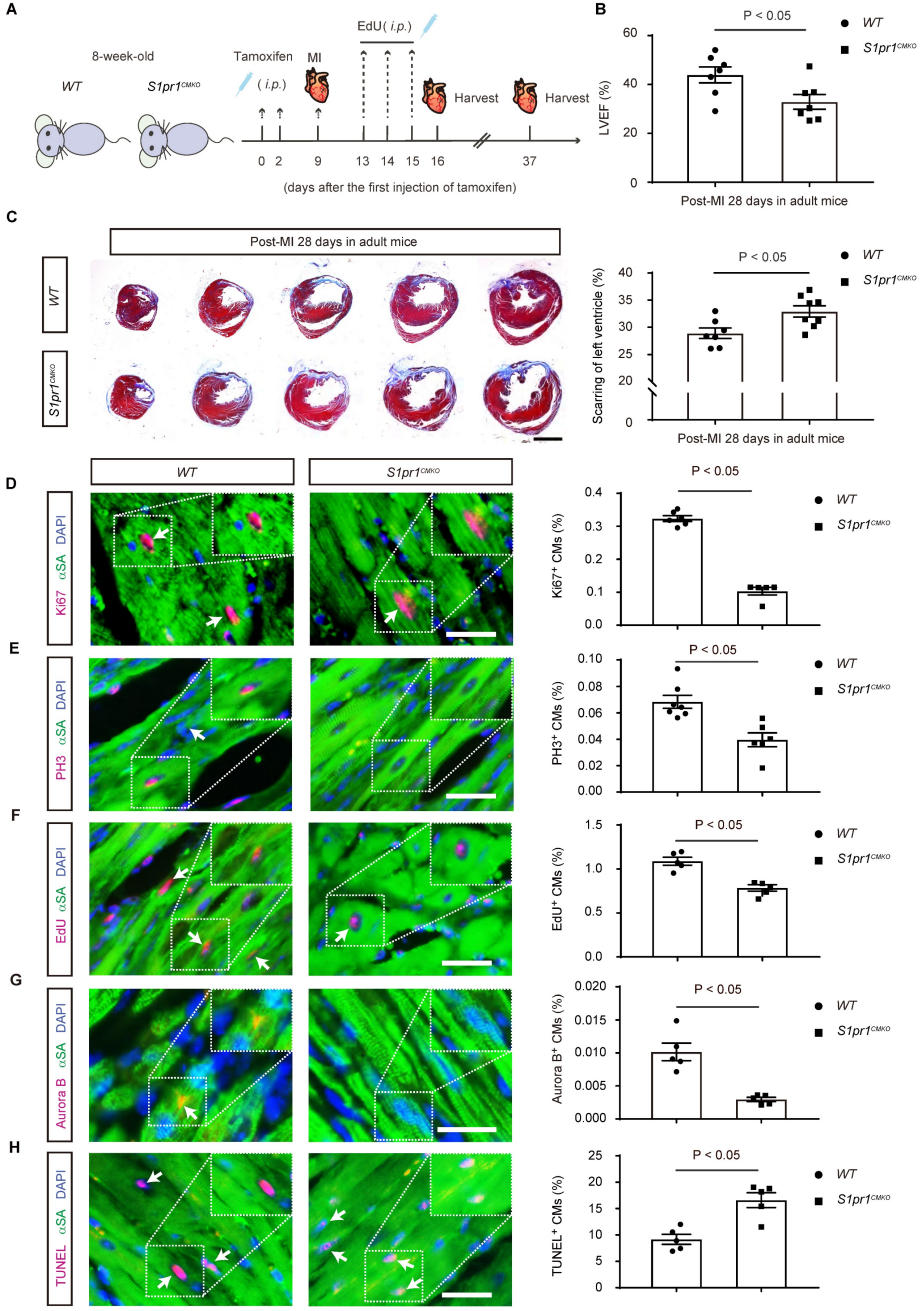
** The cardiomyocyte-specific loss of S1PR1 restrained heart regeneration and aggravated cardiac dysfunction after MI in adult mice. A**. Schematic diagram for experimental procedure. Tamoxifen (100 mg/kg, *i.p.*, every other day for total 2 injections) were administered to 8-week-old *WT* and *S1pr1^CMKO^* mice to induce specific deletion of* S1pr1* in CMs following with myocardial infarction (MI) at 7-day after tamoxifen administration. EdU were administered (50 mg/kg body weight,* i.p.*) daily for 3 consecutive days beginning from 4-day post-MI. Hearts tissues from sham-operated and MI-operated mice were harvested at designated post-MI time points (7-day, 28-day). **B.** Quantitative analysis of left ventricle ejection fraction (LVEF%) in *WT* and *S1pr1^CMKO^* mice which underwent the MI operation at age of 8 weeks were performed at 28-day post MI using echocardiography (n = 7). **C**. Representative images of Masson's Trichrome staining in hearts from *WT* and *S1pr1^CMKO^* mice which underwent the MI operation at age of 8 weeks. Hearts sections were collected from these mice at 28-day post MI, and quantification of the percentage of cardiac scar area in left ventricle (n = 5-7). **D-H**. Representative immunostaining images on peri-infarct sections for Ki67 (**D**), PH3 (**E**), EdU (**F**), Aurora B (**G**) and TUNEL (**H**) and α-SA positive cardiomyocytes of the border zone of injured hearts from* WT* and *S1pr1^CMKO^* mice which underwent the MI operation at age of 8 weeks. Hearts sections were collected from these mice at 7-day post MI (n = 5-8). The arrows indicate α-SA (green) cardiomyocytes positive for Ki67 (magenta), PH3 (magenta), Edu (magenta), Aurora B (magenta) or TUNEL (magenta). DAPI, nuclear staining (blue). Quantification of the percentage of Ki67^+^α-SA^+^, PH3^+^α-SA^+^, EdU^+^α-SA^+^, Aurora B^+^α-SA^+^ and TUNEL^+^α-SA^+^ cardiomyocytes on the right. α-SA, α-sacromeric actinin. PH3, phospho-histone H3. EdU, 5-ethynyl-2'-deoxyuridine. Data are represented as means ± S.E.M. P < 0.05 indicates significant statistical differences. n.s., no statistical significance. Unpaired Student's t-test (**B**-**H**). Scale bars: **C**, 2 mm; **D**-**H**, 25 µm.

To investigate whether the CM-specific *S1pr1* deletion influences adult cardiac regeneration and functions after heart injuries, we studied 8-week-old adult mice subjected to left anterior descending coronary artery ligation to induce MI (**Figure 3A**). Tamoxifen-induced CM-specific *S1pr1* knock-out did not affect heart morphology or cardiac functions in the sham group (**Figure S3A-B** and **Table S5**). Additionally, heart rhythm was unaffected by CM-specific *S1pr1* deletion in our mouse models (**Figure S3C-F**). Under sham operation conditions, CM-specific *S1pr1* knock-out mice displayed similar cardiomyocyte size and capillary vessel density as *WT* control mice (**Figure S3G-J**). However, our echocardiographic analysis revealed that *S1pr1^CMKO^* adult mice exhibited a significant reduction in left ventricular ejection fraction (LVEF%) at 4 weeks post-MI compared to control littermates (**Figure 3B** and **Table S5**). Furthermore, *S1pr1^CMKO^* mice showed larger fibrotic scar size as indicated by Masson's Trichrome staining (**Figure 3C**). The loss of *S1pr1* in CMs induced by tamoxifen in adult mice did not affect post-MI cardiac hypertrophy, as demonstrated by the heart weight to body weight ratio and WGA staining (**Figure S4A-C**), nor did it influence cardiac microvessel density, as shown by IB4 staining (**Figure S4D-E**). CM-specific *S1pr1* deletion significantly decreased cardiomyocyte proliferation at 7 days after MI, evidenced by reduced percentages of Ki67- , PH3-, and EdU-positive cardiomyocytes (**Figure 3D-F** and **Figure S5**). Aurora B immunostaining demonstrated that CM-specific* S1pr1* deletion significantly reduced CM cytokinesis in adult mice compared to* WT* mice at 7 days post-MI (**Figure 3G**). Additionally, more apoptotic cardiomyocytes were detected in *S1pr1^CMKO^* adult mice compared to *WT* mice at 7 days in infarct border zone after MI (**Figure 3H** and **Figure S6**). Collectively, these results indicate that CM-specific* S1pr1* deletion inhibits cardiac regeneration, leading to worse cardiac dysfunctions in post-MI adult mice.

### The cardiomyocyte-specific S1PR1 overexpression enhances heart regeneration and improves cardiac functions in neonatal mice after AR

**Figure 4:**
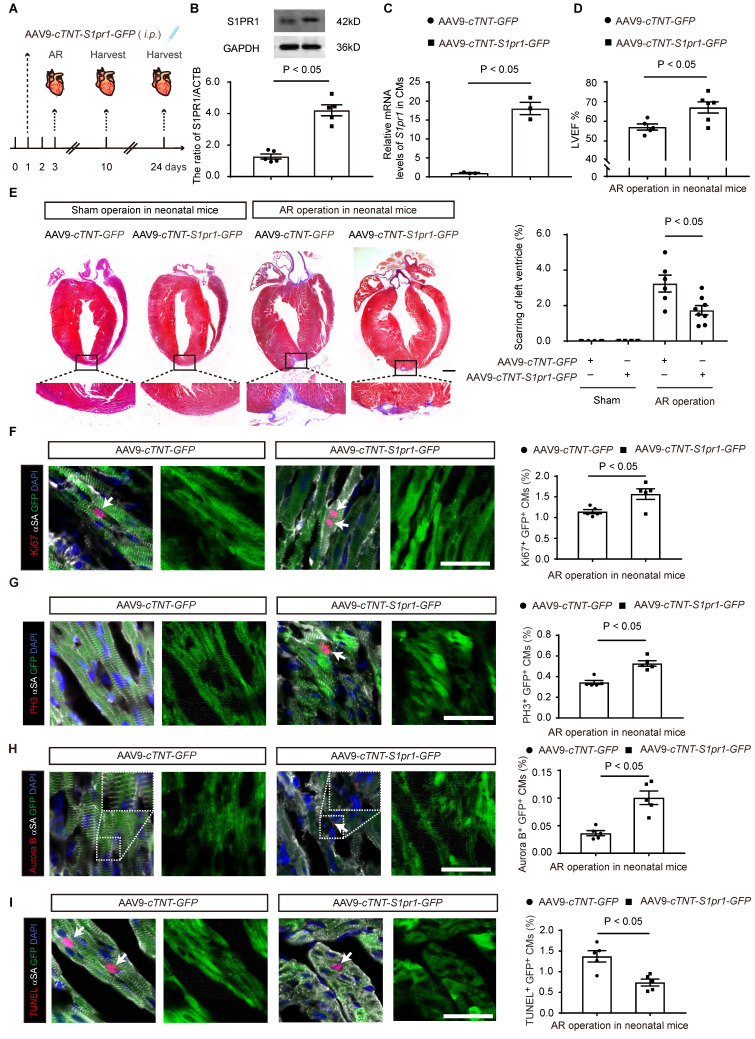
** The cardiomyocyte-specific S1PR1 overexpression enhances cardiac proliferation and improves cardiac functions in neonatal mice after AR. A**. Schematic diagram for experimental procedure. AAV9-*cTNT-S1pr1*-*GFP* driven by *cTnT* promoter were administered (8 x 10^9^ viral genome particles per mouse, *i.p.*) to postnatal day 1 (P1) neonatal pups to achieve cardiomyocyte (CM)-specific S1PR1 overexpression. Apical resection (AR) surgery was operated at postnatal day 3 (P3) and hearts tissue were harvested from sham-operated and AR-operated mice at designated post-AR time points (7-day, 21-day). **B**. Images of western blot and quantification of S1PR1 protein levels in CMs isolated from mice which underwent the AAV9-*cTNT-S1pr1*-*GFP* or AAV9-*cTNT-GFP* virus administration at postnatal day 1 (P1). Cardiomyocytes (CMs) were collected from these mice at 7-day post-infection (n = 4). **C.** Relative mRNA expression levels of *S1pr1* in CMs isolated from mice which underwent the AAV9-*cTNT-S1pr1*-*GFP* or AAV9-*cTNT-GFP* virus administration at postnatal day 1 (P1). Cardiomyocytes (CMs) were collected from these mice at 7-day post-infection (n = 3).** D**. Echocardiographic assessment of left ventricle ejection fraction (LVEF%) in AAV9-*cTNT-S1pr1*-*GFP* and AAV9-*cTNT-GFP* mice which underwent the AR operation at postnatal day 3 (P3) were performed at 21-day post AR using echocardiography (n = 5-6). **E**. Representative images of Masson's Trichrome staining of hearts from AAV9-*cTNT-S1pr1*-*GFP* and AAV9-*cTNT-GFP* neonatal mice which underwent the sham operation or the AR operation at postnatal day 3 (P3). Hearts sections were collected from these mice at 21-day post AR and quantification of the percentage of cardiac fibrotic scars in left ventricles (n = 4-8).** F-I**. Representative immunostaining images on heart apex sections for Ki67 (**F**), PH3 (**G**), Aurora B (**H**) or TUNEL (**I**) and α-SA positive cardiomyocytes within the border zone of injured hearts from AAV9-*cTNT-S1pr1*-*GFP* or AAV9-*cTNT-GFP* mice which underwent the AR operation at postnatal day 3 (P3). Heart sections were collected from these mice at 7-day post AR operation (n = 5). The arrows indicate α-SA (white) & GFP (green) cardiomyocytes positive for Ki67 (magenta), PH3 (magenta), Aurora B (magenta) or TUNEL (magenta). DAPI, nuclear staining (blue). Quantification of the percentage of Ki67^+^α-SA^+^GFP^+^, PH3^+^α-SA^+^GFP^+^, Aurora B^+^α-SA^+^GFP^+^ and TUNEL^+^α-SA^+^GFP^+^ cardiomyocytes on the right. α-SA, α-sacromeric actinin. PH3, phospho-histone H3. Data are represented as means ± S.E.M. P < 0.05 indicates significant statistical differences. n.s. no statistical significance. Unpaired Student's t-test (**B**-**D** and **F-I**). One-way ANOVA (**E**). Scale bars: **E**, 2 mm; **F-I**, 15 µm.

To further elucidate the role of CM-expressing S1PR1 for heart regeneration *in vivo*, we performed S1PR1 gain-of-function experiments in mice using AAV9-mediated *S1pr1* overexpression driven by the CM-specific promoter, *cTNT*. Neonatal mice were infected with AAV9-*cTNT*-*S1pr1*-*GFP* or AAV9-*cTNT*-*GFP* via intraperitoneal injection (*i.p*.) in P1 mice (**Figure 4A**). Our data indicated that the AAV9-*cTNT* system effectively delivered target genes to cardiomyocytes with an efficiency of approximately 64.70 ± 6.21% (mean ± S.E.M) (**Figure S7A-B**).

AAV9-*cTNT*-*S1pr1*-*GFP* significantly elevated the expression of S1PR1/*S1pr1* in isolated cardiomyocytes, as confirmed by western-blotting and RT-qPCR analysis (**Figure 4B-C**). As expected, CM-specific S1PR1 overexpression improved cardiac functions 3 weeks after AR, as evidenced by echocardiography analysis of LVEF% (**Figure 4D** and **Table S6**). Additionally, S1PR1 overexpression in cardiomyocytes resulted in a marked reduction in myocardial scar size after AR injuries in neonates (**Figure 4E**). We then assessed whether S1PR1 overexpression in cardiomyocytes was sufficient to enhance cardiomyocyte proliferation. Indeed, CM-specific S1PR1 overexpression showed a significant increase in the percentage of Ki67-, and PH3-positive cardiomyocytes (**Figure 4F-G**). Notably, an increased number of cardiomyocytes undergoing cytokinesis is observed in neonates infected with AAV9-*cTNT*-*S1pr1* after AR, as shown by Aurora B immunostaining (**Figure 4H**). Concurrently, a reduction in cardiomyocyte apoptosis was observed in neonates infected with AAV9-*cTNT*-*S1pr1* after AR (**Figure 4I**). Collectively, these results confirm that the CM-expressing S1PR1 plays a crucial role in regulating cardiomyocyte proliferation and apoptosis during cardiac regeneration following heart injuries.

### S1PR1 regulates AKT/mTORC1/CYCLIN D1 and BCL2 signaling pathways in cardiomyocytes

**Figure 5:**
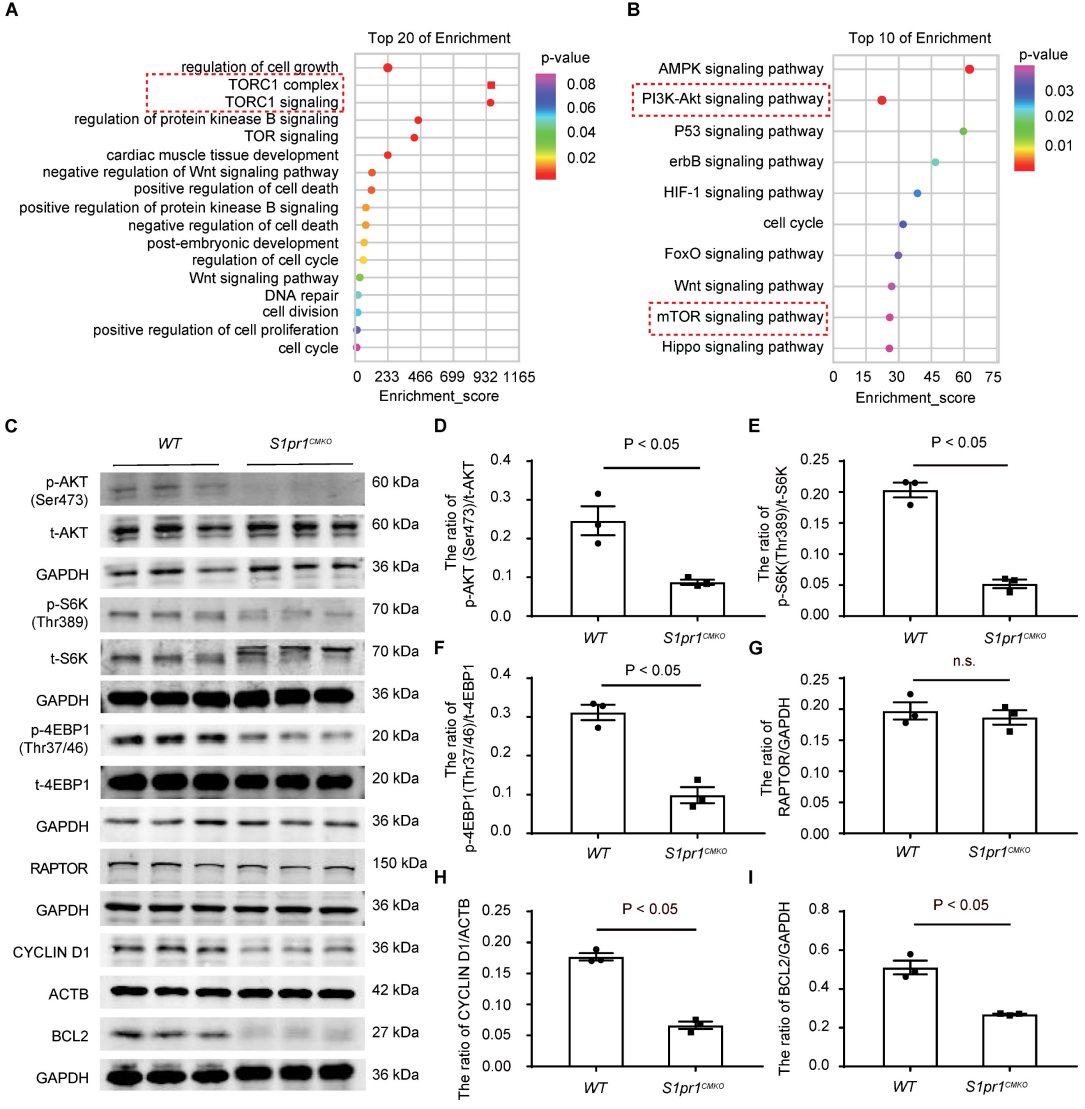
** AKT/mTORC1 signaling is identified as a key downstream of S1PR1 in cardiomyocyte to promote CM proliferation and heart regeneration. A-B**. Gene Ontology (GO) enrichment analysis (**A**) and Kyoto Encyclopedia of Genes and Genomes (KEGG) pathway analysis (**B**) were performed on heart tissues from *WT* and *S1pr1^CMKO^* neonatal mice which underwent the AR operation at postnatal day 3 (P3). Hearts were collected from these mice at 1-day post AR operation for gene expression microarray.** C-I**. Western blot analysis was performed to assess the protein levels of total and phosphorylated AKT (Ser473), total and phosphorylated S6K (Thr389), total and phosphorylated 4EBP1 (Thr37/46), RAPTOR, CYCLIN D1 and BCL2 protein levels in heart tissues from *WT* and *S1pr1^CMKO^* neonatal mice which underwent the AR operation at postnatal day 3 (P3). Hearts tissues were collected from these mice at 1-day post AR operation (**C**), with quantification of the ratio of p-AKT(Ser473) /t-AKT (**D**), the ratio of p-S6K (Thr389) /t-S6K (**E**), the ratio of p-4EBP1(Thr37/46) /t-4EBP1 (**F**), RAPTOR/GAPDH (**G**), CYCLIN D1/ACTB (**H**), and the ratio of BCL2/GAPDH (**I**) (n = 3). mTOR, mammalian target of rapamycin. S6K, ribosomal S6 kinase. 4EBP1, eIF4E-binding protein 1. RAPTOR, regulatory-associated protein of mammalian target of rapamycin. BCL2, B-cell lymphoma 2. ACTB, β-actin. The red dotted line represents the important signaling pathways related to PI3K-AKT/mTOR signaling. Data are represented as means ± S.E.M. P < 0.05 indicates significant statistical differences. n.s. no statistical significance. Unpaired Student's t-test (**D**-**I**).

To study the molecular mechanism by which CM-S1PR1 regulates cardiac regeneration, we performed gene expression analysis of heart tissues from* S1pr1^CMKO^* and *WT* neonates by RNA microarray. Gene Ontology (GO) analysis of differentially expressed genes revealed multiple downregulated genes related to cell growth, cardiac muscle cell development, positive regulation of cell death, regulation of cell cycle (**Figure 5A**). The GO and KEGG analysis showed that the enrichment of signaling pathways were related to PI3K-AKT and mTORC1 signaling pathways (**Figure 5A-B**). It has been shown that S1PR1 activated the AKT signaling pathway, which subsequently activates the mTOR pathway 35, 36. Our data showed that the phosphorylation levels of AKT (Ser473) were significantly reduced in *S1pr1^CMKO^* neonatal hearts compare to *WT* hearts, suggesting that CM-S1PR1 regulated AKT activity (**Figure 5C-I**). Recent studies have highlighted the crucial role of mTORC1 in heart and other organ regeneration 37-41. As previous investigation reported, the phosphorylation of mTOR itself is a controversial indicator of mTOR activity 42. Therefore, we used the phosphorylation of S6K (Thr389) and 4E-BP1 (Thr37/46), downstream targets of mTORC1, as reliable indicators of mTORC1 activity 42. Our results indicated that the phosphorylation levels of S6K (Thr389) and 4E-BP1 (Thr37/46) were significantly reduced in *S1pr1^CMKO^* neonates compared to *WT* littermates (**Figure 5C-I**). However, RAPTOR expression, a critical component of mTORC1, did not differ between* S1pr1^CMKO^* and *WT* neonates (**Figure 5C** and** 5G**), suggesting that S1PR1 regulated mTORC1 activity rather than mTORC1 expression. Additionally, the mTORC1 pathway is known to promote the expression of positive cell cycle regulators, including CYCLIN D1 34. Our results showed reduced CYCLIN D1 expression in *S1pr1^CMKO^* hearts, as determined by western-blotting analysis (**Figure 5C** and** 5H**), indicating that S1PR1/AKT/mTORC1/CYCLIN D1 signaling pathway may regulate cell cycle and cell proliferation during the process of cardiac regeneration. Furthermore, previous studies have reported that the AKT signaling pathway regulated the expression of numerous "cell death genes", including anti-apoptotic gene *BCL2* 43. As expected, the expression of BCL2 was significantly reduced in *S1pr1^CMKO^* neonates as shown by western-blotting analysis (**Figure 5C** and **5I**). These results suggested that S1PR1/AKT/mTORC1/CYCLIN D1 and BCL2 signaling pathway may be responsible for the effect of S1PR1 on cardiomyocyte proliferation and cell apoptosis.

### S1PR1 inhibits cardiomyocyte apoptosis via AKT/BCL2 signaling pathways

We further investigated whether S1PR1/AKT/BCL2 signaling pathway regulates cardiomyocyte apoptosis and cell survival. We treated cardiomyocytes with S1PR1 agonist, SEW2871, and subjected to apoptosis induced by overnight serum deprivation and hypoxia for 24 hours followed by reoxygenation for 12 hours, as established in prior studies 27-29.

**Figure 6:**
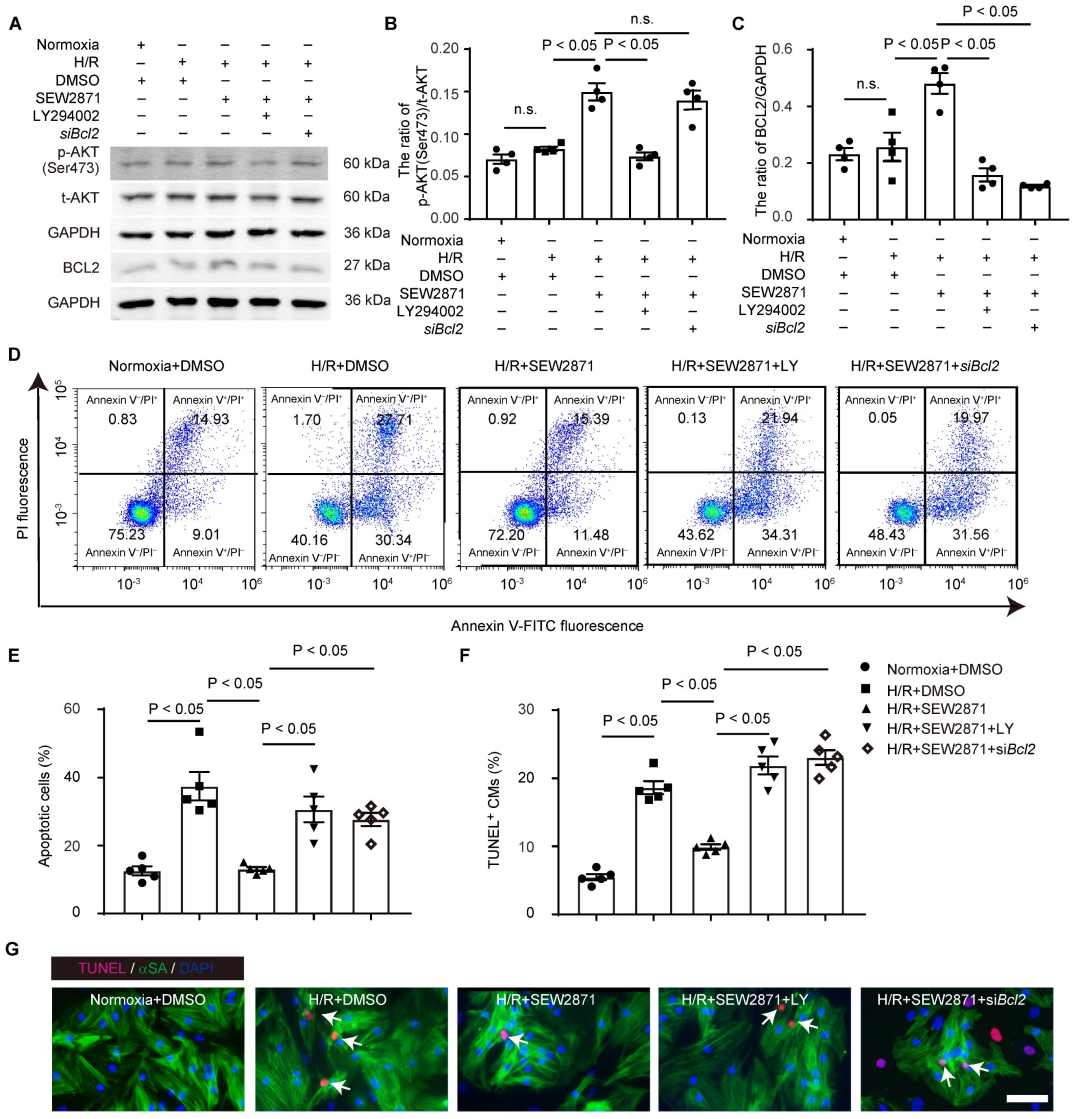
**S1PR1 inhibits cardiomyocyte apoptosis via AKT/BCL2 signaling pathways. A-C**. Western blot analysis was conducted to assess the levels of total and phosphorylated AKT (Ser473) and BCL2 protein in neonatal mouse cardiomyocytes (NMCMs) treated with or without SEW2871 under the normoxia or 24h-hypoxia/12h-reoxygenation condition (**A**). Quantification of the ratio of p-AKT(Ser473)/t-AKT (**B**), the ratio of BCL2/GAPDH (**C**) (n = 4).** D-E**. Flow cytometry analysis using Annexin V/PI staining was utilized to evaluate apoptosis in NMCMs treated with or without SEW2871, LY294002 or *Bcl2-siRNA* under 24h-hypoxic/12h-reoxygenation condition (**D**) and quantification of the percentage of apoptotic NMCMs (Annexin V FITC^+^/PI^-^) (**E**) (n = 5). **F-G**. Representative immunostaining images of TUNEL positive and α-SA positive NMCMs treated with or without SEW2871, LY294002 or *Bcl2-siRNA* under 24h-hypoxia/12h-reoxygenation condition (n = 5) (**G**). The arrows indicate α-SA (green) cardiomyocytes positive for TUNEL (magenta). DAPI, nuclear staining (blue). Quantification of the percentage of TUNEL^+^α-SA^+^ cardiomyocytes (**F**). H/R, 24h-hypoxia/12h-reoxygenation condition. BCL2, B-cell lymphoma 2. SEW2871, S1PR1 agonist. LY294002 (LY), AKT inhibitor. *siBcl2*, *Bcl2-siRNA*. Data are represented as means ± S.E.M. P < 0.05 indicates significant statistical differences. n.s., no statistical significance. Scale bar: **G**, 50 µm. One-way ANOVA (**B**-**C** and **E**-**F**).

Western-blotting analysis showed that the S1PR1 agonist increased AKT activity and BCL2 expression under hypoxia/reoxygenation conditions (**Figure 6A-C**). Conversely, the AKT inhibitor, LY294002, blocked this enhancement of AKT activity and BCL2 expression, confirming that S1PR1 regulates AKT activation, which subsequently upregulates BCL2 expression in cardiomyocytes (**Figure 6A-C**). Both flow cytometric analysis of CM apoptosis and TUNEL staining revealed that S1PR1 activation significantly reduced cardiomyocyte apoptosis induced by hypoxia/reoxygenation (**Figure 6D-G**). Furthermore, inhibition of AKT or BCL2 reversed the protective effect of S1PR1 agonist on CM apoptosis (**Figure 6D-G**). These data collectively indicate that S1PR1 inhibits cardiomyocyte apoptosis via AKT/BCL2 signaling pathway.

### S1PR1 increases cardiomyocyte proliferation via AKT/mTORC1/CYCLIN D1 signaling pathways

**Figure 7:**
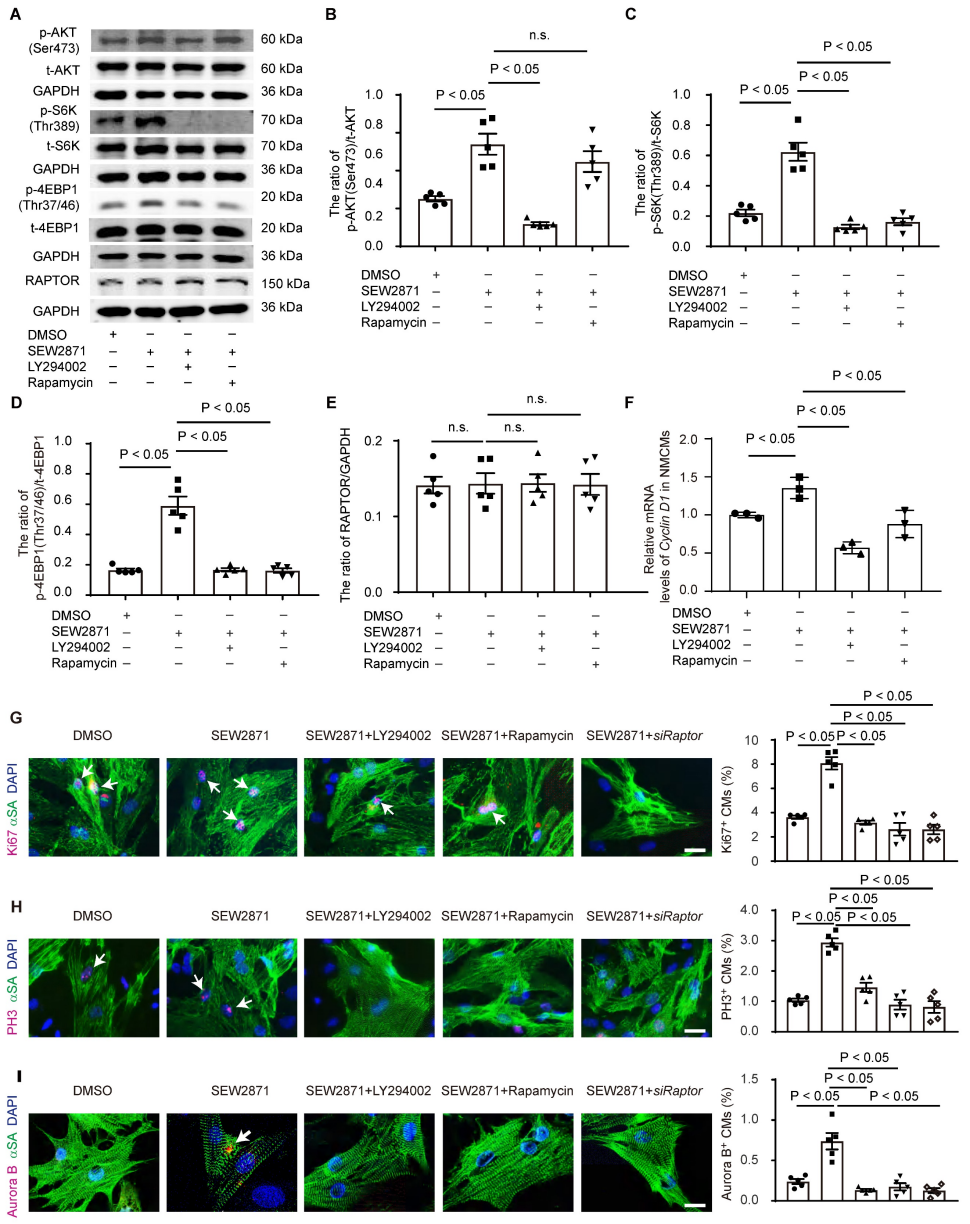
** S1PR1 increases cardiomyocyte proliferation via AKT/ mTORC1/CYCLIN D1 signaling pathways. A-E**. Western blot analysis was conducted to assess of the expression levels of total and phosphorylated AKT (Ser473), total and phosphorylated S6K (Thr389), total and phosphorylated 4EBP1 (Thr37/46) and RAPTOR protein levels in neonatal mouse cardiomyocytes (NMCMs) with or without SEW2871 in the presence or absence of rapamycin or LY294002 (**A**) and quantification of the ratios of phosphorylated AKT (Ser473) to total AKT, p-AKT (Ser473)/t-AKT (**B**), the ratios of phosphorylated S6K (Thr389) to total S6K, p-S6K(Thr389)/t-S6K (**C**), the ratios of phosphorylated 4EBP1 (Thr37/46) to total 4EBP1, p-4EBP1(Thr37/46)/t-4EBP1 (**D**) and RAPTOR protein levels (**E**) (n = 5). **F**. The mRNA expression levels of *Cyclin D1* were determined by RT-qPCR in NMCMs treated with or without SEW2871 in the presence or absence of rapamycin or LY294002 (n = 3). **G-I**. Representative immunostaining images of Ki67 (**G**), PH3 (**H**) or Aurora B (**I**) and α-SA positive NMCMs treated with or without SEW2871 in the presence or absence of rapamycin, LY294002 or *Raptor siRNA*. The arrows indicate α-SA (green) cardiomyocytes positive for Ki67 (magenta), PH3 (magenta) or Aurora B (magenta). DAPI, nuclear staining (blue). Quantification of the percentage of Ki67^+^α-SA^+^, PH3^+^α-SA^+^ or Aurora B^+^α-SA^+^ cardiomyocytes on the right (n = 5). α-SA, α-sacromeric actinin. PH3, phospho-histone H3. S6K, ribosomal S6 kinase. 4EBP1, eIF4E-binding protein 1. RAPTOR, regulatory-associated protein of mammalian target of rapamycin. SEW2871, S1PR1 agonist. LY294002 (LY), AKT inhibitor. Rapamycin, mTOR inhibitor. *siRaptor, Raptor siRNA*. Data are represented as means ± S.E.M. P < 0.05 indicates significant statistical differences. n.s. no statistical significance. Scale bar: **G**-**H**, 25 µm. **I**, 15 µm; One-way ANOVA (**B**-**I**).

To further elucidate the role of S1PR1/AKT/mTORC1/CYCLIN D1 signaling pathway in cardiomyocyte proliferation, we performed *in vitro* experiments using neonatal cardiomyocytes. Neonatal cardiomyocytes were treated with S1PR1 agonist, SEW2871, with or without AKT antagonist, LY294002, or mTORC1 antagonist, rapamycin. SEW2871 enhanced the activity of both AKT and mTORC1, as evidenced by changes in phosphorylation levels of AKT (Ser473), S6K (Thr389), and 4E-BP1 (Thr37/46), and the AKT antagonist inhibited S1PR1-mediated mTORC1 activity in cardiomyocytes (**Figure 7A-D**), suggesting S1PR1 triggered mTORC1 activation via AKT signaling pathway. We next stimulated neonatal cardiomyocytes with S1P, and our results showed that S1P stimulation increased the activity of mTORC1 in cardiomyocytes compared to vehicle control, while the knockdown of *S1pr1* decreased their activity (**Figure S8A-C**). As expected, rapamycin efficiently inhibited the activity of mTORC1 *in vitro* cell cultures (**Figure 7A-D**). Interestingly, our data showed a significant increase in the phosphorylation of AKT (Ser473) levels following short-term rapamycin treatment (**Figure 7A-B**), which is consistent with previous studies that inhibition of mTORC1 lifts the feedback inhibition on mTORC2, leading to enhanced mTORC2 activity, which consequently increased phosphorylation of AKT (S473) 44, 45. It has been known that TSC-1 negatively regulated mTORC1 activity and that TSC-1 knockdown induced constitutive mTORC1 activation 46. We thus utilized *Tsc-1 siRNA* to knockdown *Tsc-1* in neonatal cardiomyocytes, and our results indicated that *Tsc-1 siRNA* reversed the diminished mTORC1 activity observed in *S1pr1* knockdown cardiomyocytes (**Figure S8A-D**). Additionally, neither total AKT protein levels nor RAPTOR expression were affected by S1PR1 agonist or *S1pr1* knockdown (**Figure 7A-E** and **Figure S8A-D**), suggesting that S1PR1 regulates AKT/mTORC1 signaling pathway without influencing the expression of AKT/mTORC1 itself. Furthermore, SEW2871 increased the expression of *Cyclin D1*, while inhibition of AKT/mTORC1 reduced its expression, indicating that S1PR1 enhances *Cyclin D1* expression through AKT/mTORC1 pathway (**Figure 7F**). As expected, SEW2871 promoted neonatal cardiomyocyte proliferation and cardiomyocytes cytokinesis *in vitro* (**Figure 7G-I**), an effect that was blocked by AKT/mTORC1 inhibitors or* Raptor siRNA*, as demonstrated by Ki67, PH3 and Aurora B staining in cardiomyocytes (**Figure 7G-I**). Conversely, S1PR1 knockdown inhibited cardiomyocyte proliferation, while *Tsc-1* knockdown promoted cell proliferation in *S1pr1* knockdown cardiomyocytes (**Figure S8E-G**). These findings underscore the causal link between S1PR1 and mTORC1 signaling-mediated cardiomyocyte proliferation.

Prior studies have demonstrated that mTORC1 regulates mitochondrial dynamics by modulating DRP1 47, a key regulator of mitochondrial fission and homeostasis 48. Therefore, we hypothesized that the S1PR1/mTORC1/DRP1 axis may play a role in cardiomyocyte mitochondrial dynamics.

Our data revealed that activation of S1PR1 with the agonist SEW2871 significantly reduced DRP1 phosphorylation, indicative of enhanced DRP1 activity (**Figure S9A**). This effect was abrogated by mTORC1 inhibition using rapamycin or* siRNA* targeting* Raptor*, a critical mTORC1 component, demonstrating that S1PR1-regulated DRP1 activation is mTORC1-dependent (**Figure S9A**). Consistent with the previous report 49, hypoxia-induced stress led to excessive mitochondrial hyperfission, mitochondrial membrane potential depolarization, and dysfunction (**Figures S9B-E**). Our data further showed that S1PR1 activation mitigated hypoxia-induced mitochondrial hyperfission and preserved mitochondrial membrane potential (**Figures S9B-E**). However, these protective effects were abolished by mTORC1 inhibition, further implicating the S1PR1/mTORC1 pathway in maintaining mitochondrial homeostasis (**Figures S9B-E**). It has been well known that cytochrome C release from damaged mitochondria is a critical step in cell apoptosis 50. Furthermore, we observed increased cytoplasmic cytochrome C levels in CMs following hypoxia/reoxygenation injury (**Figure S9F**). Activation of S1PR1 inhibited cytochrome C release into the cytoplasm (**Figure S9F**), thereby reducing the apoptotic trigger. This inhibitory effect was reversed by rapamycin and* Raptor siRNA*, confirming that the S1PR1-mediated suppression of cytochrome C leakage is mTORC1-dependent (**Figure S9F**). Our findings suggest that CM-S1PR1 not only enhances cardiomyocyte proliferation via mTORC1/CYCLIN D1 signaling pathway, but also regulates mitochondrial homeostasis to reduce apoptotic signaling via the mTORC1/DRP1 axis.

**Figure 8:**
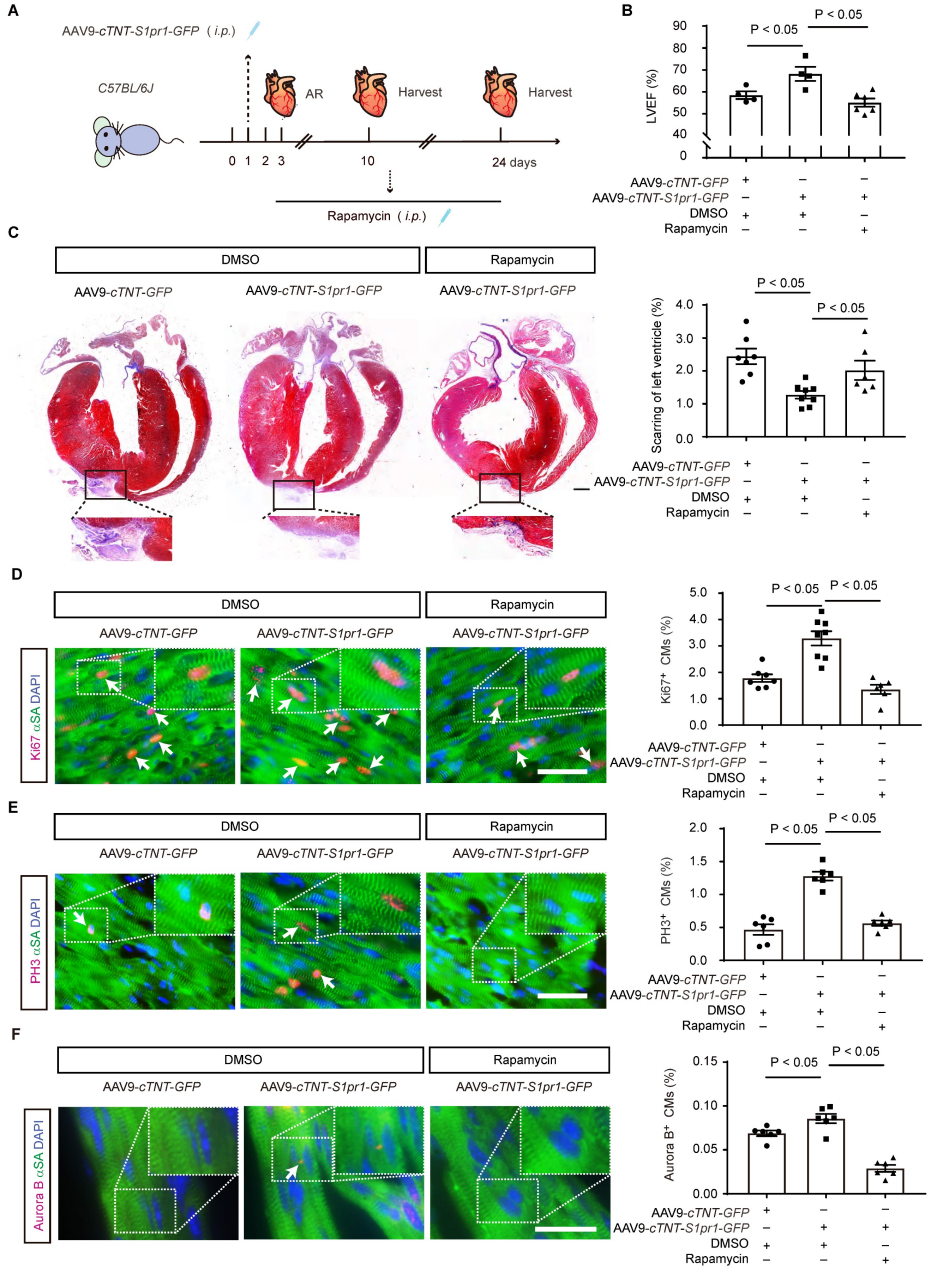
** S1PR1 increases cardiomyocyte proliferation via mTORC1 signaling pathways in neonatal mice. A**. Schematic diagram for experimental procedure. AAV9-*cTNT-S1pr1*-*GFP* driven by *cTnT* promoter were administered (8 × 10^9^ viral genome particles per mouse, *i.p.*) to postnatal day 1 (P1) neonatal pups to achieve cardiomyocyte (CM)-specific S1PR1 overexpression. Apical resection (AR) surgery was operated at postnatal day 3 (P3) followed by rapamycin (2 mg/kg body weight, every day,* i.p*.) administration. Hearts tissues from sham-operated and AR-operated mice were harvested at designated post-AR time points (7-day, 21-day).** B**. Quantitative assessment of left ventricle ejection fraction (LVEF%) in AAV9-*cTnT*-*S1pr1*-*GFP* and AAV9-*cTnT*-*GFP* neonatal mice which underwent the AR operation at postnatal day 3 (P3) were performed at 21-day post AR with or without rapamycin treatment (n = 4-6). **C**. Representative images of Masson's Trichrome staining in AAV9-*cTnT*-*S1pr1*-*GFP* and AAV9-*cTnT*-*GFP* neonatal mice which underwent the AR operation at postnatal day 3 (P3) with or without rapamycin treatment. Hearts sections were collected from these mice at 21-day post AR and quantification of the percentage of cardiac scar area in left ventricles on the right (n = 6-8). **D-F**. Representative immunostaining images on heart sections for Ki67 (**D**), PH3 (**E**) or Aurora B (**F**) and α-SA positive cardiomyocytes within the border zone of injured hearts from AAV9-*cTNT-S1pr1*-*GFP* or AAV9-*cTNT-GFP* neonatal mice which underwent the AR operation at postnatal day 3 (P3) with or without rapamycin treatment. Hearts sections were collected from these mice at 7-day post AR (n = 6-8). The arrows indicate α-SA (green) cardiomyocytes positive for Ki67 (magenta), PH3 (magenta) or Aurora B (magenta). DAPI, nuclear staining (blue). Quantification of the percentage of Ki67^+^α-SA^+^, PH3^+^α-SA^+^ or Aurora B^+^α-SA^+^ cardiomyocytes on the right. α-SA, α-sacromeric actinin. PH3, phospho-histone H3. Rapamycin, mTOR inhibitor. Data are represented as means ± S.E.M. P < 0.05 indicates significant statistical differences. One-way ANOVA (**B**-**F**). Scale bars: **C**, 2 mm; **D**-**F**, 15 µm.

To further investigate whether the effect of CM-S1PR1 on cardiomyocyte proliferation is dependent on mTORC1 signaling* in vivo*, neonates were treated with rapamycin (**Figure 8A**). As previously mentioned, AAV9-*cTNT*-*S1pr1*-*GFP* enhanced cardiac functions 3 weeks after AR, an effect that was reversed by rapamycin treatment* in vivo* (**Figure 8B** and **Table S7**). Rapamycin also reversed the inhibition of cardiac scar formation caused by CM-specific S1PR1 overexpression following heart injuries (**Figure 8C**). Moreover, rapamycin did not exacerbate the detrimental effects of cardiomyocyte-specific S1PR1 deletion on cardiac dysfunction and regeneration after AR heart injuries (**Figure S10A-G** and **Table S8**). These results indicate that the cardioprotective effects of S1PR1 overexpression are dependent on the mTORC1 pathway. To assess whether mTORC1 signaling pathway is necessary for the effect of CM-expressing S1PR1 on cardiomyocyte proliferation, mTORC1 inhibition by rapamycin was shown to reduce the increase in Ki67-, PH3- and Aurora B-positive cardiomyocytes induced by S1PR1 overexpression (**Figure 8D-F**). This suggests that the mTORC1 signaling pathway contributes to the S1PR1-indcued enhancement of cardiomyocyte proliferation *in vivo*. Both our *in vitro* experiment and *in vivo* investigations confirm the crucial role of S1PR1 in cardiomyocyte proliferation via AKT/mTORC1 pathway.

### S1PR1 overexpression and activation in cardiomyocytes promotes cardiac proliferation and improves cardiac functions after MI in adult mice

**Figure 9:**
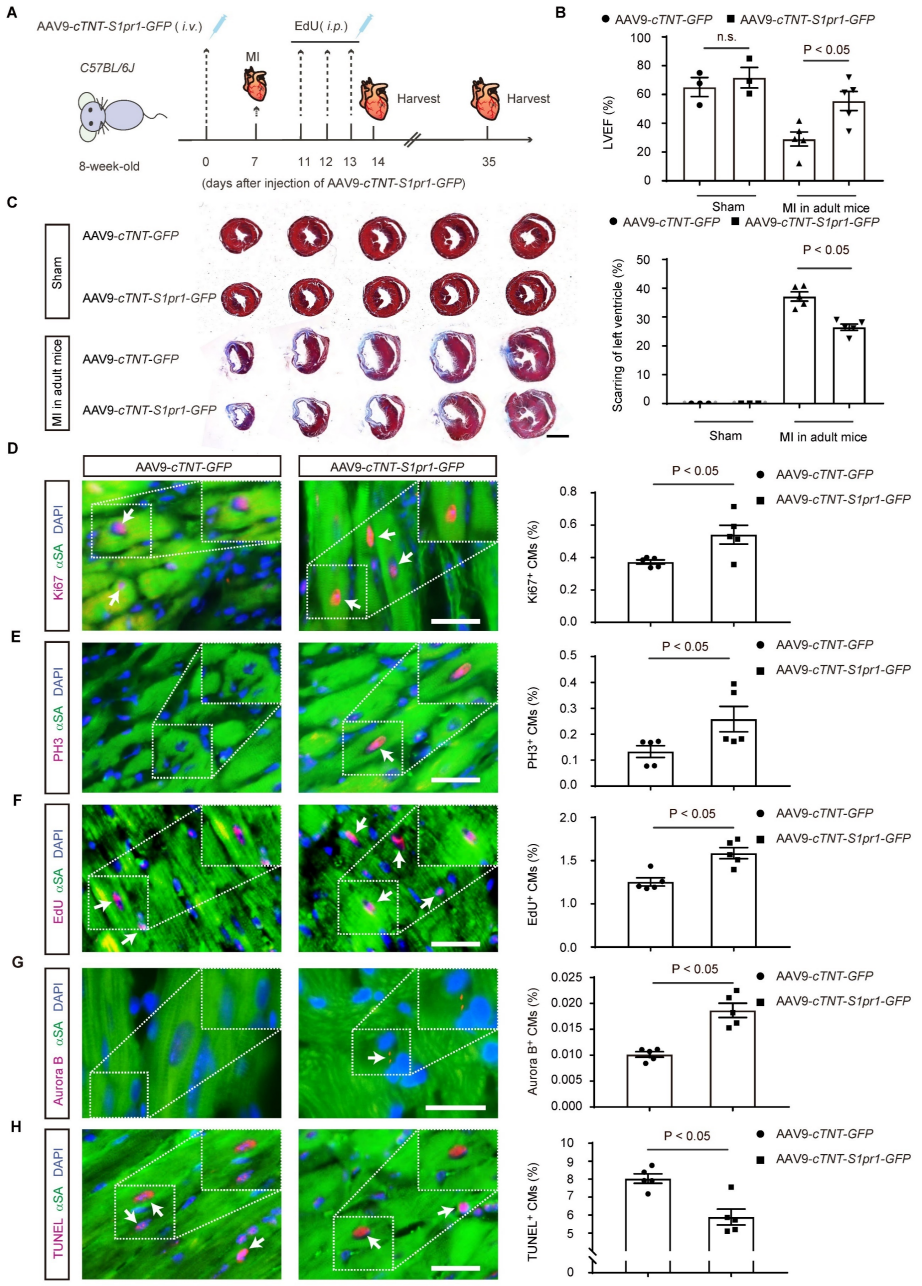
** Overexpression of S1PR1 in cardiomyocytes promotes cardiac proliferation and improve cardiac functions after MI in adult mice. A**. Schematic diagram for experimental procedure. AAV9-*cTNT-S1pr1*-*GFP* driven by *cTnT* promoter were administered (4 × 10^11^ viral genome particles per mouse, *i.p.*) to 8-week-old mice to achieve cardiomyocyte (CM)-specific S1PR1 overexpression followed by myocardial infarction (MI) surgery at 7-day after AAV administration and EdU was administered at 6.5-day after MI. Hearts tissues from sham-operated and AR-operated mice were harvested at 7-day and 28-day after MI. **B**. Echocardiographic quantification of left ventricle ejection fraction (LVEF%) in AAV9-*cTnT*-*S1pr1*-*GFP* and AAV9-*cTnT*-*GFP* adult mice which underwent the MI operation were performed at 28-day post MI using echocardiography (n = 5). **C**. Representative images of Masson's Trichrome staining of AAV9-*cTnT*-*S1pr1*-*cTnT*-*GFP* and AAV9-*cTnT*-*GFP* mice which underwent the sham operation or MI operation at the age of 8 weeks. Hearts sections were collected from these mice at 28-day post MI and quantification of the percentage of cardiac scar area in left ventricles on the right (n = 3-5). **D-H**. Representative immunostaining images on peri-infarct sections for Ki67 (**D**), PH3 (**E**), EdU (**F**), Aurora B (**G**) and TUNEL (**H**) and α-SA positive cardiomyocytes of injured hearts from AAV9-*cTNT-S1pr1*-*GFP* or AAV9-*cTNT-GFP* mice which underwent MI operation at postnatal day 56 (P56). Hearts sections were collected from these mice at 7-day post MI. The arrows indicate α-SA (green) cardiomyocytes positive for Ki67 (magenta), PH3 (magenta), EdU (magenta), Aurora B (magenta) or TUNEL (magenta). DAPI, nuclear staining (blue). Quantification of the percentage of Ki67^+^α-SA^+^, PH3^+^α-SA^+^, EdU^+^α-SA^+^, Aurora B^+^α-SA^+^ and TUNEL^+^α-SA^+^ cardiomyocytes on the right (n = 5). α-SA, α-sacromeric actinin. PH3, phospho-histone H3. EdU, 5-ethynyl-2'-deoxyuridine. Data are represented as means ± S.E.M. P < 0.05 indicates significant statistical differences. n.s., no statistical significance. Unpaired Student's t-test (**B** and **D**-**H**). One-way ANOVA (**C**). Scale bars: **C**, 2 mm; **D**-**H**, 25 µm.

We next investigated whether CM-specific overexpression of S1PR1 could promote cardiac regeneration and improve cardiac function after MI in adult mice. To achieve this, AAV9-*cTNT-S1pr1*-*GFP* was intravenously administered to specifically overexpress S1PR1 in adult CMs (**Figure 9A** and** S11A**). Our results showed that AAV9-*cTNT*-*S1pr1*-*GFP* significantly increased *S1pr1*/S1PR1 expression levels in adult cardiomyocytes, as confirmed by RT-qPCR and western-blotting analysis (**Figure S11B-D**). As anticipated, CM-specific overexpression of S1PR1 markedly enhanced left ventricular myocardial contractility (**Figure 9B** and **Table S9**) and reduced the fibrotic scar size following MI (**Figure 9C**). More importantly, an increase in proliferative cardiomyocytes was observed in mice infected with AAV9-*cTNT*-*S1pr1*-*GFP*, as shown by immunostaining of Ki67, PH3, EdU, and Aurora B in adult post-MI cardiac tissues (**Figure 9D-G** and **Figure S12**). Notably, S1PR1 overexpression in cardiomyocytes significantly reduced cardiomyocyte apoptosis in the infarct border zone, as indicated by TUNEL staining (**Figure 9H** and **Figure S13**). Rapamycin reversed the enhancing effect of S1PR1 overexpression on cardiac regeneration and function, suggesting a crucial role of S1PR1 in cardiac repair is dependent on mTORC1 pathway (**Figure S11E-J** and **Table S10**).

Our prior investigation demonstrated that pharmacological activation of S1PR1 by SEW2871 (5 mg/kg/day for 4 weeks) significantly reduced fibrotic scar size and enhanced left ventricular myocardial contractility following myocardial infarction (MI) 20. Furthermore, we showed that cardiac endothelial cell-expressing S1PR1 promoted reparative macrophage proliferation, contributing to the protective effects of SEW2871 on myocardial infarction 20. To further investigate the regenerative effects of SEW2871 on cardiomyocytes in adult mice, we assessed the capability of cardiomyocyte proliferation in SEW2871-treated mice after MI. Our data showed that SEW2871 treatment enhanced cardiomyocyte proliferation in adult post-MI mice, as evidenced by increased immunostaining for Ki67, phospho-histone H3 (PH3), EdU, and Aurora B in cardiac tissues (**Figure S14A-D**).

Taken together, these findings suggest that both overexpression of S1PR1 in cardiomyocytes via AAV9-*cTNT*-*S1pr1*-*GFP* and pharmacologic activation of S1PR1 via SEW2871 enhances cardiomyocyte proliferation and consequently improves cardiac repair and cardiac functions following MI in adult mice. These approaches present promising therapeutic targets for myocardial infarction and heart failure.

## Discussion

Lower vertebrates, such as zebrafish and newts, retain the capability to regenerate their hearts 51-53, and some neonatal mammals, including mice and pigs, exhibit efficient regenerative potential during the early postnatal period 7. However, adult mammals lack the sufficient capacity to regenerate their hearts and restore cardiac functions following injury 54. Investigating the molecular mechanisms underlying cardiac regeneration is crucial for advancing our understanding of the intricate regulatory processes involved, potentially uncovering potential therapeutic avenues for treating heart failure.

S1P is a bioactive lipid and displays versatile functions mediated by its different S1P receptors (S1PRs) across various organs and cell types 10. It has been shown that S1P-S1PRs axis plays a key role in regulating both physiological and pathological processes of cardiovascular system 55. Among the five G protein-coupled receptors (S1PR1-S1PR5) that exhibit high affinity for S1P, S1PR1, S1PR2, and S1PR3 are predominantly expressed in cardiovascular tissues. In contrast, S1PR4 is primarily expressed in lymphatic system, and S1PR5 is mainly present in the immune and nervous systems 55, 56. Each S1P receptor couples with specific G protein α subunits, resulting in diverse cellular responses 57. S1PR1 exclusively binds to Gαi, whose activation involves multiple signaling pathways, including ERK1/2, AKT, and mTORC1 signaling 55, 57. Sekiguchi *et al*. reported no significant changes in cardiac myocyte size following S1P stimulation 58, whereas Robert *et al*. demonstrated significant enhancement of cardiomyocyte hypertrophy via the activation of S1PR1/Gαi, leading to downstream signaling via ERK1/2, p38, JNK, and AKT pathways 59. Notably, Liu *et al*. showed that FTY720, an activator of S1PR1, S1PR3, S1PR4, and S1PR5, could reverse pressure overload-induced cardiac hypertrophy and fibrosis while improving cardiac performance 60. Furthermore, S1P-mediated inhibition of cardiomyocyte apoptosis was found to dependent on downstream activation of AKT through S1PR1 61. *Ex vivo* experiments by Lecour *et al*., using isolated perfused rat and mouse hearts, demonstrated that S1P displayed the cardioprotective effects and reduced infarct size after heart regional reischemia/reperfusion (I/R) injury 62. Similar protective effects of S1P were obtained in isolated perfused mouse hearts subjected to global heart I/R injury 63. These investigations collectively underscore the important role of the S1P-S1PRs signaling pathway in the regulation of cardiac functions.

Among the five types of S1P receptors, sphingosine 1-phosphate receptor 1 (S1PR1) is predominantly expressed in both cardiomyocytes (CMs) and vascular endothelial cells (ECs) in the heart 56. Our previous reports revealed that EC-specific deletion of *S1pr1* inhibited the proliferation of reparative F4/80^+^Ly6c^low^ macrophages, exacerbating pathological cardiac remodeling and worsening cardiac dysfunction following myocardial infarction in mice, demonstrating a key role of EC-expressing S1PR1 for cardiac homeostasis 20. Besides the effect of EC-S1PR1 on cardiovascular system, cardiomyocyte-expressing S1PR1 has been shown to regulate cardiac development and functions 56. Clay *et al*. used *Nkx2.5*-cre and *Mlc2a*-cre mice to generate cardiomyocyte conditional knockout of *S1pr1* at the embryonic stages 17. Clay *et al*.'s investigations revealed that mice with CM-specific deletion of *S1pr1* displayed ventricular septal defects, which caused perinatal death in 68% of these mutant mice; however, the remaining mutant mice survived to adulthood 17. Jorgensen *et al.* further reported that these adult survivors of embryonic cardiomyocyte S1PR1 deletion displayed cardiac hypertrabeculation consistent with ventricular noncompaction 64. In contrast to previous studies that deleted S1PR1 during embryonic development 17, 64, we induced cardiomyocyte-specific S1PR1 deletion postnatally using tamoxifen. Under our experimental conditions, this postnatal deletion did not affect the morphology or function of the hearts. These findings suggest that the induction of cardiomyocyte-specific S1PR1 knockout after birth may not significantly influence heart development and growth within the observed time frame. Keul *et al*. bred α-myosin heavy chain-Cre mice (*αMHC*-Cre) with *S1pr1^flox/flox^* mice to obtain CM-specific *S1pr1* knock-out mice, *αMHC*-Cre;*S1pr1^flox/flox^* mice, and reported that *αMHC*-Cre;*S1pr1^flox/flox^* began to die prematurely from 34 weeks on 18. Their data showed that cardiac function is normal within 36 weeks and impaired at 48 weeks in *αMHC*-Cre;*S1pr1^flox/flox^* mice, which displayed a reduction in diastolic and systolic Ca^2+^ concentrations that were secondary to reduced intracellular Na^+^ and caused by suppressed activity of the sarcolemmal Na^+^/H^+^ exchanger NHE-1 18. They further reported that CM-S1PR1 played a role in S1P-mediated ischemic preconditioning, whereas there were no significant differences in infarct size between the *αMHC*-Cre;*S1pr1^flox/flox^* mice and *WT* control groups under basal conditions 18. In their study 18, S1PR1 was deleted starting from the embryonic stage. In contrast, our study employed a tamoxifen-inducible Cre system to delete S1PR1 specifically in cardiomyocytes after birth, thereby avoiding any impact on embryonic development. Under our experimental conditions and observation period, we did not observe significant cardiac fibrosis or dysfunction in *S1pr1^CMKO^* mice in the absence of additional heart injuries. Cannavo *et al*.'s investigation revealed a direct interaction between S1PR1 and the β1 adrenergic receptor (β1AR), which stimulation is tightly associated with the pathology of heart failure (HF). Stimulation of β1AR led to the downregulation of S1PR1 on cell membrane, while S1PR1 activation resulted in the downregulation of β1AR 65. Their study utilized rAAV6-S1PR1 gene therapy to overexpress S1PR1 in the heart, resulting in significantly improved cardiac function after myocardial infarction (MI) in adult rats 65. Their approach involved overexpressing S1PR1 in the entire heart tissue *in vivo*, while our study induced cardiomyocyte-specific S1PR1 knockout or overexpression* in vivo*, focusing primarily on the effects of CM-S1PR1 on cardiac regeneration in a neonatal AR model. Although we concentrated on this specific aspect, we did not exclude the potential mechanism by which S1PR1 regulates cardiac regeneration through its interaction with β1-adrenergic receptors (β1AR), which is an intriguing area warranting further investigation. These investigations demonstrated the essential role of cardiomyocyte-expressing S1PR1 in regulating cardiac homeostasis and functions. However, the impact of CM-S1PR1 on heart regeneration has not been previously explored. Our study revealed that tamoxifen-inducible CM-specific *S1pr1* deletion significantly reduced cardiomyocyte proliferation and hindered heart regeneration in neonatal mice after heart injuries. Furthermore, CM-specific *S1pr1* overexpression markedly enhanced cardiac regeneration and improved cardiac functions in injured hearts. Our *in vivo* experiments, involving both CM-specific *S1pr1* loss-of-function and gain-of-function, strongly support the crucial role of S1PR1 in regulating cardiac regeneration and promoting cardiac repair and functions after heart injuries.

The recent study by Ji *et al*. demonstrated that sphingolipid metabolism undergoes significant dynamics in neonatal hearts following injury, with SPHK1 and SPHK2, isoenzymes that synthesize sphingosine-1-phosphate (S1P), playing essential roles in regulating cardiac regeneration 33. Consistent with these findings 33, our study also observed dynamic regulation of S1P metabolism during neonatal heart regeneration, with S1P levels increasing post-injury. Ji *et al*. further elucidated that the pro-proliferative effects of S1P depend on the enzymatic activity of sphingosine kinases and the generation of S1P, primarily mediated through epigenetic regulation of genes including *Erbb4*, *Mef2a*, and *Mef2c* 33. Notably, Ji *et al*. highlighted that S1PR1 is the predominant S1P receptor subtype expressed in cardiomyocytes 33. In consistence with this 33, our findings demonstrate that *S1pr1* expression is significantly upregulated in neonatal cardiomyocytes following apical resection (AR) compared to sham controls. These data suggest that the S1P/S1PR1 axis may contribute to the pro-regenerative effects of S1P. Further *in vivo* and *in vitro* analyses in our study confirmed that cardiomyocyte-expressing S1PR1 is essential for cardiomyocyte proliferation and cardiac regeneration. By building on the findings of Ji *et al*. 33, our study provides additional evidence that the S1P/S1PR1 axis is a critical downstream effector in the S1P-mediated regulation of cardiomyocyte proliferation and heart regeneration. These results contribute to a more comprehensive understanding of the molecular mechanisms underlying the role of S1P for cardiac repair and regeneration.

The arrest of cardiomyocyte proliferation in postnatal mice is tightly controlled by cell cycle factors 66. During the arrest of cardiomyocyte cell cycle, negative regulators such as p21, p27, retinoblastoma protein (Rb), and cyclin-dependent kinase inhibitors (CKIs) are up-regulated, while cell cycle activators, including cyclins, cyclin-dependent kinases (CDKs), c-Myc oncogene, and E2F transcription factors, are down-regulated 67, 68. The regulation of cardiomyocyte proliferation mainly occurs during the G1 phase of the cell cycle. The G1 restriction point is triggered by CYCLIN D and CYCLIN E complexes, initiating the entry into the S phase of cell cycle 69. Overexpression of CYCLIN D1 in cardiomyocytes leads to the increased DNA synthesis 70. In consistence with previous studies 70, our data demonstrated that CM-S1PR1 signaling up-regulated CYCLIN D1 expression, thereby enhancing cardiomyocyte proliferation during cardiac regeneration. The PI-3-Kinase (PI3K)/AKT pathway plays a pivotal role in promoting the growth and survival of various cells, including cardiomyocytes. AKT exerts its effects by inactivating pro-apoptotic proteins, activating CYCLIN D1 and CDK2, inhibiting p27, and regulating cell growth through mTOR activity 35. In line with these reports, our data suggested that CM-S1PR1 was involved in the regulation of AKT/mTORC1 activation, which reduced cardiomyocyte apoptosis and upregulated CYCLIN D1 expression, thereby enhancing cardiomyocyte proliferation and promoting cardiac regeneration. Previous studies have demonstrated that mTOR activation is essential for zebrafish cardiomyocyte regeneration 40, and mouse cardiomyocyte survival and heart development 41. A recent elegant study identified mTORC1 as a key regulator of the metabolic switch during postnatal heart development and regeneration 39. Consistent with these findings, our results show that inhibition of mTORC1 significantly blocks S1PR1-induced enhancement of cardiac regeneration, further confirming the crucial role of mTORC1 in cardiac regeneration.

Previous reports and our study suggest that *S1pr1* gene delivery might be a promising therapy for heart failure. Cannavo *et al*. reported *in vivo* gene delivery of *S1pr1* via AAV6 to systemically overexpress *S1pr1* significantly improved cardiac functions in a rat post-MI heart failure model 16. However, systemic overexpression or stimulation of* S1pr1* may cause potential side effects and toxicity, including lethal arrhythmias and severe immunosuppression 71, 72. To mitigate these off-target adverse effects, we constructed AAV9 to express target genes under CM-specific promoter, *cTNT,* achieving high efficiency of CM-specific *S1pr1* overexpression *in vivo*. This CM-specific overexpression of *S1pr1* significantly promoted cardiomyocyte proliferation and improved cardiac functions after myocardial infarction in adult mice, offering a promising cell-specific gene delivery system for treating heart failure. Our studies complement previous findings on the roles of CM-S1PR1 for cardiac healing by providing experimental evidences that CM-targeted overexpression of S1PR1 significantly boosts cardiac regeneration in the injured hearts.

**Figure 10:**
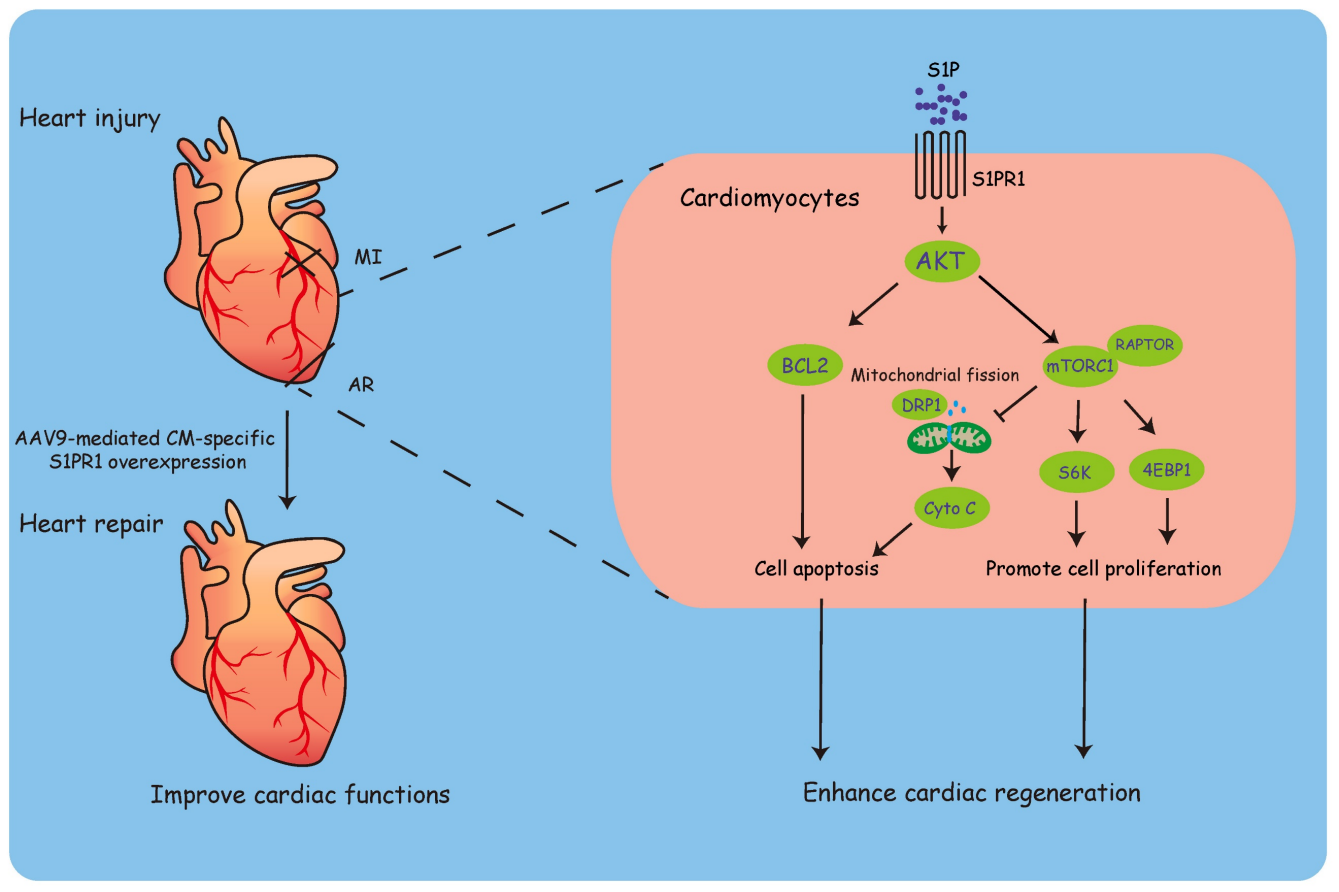
** A work model for cardiomyocyte S1PR1 promotes cardiomyocyte proliferation and cardiac regeneration.** S1PR1 plays a key regulator to regulate cardiomyocyte proliferation during the process of cardiac regeneration. Mechanistically, S1P-S1PR1 signaling promoted cardiomyocyte proliferation and inhibits CM apoptosis via AKT/mTORC1/CYCLIN D1 and BCL2 pathway, respectively. CM-S1PR1 overexpression promotes cardiac regeneration and improves cardiac functions of injured hearts, providing a future promising CM-target therapy for myocardial infarction and heart failure through the S1PR1 signal pathway.

In summary, our investigations identified CM-S1PR1 as a key regulator of cardiomyocyte proliferation during heart regeneration. CM-specific* S1pr1* loss-of-function significantly reduced cardiomyocyte proliferation and impaired cardiac regeneration after heart injuries, while CM-targeted *S1pr1* overexpression enhanced cardiac regeneration and improved cardiac function in injured hearts. Mechanistically, S1P-S1PR1 signaling promoted cardiomyocyte proliferation and inhibited apoptosis via the AKT/mTORC1/CYCLIN D1 and BCL2 pathways. Moreover, CM-targeted gene delivery of *S1pr1* to achieve CM-specific S1PR1 overexpression *in vivo* significantly enhanced cardiomyocyte proliferation and improved cardiac function following myocardial infarction in adult mice (**Figure 10**). This study suggests a promising CM-targeted therapy for myocardial infarction and heart failure through the S1PR1 signal pathway.

## Supplementary Material

Supplementary figures and tables.

## Abbreviations

S1PR1: sphingosine 1-phosphate receptor 1
HF: heart failure
CHD: coronary heart diseases
NMCMs: neonatal mouse cardiomyocytes
ECs: endothelial cells
CFs: cardiac fibroblasts
CMs: cardiomyocytes
AR: apical resection
MI: myocardial infarction
PH3: phospho-histone H3
EdU: 5-ethynyl-2'-deoxyuridine
