# Supplementary material for: Cardiomyocyte S1PR1 promotes cardiac regeneration via AKT/mTORC1 signaling pathway
Source: Theranostics. 2025 Jan 2;15(4):1524–51. doi: 10.7150/thno.103797 (PMC11729560; doi:10.7150/thno.103797)
Supplement: Supplementary file 1 — Supplementary figures and tables. [file thnov15p1524s1.pdf]

Supplementary Figure 1

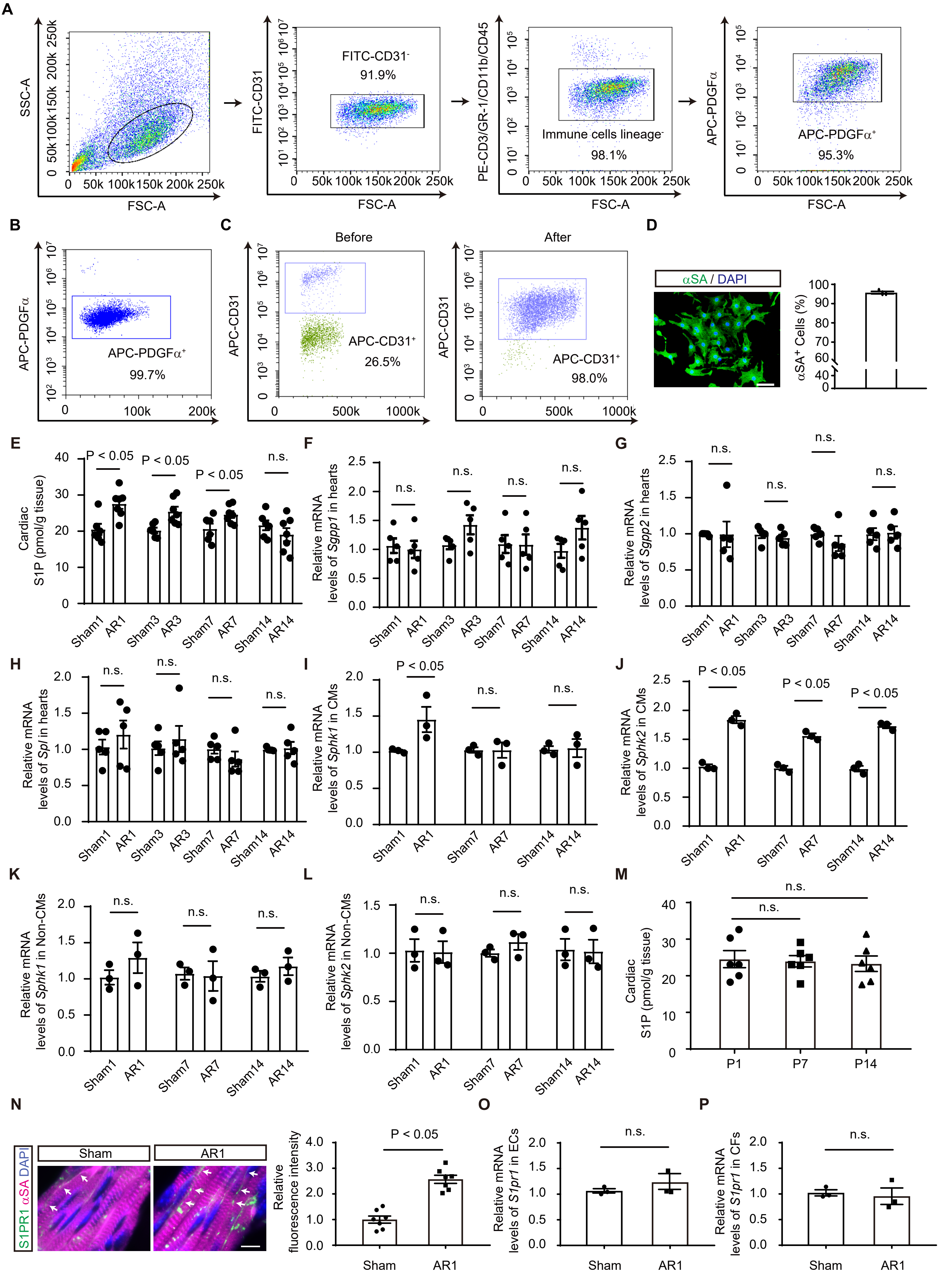

Supplementary Figure 1 (Continued)

Supplementary Figure 1. S1P receptor expression profile in cardiac tissues change after AR injury. A. The representative flow cytometric plots for a typical $CD31^{-}$ $lineage^{-}$ $PDGF\alpha^{+}$ cell sorting procedure for isolation of cardiac fibroblasts from neonatal hearts. **B**. Cytometric analysis of the purity of cardiac fibroblasts after cell sorting, as shown in (**A**). **C**. Cytometric analysis of the purity of cardiac ECs before and after cell purification by CD31-conjugated magnetic beads. **D.** Representative immunostaining images of α-SA with quantification of the purity of cardiomyocytes (n = 3). **E.** Cardiac sphingosine-1-phosphate (S1P) levels in heart tissues collected from mice which underwent the sham operation or the AR operation at postnatal day 3 (P3). Samples were collected at various post-operation time points (1-day, 3-day, 7-day and 14-day) and determined by ELISA (n = 7-8). **F-H.** Relative mRNA expression levels were determined by quantitative RT-qPCR in heart tissues of mice which underwent sham operation at postnatal day 3 (P3). Samples were collected at various post-operation time points (1-day, 3-day, 7-day and 14-day), including *Sgpp1* (**F**), *Sgpp2* (**G**), *Spl* (**H**) (n = 5). **I-J.** Relative mRNA expression levels were determined by quantitative RT-qPCR in cardiomyocytes (CMs) isolated from mice which underwent the sham operations or the AR operation at postnatal day 3 (P3). Samples were collected at various post-operation time points (1-day, 7-day and 14-day) including *Sphk1* (**I**), *Sphk2* (**J**) (n = 3). **K-L.** Relative mRNA expression levels, including *Sphk1* (**K**) and *Sphk2* (**L**), were determined by quantitative RT-qPCR in non-cardiomyocytes of hearts collected from mice which underwent the sham operation or the AR operation at postnatal day 3 (P3). Samples were collected at various post-operation time points (1-day, 7-day and 14-day) (n = 3). **M.** Cardiac sphingosine-1-phosphate (S1P) levels in heart tissues collected from postnatal day 1 (P1), postnatal day 7 (P7) and postnatal day 14 (P14) mice were determined by ELISA (n = 6). **N.** Representative immunostaining images and quantification of percentage of S1PR1 fluorescence intensity in heart tissues from mice which underwent the sham operation or the AR operation at postnatal day 3 (P3). Hearts sections were collected from these mice at 1-day post AR. The arrows indicate that α-SA (magenta) positive cardiomyocytes express S1PR1 (green). DAPI, nuclear staining (blue). Quantification of percentage of S1PR1 fluorescence intensity in immunostaining images of the sham hearts and the AR hearts at 1 day after operation (n= 7). **O**-**P**. Relative mRNA expression levels of S1PR1 in endothelial cells (ECs) (**O**) and cardiac fibroblasts (CFs) (**P**) from the sham hearts or the AR hearts at 1 day after operation in neonatal mice which underwent sham or AR operation at postnatal day 3 (P3)were determined by quantitative RT-qPCR (n = 3). Sham1, 1-day post sham operation; Sham3, 3-day post sham operation; Sham7, 7-day post sham operation; Sham14, 14-day post sham operation; AR1, 1-day post AR; AR3, 3-day post AR; AR7, 7-day post AR; AR14, 14-day post AR; P1, postnatal day 1 (P1); P7, postnatal day 7 (P7); P14, postnatal day 14 (P14). α-SA, α-sacromeric actinin. Data are represented as means ± S.E.M. $P < 0.05$ indicates significant statistical differences. n.s., no statistical significan -ce. Unpaired Student's t-test (E-N). Scale bars: D, 50 μm. N, 15 μm.

Supplementary Figure 2

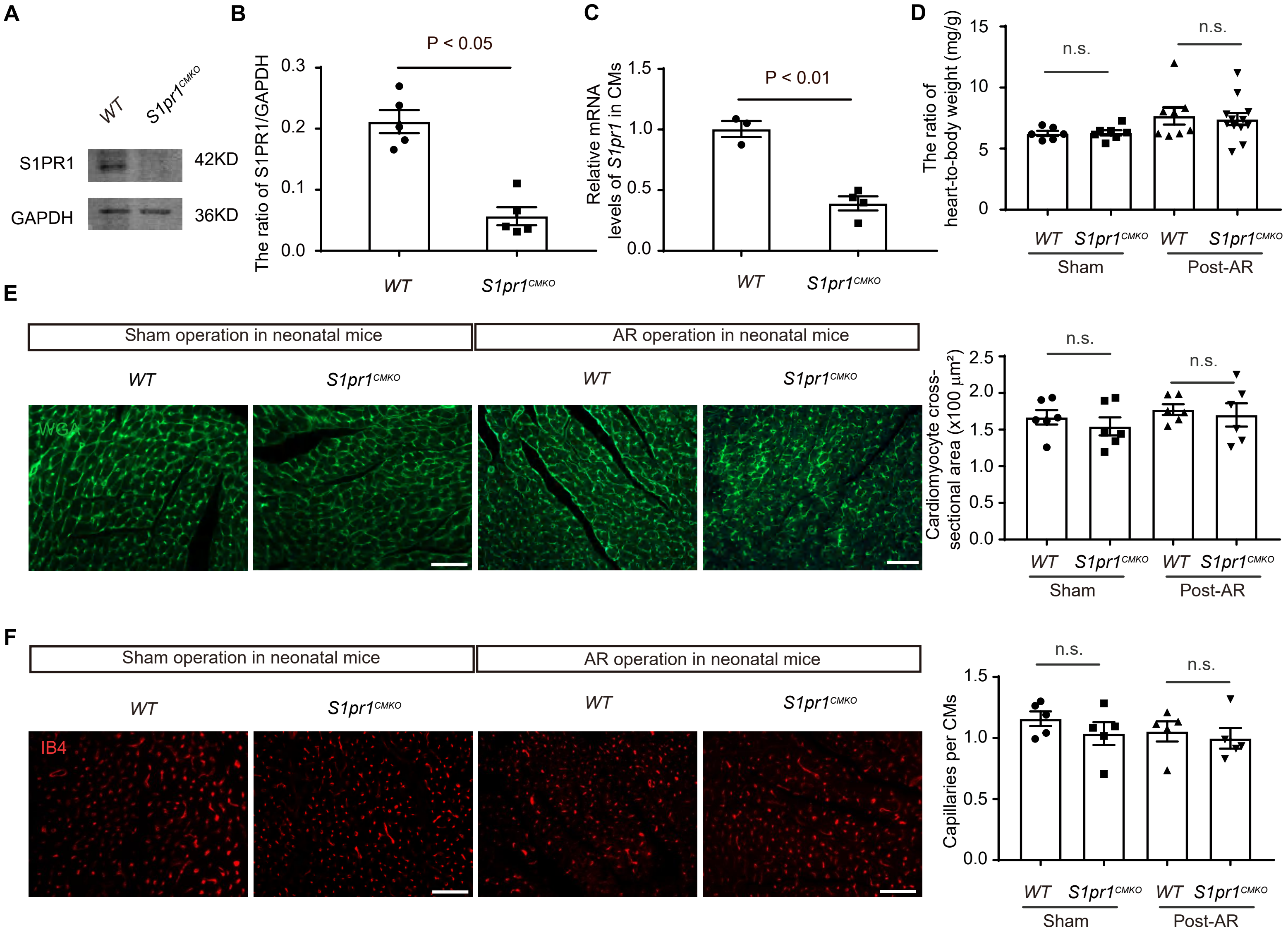

Supplementary Figure 2. The loss of cardiomyocyte-expressing S1PR1 didn't influence cardiac hypertrophy and vascular density after AR in neonatal mice. A-B. Western blot analysis of S1PR1 protein levels in cardiomyocytes from *WT* and $S1pr1^{CMKO}$ neonatal mice after tamoxifen were administered at postnatal day 0 to 1 to induce specific deletion of *S1pr1* in CMs (**A**). Cardiomyocytes (CMs) were collected from these mice at 7-day post tamoxifen injection. The quantification of the ratio of S1PR1/GAPDH was shown in **B** (n = 5). **C.** The relative mRNA expression levels of *S1pr1* in cardiomyocytes was determined by RT-qPCR in the indicated mice after tamoxifen were administered at postnatal day 0 to 1 to induce specific deletion of S1pr1 in CMs (n = 3-4). Cardiomyocytes (CMs) were collected from these mice at 7-day post tamoxifen injection. **D.** The ratio of heart weight to body weight of *WT* and $S1pr1^{CMKO}$ mice which underwent the AR operation or sham operation at postnatal day 3 (P3), and hearts were collected from these mice at 21-day post AR (n = 6-12). **E.** Representative images of WGA (green) staining of hearts from *WT* and $S1pr1^{CMKO}$ mice which underwent the AR operation or sham operation at postnatal day 3 (P3). Hearts sections were collected from these mice at 21-day post AR and quantification of cross-sectional size of CMs on the right (n = 6). **F.** Representative images of IB4 (red) staining of hearts from *WT* and $S1pr1^{CMKO}$ mice which underwent the AR operation or sham operation at postnatal day 3 (P3). Hearts sections were collected from these mice at 21-day post AR and quantification of capillary density on the right (n = 5). WGA, wheat germ agglutinin. IB4, biotinylated-isolectin B4. Data are represented as means ± S.E.M. $P < 0.05$ indicates significant statistical differences. n.s., no statistical significance. Unpaired Student's t-test (**B-F**). Scale bars: **E-F**, 50 μm.

Supplementary Figure 3

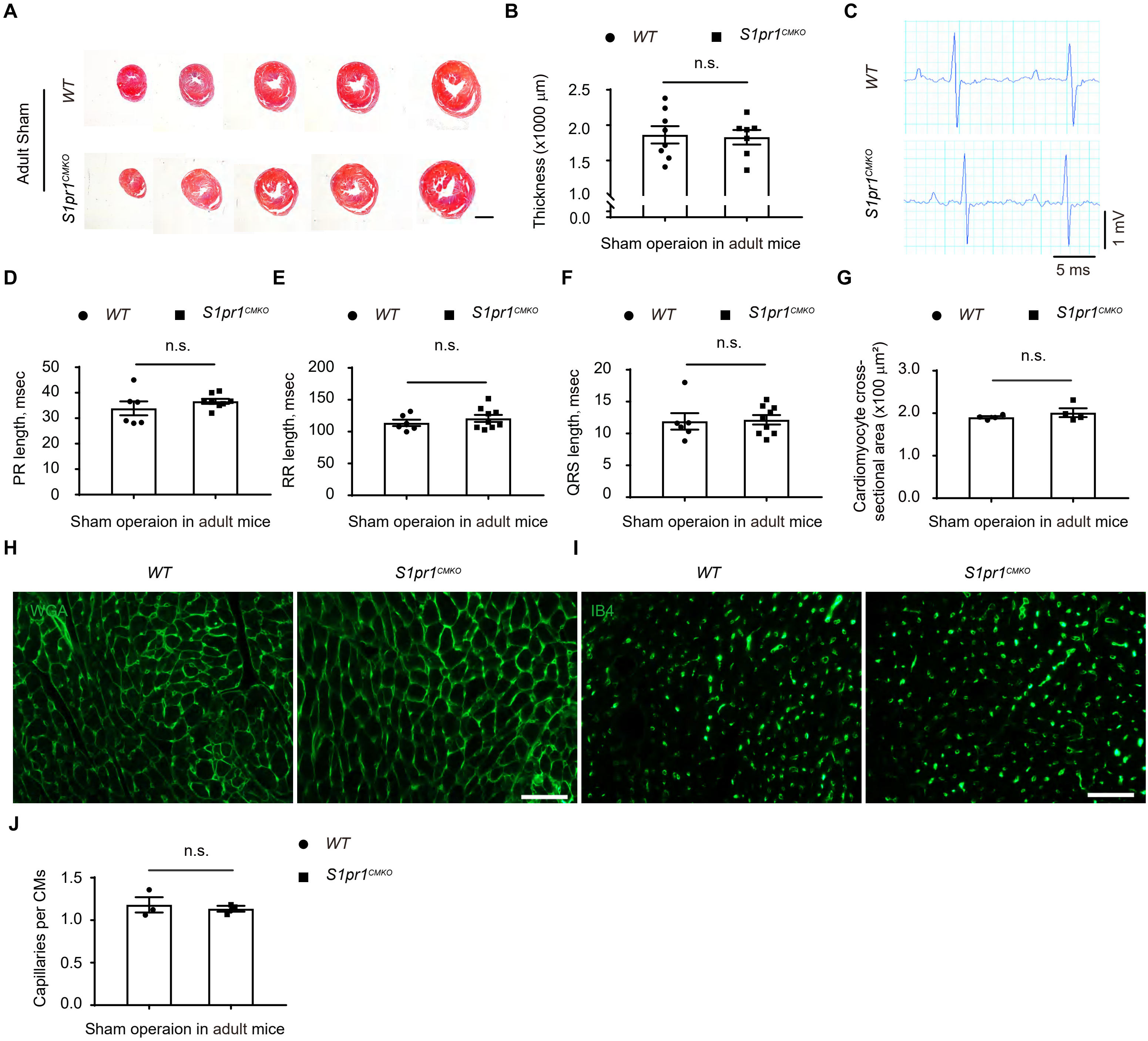

Supplementary Figure 3. Cardiomyocyte S1PR1 deletion didn't affect heart morphology and heart rhythm. A. Representative images of Masson's Trichrome staining of adult sham hearts of *WT* and *S1pr1*^{CMKO} mice (n = 6). **B.** Quantification of the thickness of compact myocardium (CM) of sham hearts from *WT* and *S1pr1*^{CMKO} adult mice (n = 7-8). **C-F.** Representative images of electrocardiogram (**C**) and quantification of the length of PR interval (**D**), RR interval (**E**), and QRS interval (**F**) of sham hearts from *WT* and *S1pr1*^{CMKO} adult mice (n = 6-9). **G-H.** Representative images of WGA (green) staining of sham hearts from *WT* and *S1pr1*^{CMKO} adult mice and quantification of cross-sectional size of CMs (n = 4). **I-J.** Representative images of IB4 (green) staining of sham hearts of *WT* and *S1pr1*^{CMKO} adult mice (**I**) and quantification of capillary density (**J**) (n = 3). WGA, wheat germ agglutinin. IB4, biotinylated-isolectin B4. Data are represented as means ± S.E.M. $P < 0.05$ indicates significant statistical differences. n.s., no statistical significance. Unpaired Student's t-test (**B**, **D-G**, and **J**). Scale bars: **A**, 2 mm, **H**, **I**, 50 μm.

Supplementary Figure 4

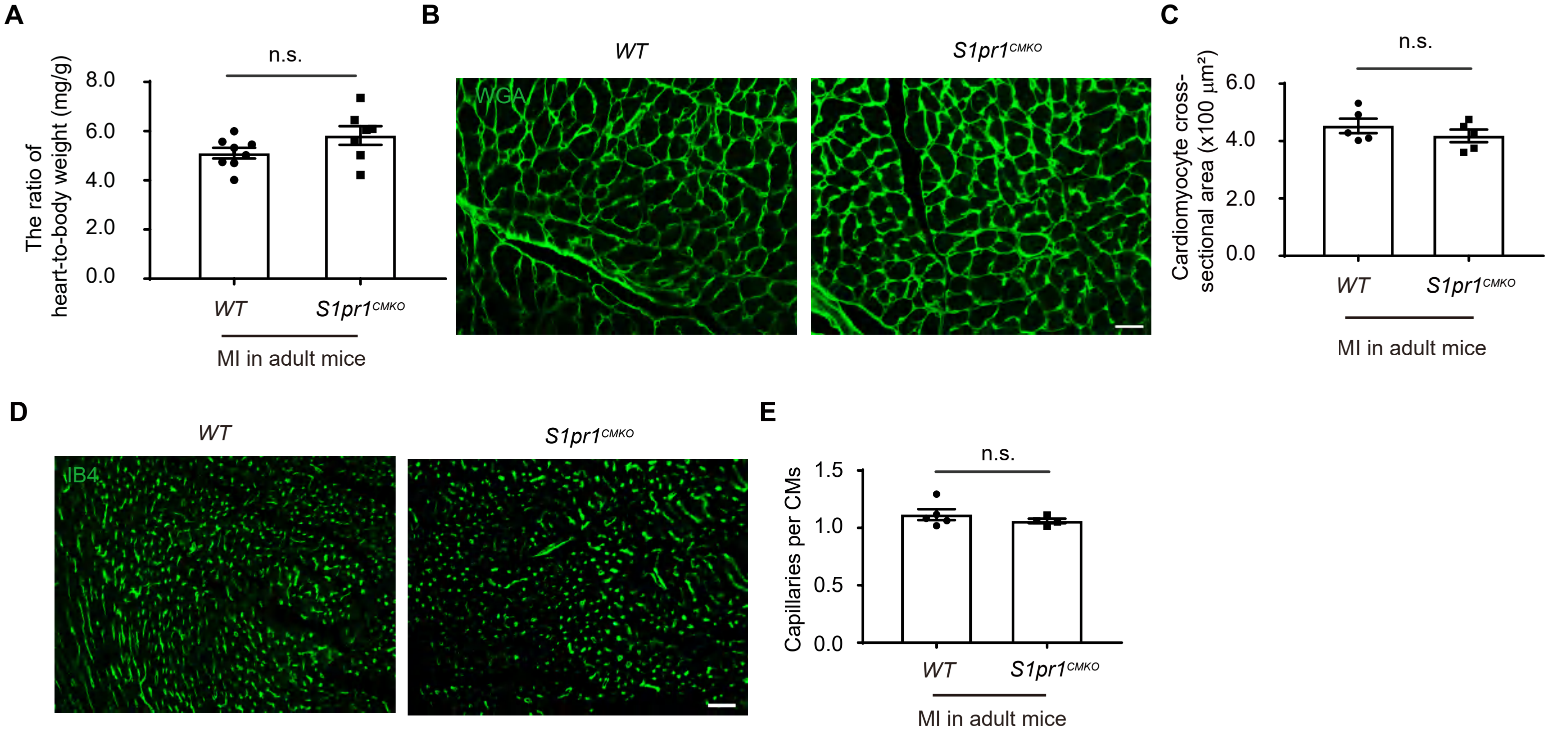

Supplementary Figure 4. Cardiomyocyte S1PR1 deletion didn't influence cardiac hypertrophy and vascular density after adult myocardial infarction. A. The ratio of heart weight to body weight of *WT* and *S1pr1*^{CMKO} adult mice which underwent the MI operation at age of 8 weeks, and hearts were collected from these mice at 28-day post MI (n = 7-8). **B-C.** Representative images of WGA (green) staining of *WT* and *S1pr1*^{CMKO} adult mice which underwent the MI operation at age of 8 weeks. Hearts sections were collected from these mice at 28-day post MI (**B**) and quantification of cross-sectional size of CMs (**C**) (n = 5). **D-E.** Representative images of IB4 (green) staining of *WT* and *S1pr1*^{CMKO} adult mice which underwent the MI operation at age of 8 weeks. Hearts sections were collected from these mice at 28-day post MI (**D**), and quantification of capillary density (**E**) (n = 5). WGA, wheat germ agglutinin. IB4, biotinylated-isolectin B4. Data are represented as means ± S.E.M. P < 0.05 indicates significant statistical differences. n.s. indicates no statistical significance. Unpaired Student's t-test (**A**, **C** and **E**). Scale bars: **B**, **D**, 50 μm.

Supplementary Figure 5

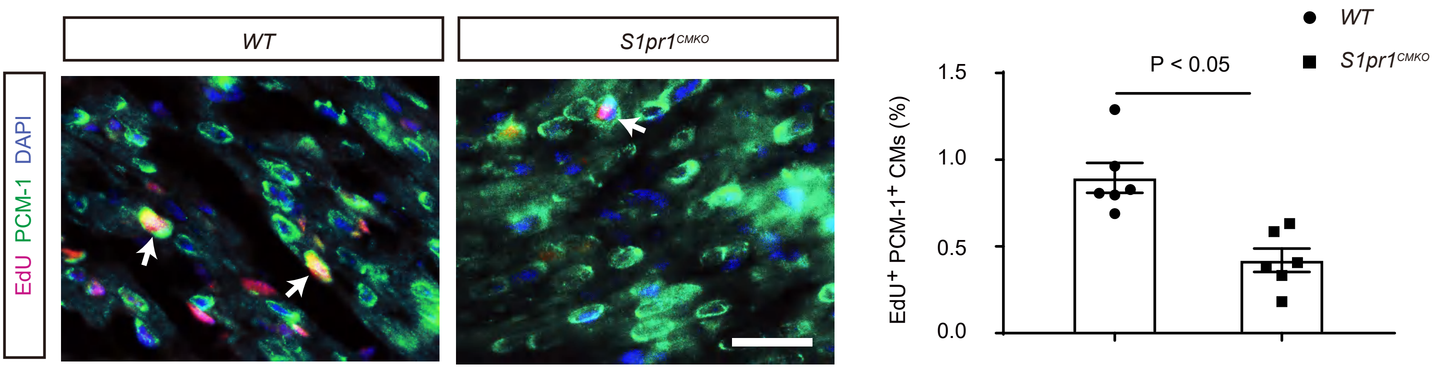

Supplementary Figure 5. The cardiomyocyte-specific loss of S1PR1 inhibits cardiac proliferation after MI in adult mice. Representative immunostaining images on peri-infarct sections for EdU and PCM1 positive cardiomyocytes of injured hearts from *WT* and *S1pr1*^{CMKO} mice which underwent the MI operation at age of 8 weeks. Hearts sections were collected from these mice at 7-day post MI. The arrows indicate PCM1 (green) cardiomyocytes positive for EdU (magenta). DAPI, nuclear staining (blue). Quantification of the percentage of EdU^{+}PCM1^{+} on the right (n = 6). PCM1, Pericentriolar Material 1. EdU, 5-ethynyl-2'-deoxyuridine. Data are represented as means ± S.E.M. $P < 0.05$ indicates significant statistical differences. Unpaired Student's t-test. Scale bars: 25 μm.

Supplementary Figure 6

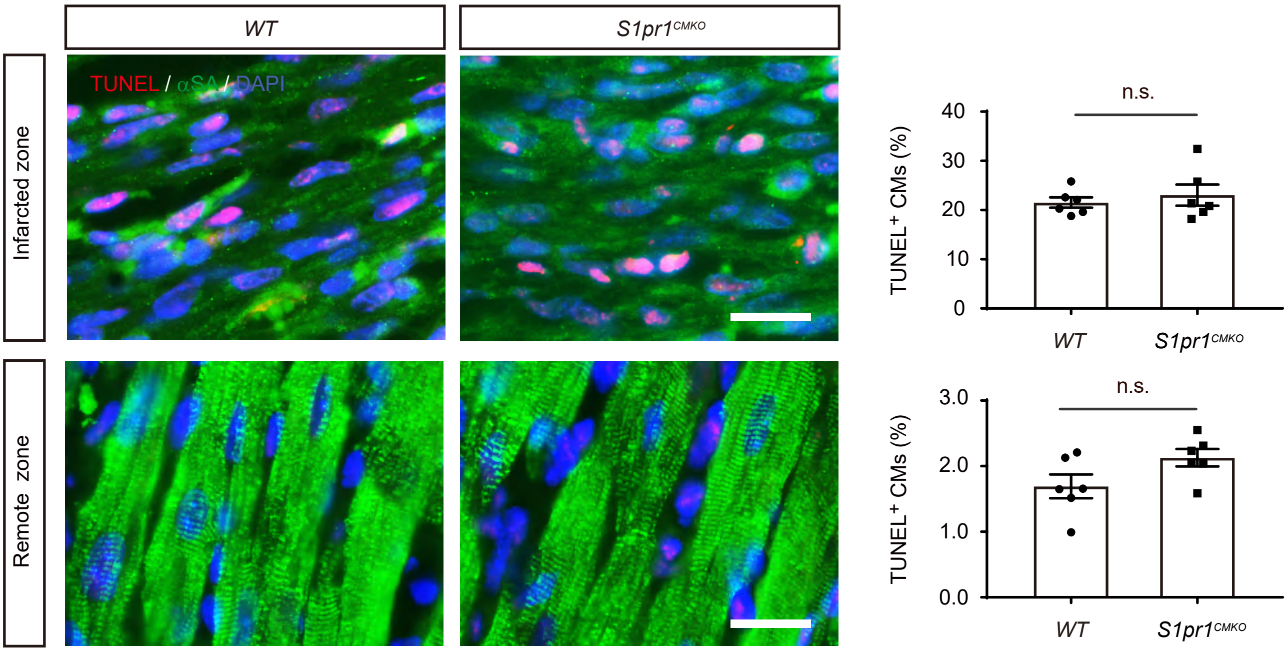

Supplementary Figure 6. The cardiomyocyte-specific loss of S1PR1 didn't influence cardiac apoptosis in infarcted zone or remote zone of heart from adult mice post-MI. Representative immunostaining images on sections for TUNEL and α-SA positive cardiomyocytes of infarcted zone or remote zone of injured hearts from *WT* and *S1pr1^{CMKO}* mice which underwent the MI operation at age of 8 weeks. Hearts sections were collected from these mice at 7-day post MI (n = 6). The arrows indicate α-SA (green) cardiomyocytes positive for TUNEL (magenta). DAPI, nuclear staining (blue). Quantification of the percentage of TUNEL$^+$α-SA$^+$ cardiomyocytes on the right. α-SA, α-sacromeric actinin. Data are represented as means ± S.E.M. $P < 0.05$ indicates significant statistical differences. n.s., no statistical significance. Unpaired Student's t-test. Scale bars: 25 μm.

Supplementary Figure 7

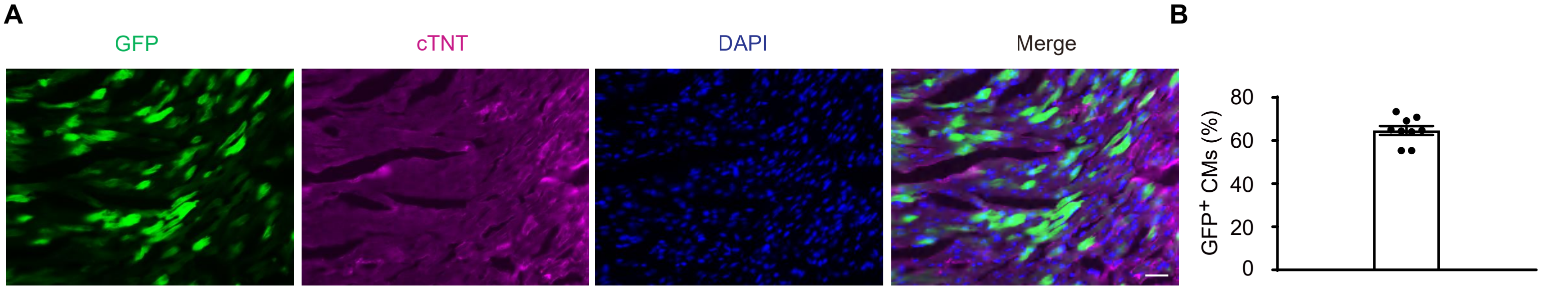

Supplementary Figure 7. AAV9-*cTnT-GFP* achieved a high efficiency of CM-specific express target genes *in vivo*. A-B. The representative images of GFP positive cells (green) and cTNT (magenta) positive cardiomyocytes of mice which underwent the *AAV9-cTNT-GFP* virus administration at postnatal day 1 (P1) (**A**). Hearts sections were collected from these mice at 7-day post-infection. The quantification of GFP^+ $cTNT^+$ cells (%) as the efficiency of AAV infection was shown in **B** (n = 9). DAPI, nuclear staining (blue). cTNT, cardiac troponin T. Data are represented as means ± S.E.M. Scale bar: **A**, 50 μm.

Supplementary Figure 8

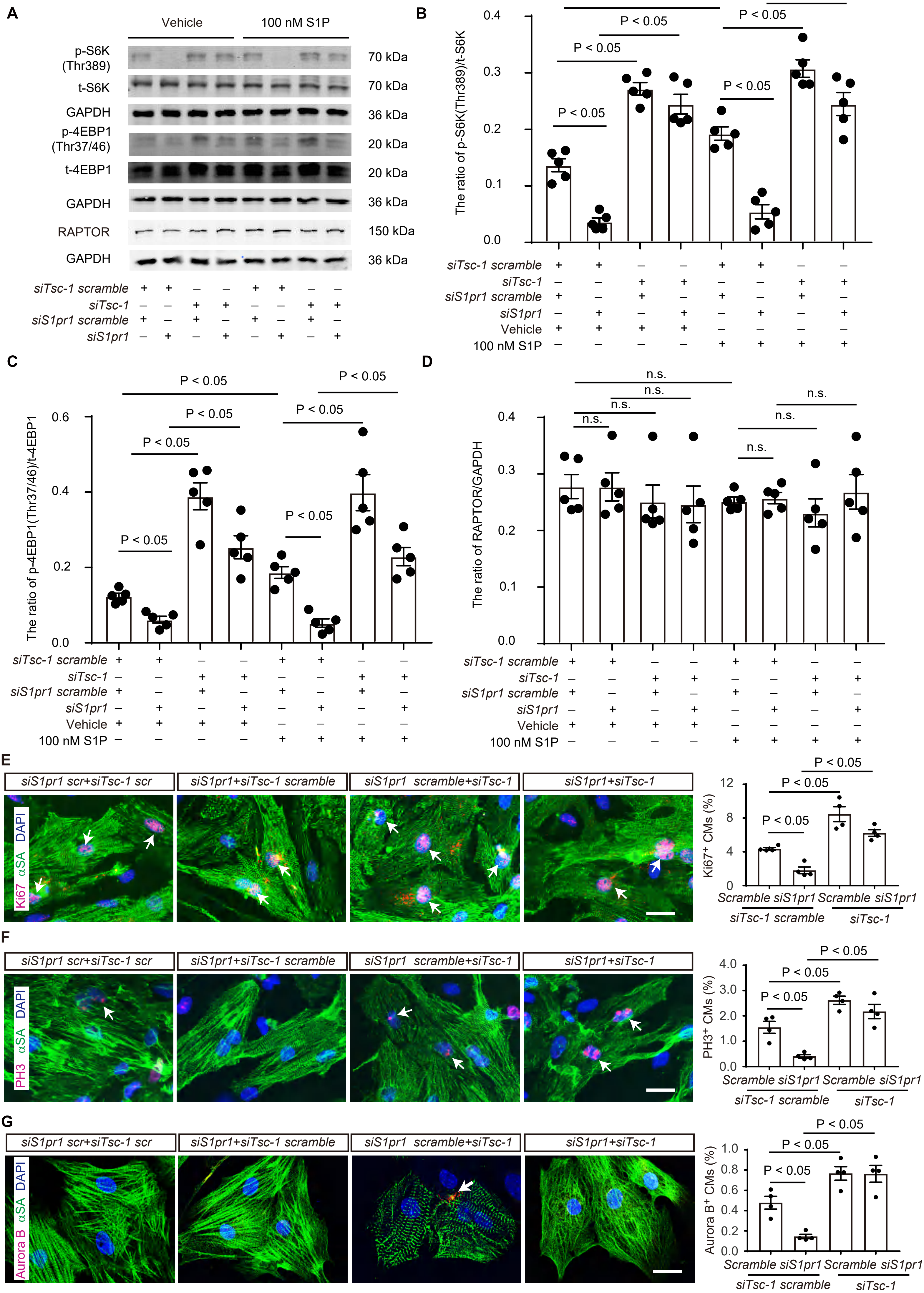

Supplemetary Figure 8 (Continued)

Supplementary Figure 8. S1PR1 knockdown decreases cardiomyocyte proliferation via AKT/mTORC1 signaling pathways. A-D. Western blot analysis of total and phosphorylated S6K (Thr389), total and phosphorylated 4EBP1 (Thr37/46) and RAPTOR protein levels in neonatal mouse cardiomyocytes (NMCMs) which were starved overnight and treated with or without *S1pr1-siRNA* or *Tsc1-siRNA* (**A**) before stimulation with vehicle or 100 nM S1P. The activity and expression levels were shown by quantification of the ratios of phosphorylated S6K (Thr389) to total S6K, p-S6K(Thr389)/t-S6K (**B**), the ratios of phosphorylated 4EBP1 (Thr37/46) to total 4EBP1, p-4EBP1(Thr37/46)/t-4EBP1 (**C**) and the ratios of RAPTOR to GAPDH (RAPTOR/GAPDH) (**D**) (n = 5). **E-G.** Representative immunostaining images of for Ki67 (**E**), PH3 (**F**) or Aurora B (**G**) and α-SA positive neonatal mouse cardiomyocytes (NMCMs) treated with or without *S1pr1-siRNA* and *Tsc1-siRNA*. The arrows indicate α-SA (green) cardiomyocytes positive for Ki67 (magenta), PH3 (magenta) or Aurora B (magenta). DAPI, nuclear staining (blue). Quantification of the percentage of $Ki67^{+}\alpha\text{-}SA^{+}$ (**E**), $PH3^{+}\alpha\text{-}SA^{+}$ (**F**) and Aurora $B^{+}\alpha\text{-}SA^{+}$ (**G**) cardiomyocytes (n = 4). α-SA, α-sacromeric actinin. PH3, phospho-histone H3. *siS1pr1 scr*, *siS1pr1 scramble. siTsc-1 scr, siTsc-1 scramble. siS1pr1*, *S1pr1*-siRNA. *siTsc-1*, *Tsc1*-siRNA. S6K, ribosomal S6 kinase. 4EBP1, eIF4E-binding protein 1. RAPTOR, regulatory-associated protein of mammalian target of rapamycin. Data are represented as means ± S.E.M. $P < 0.05$ indicates significant statistical differences. Scale bars: **E-G**, 15 μm. One-way ANOVA (**B-G**).

Supplementary Figure 9

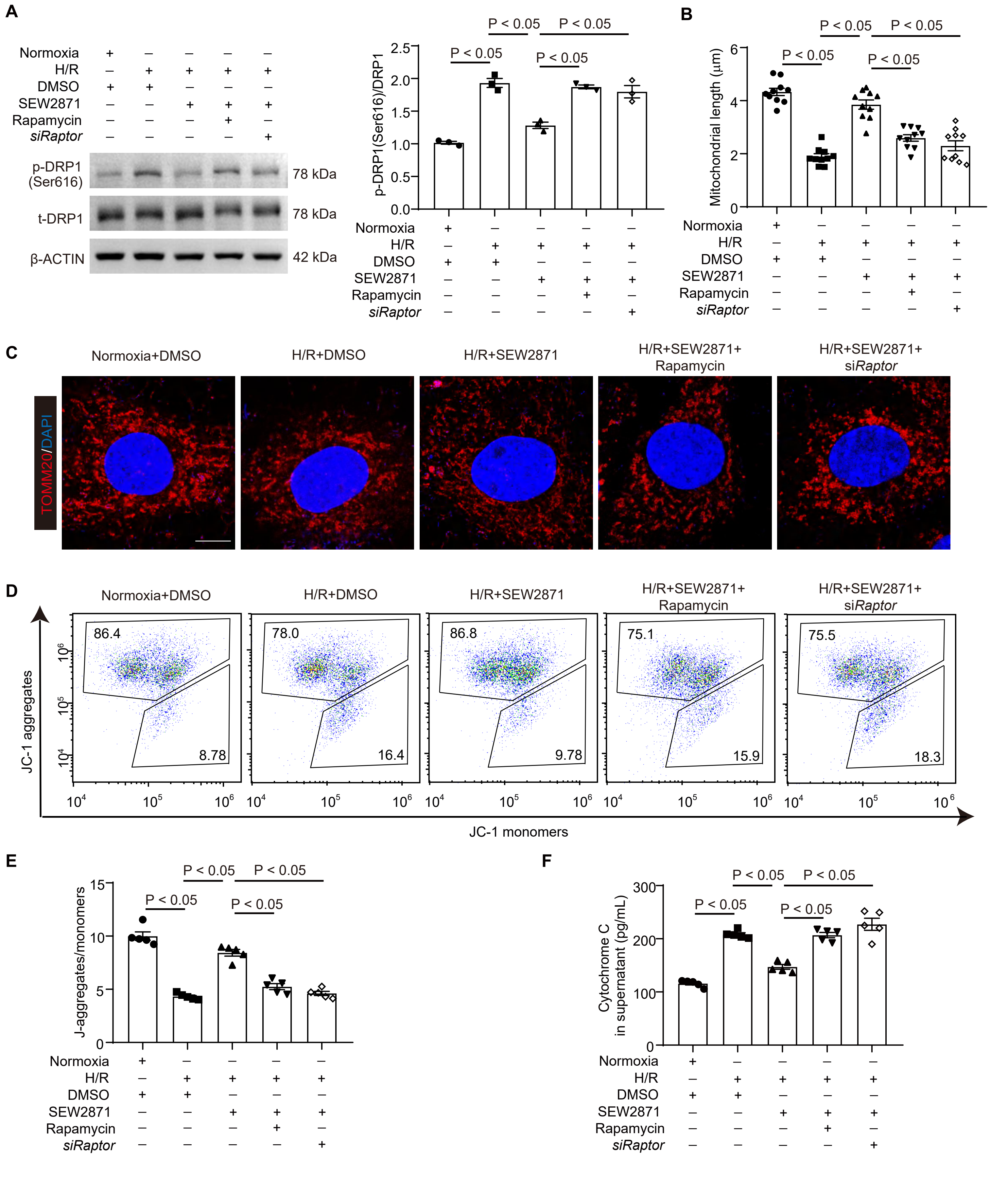

Supplementary Figure 9. S1PR1 attenuates mitochondrial hyperfission to inhibit cardiomyocyte apoptosis by mTORC1 signaling. A. Western blot analysis was conducted to assess the levels of total and phosphorylated DRP1 (Ser616) in MCMs treated with or without SEW2871, Rapamycin or Raptor-siRNA under the normoxia or 24h-hypoxia/12h-reoxygenation condition, with quantification of the ratio of p-DRP1(Ser616)/t-DRP1 (n=3). **B-C.** The representative images of the Tomm20 staining to visualize mitochondrial morphology in mouse cardiomyocytes (MCMs) treated with or without SEW2871, Rapamycin or Raptor-siRNA under the normoxia or 24h-hypoxia/12h-reoxygenation condition (**C**). Quantification of mitochondrial length (n = 10) in the indicated groups(**B**). **D-E**. The representative images of flow cytometric analysis of JC-1 staining in MCMs treated with or without SEW2871, Rapamycin or Raptor-siRNA under the normoxia or 24h-hypoxia/12h-reoxygenation condition for the detection of mitochondrial membrane potential changes (**D**). Quantification of the ratio of J-aggregates to monomer (n = 5) in the indicated groups (**E**). **F**. Cytochrome C in supernatant of MCMs treated with or without SEW2871, Rapamycin or Raptor-siRNA under the normoxia or 24h-hypoxia/12h-reoxygenation condition were determined by ELISA (n = 5). TOMM20, translocase of outer mitochondrial membrane 20. H/R, 24-hour hypoxia/12-hour reoxygenation condition. CYCS, Cytochrome C. SEW2871, S1PR1 agonist. Rapamycin, mTOR1 inhibitor. JC-1, 5,5',6,6'-Tetrachloro-1,1',3,3'-tetraethylbenzimidazolocarbocyanine iodide. DRP1, dynamin-related protein 1. *siRaptor*, *Raptor-siRNA*. Data are represented as means ± S.E.M. P < 0.05 indicates significant statistical differences. Scale bar: **B**, 5 μm. One-way ANOVA (**A-B**, and **E-F**).

Supplementary Figure 10

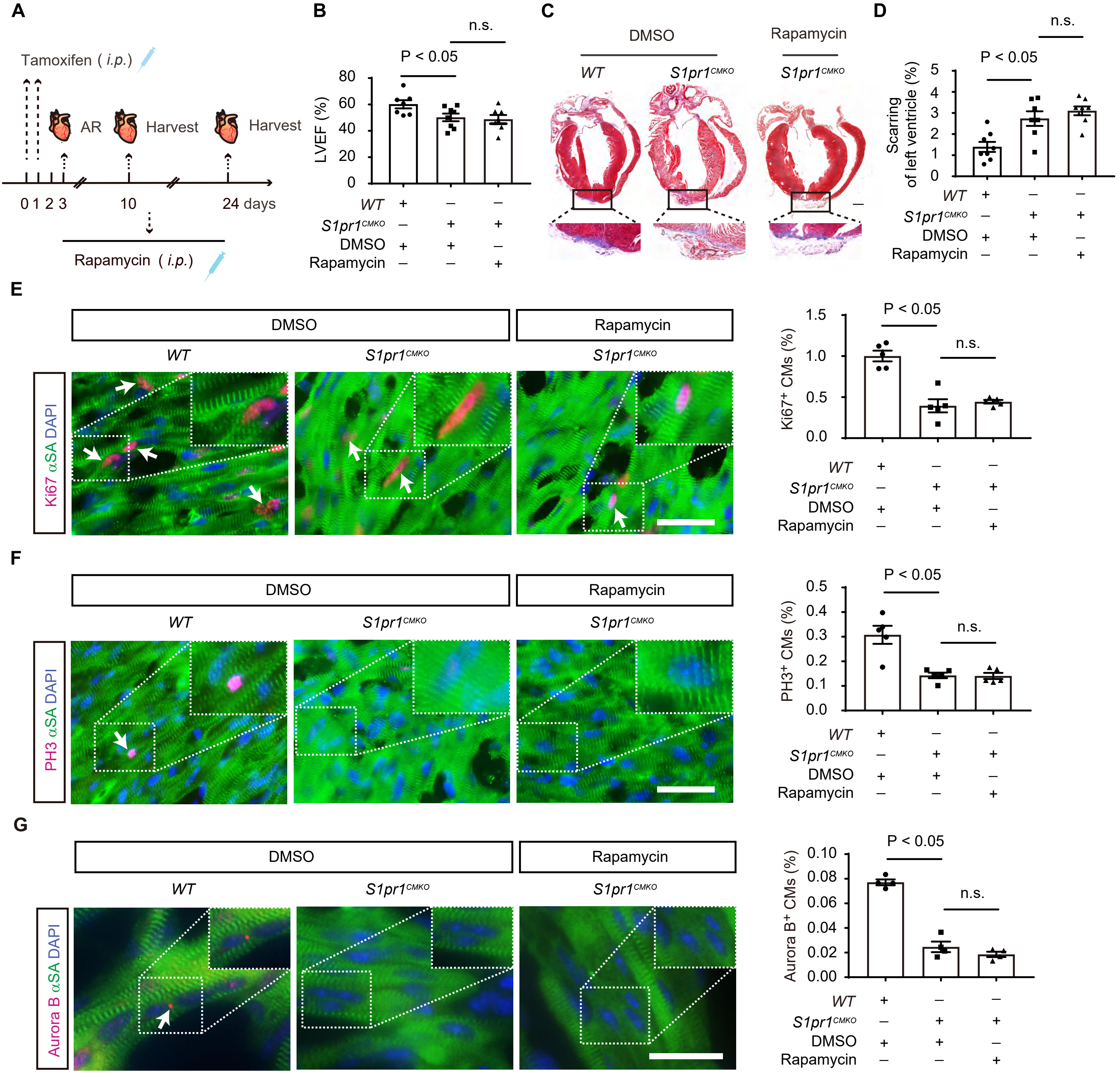

Supplementary Figure 10. The effect of rapamycin on cardiac regeneration in *S1pr1^{CMKO}* neonatal mice after AR. A. Schematic diagram of generation of cardiomyocyte-specific *S1pr1* knockout mice (*S1pr1^{CMKO}*), and tamoxifen (dosed at 40 μg daily) were administered to neonatal mice from postnatal day 0 to 1 to induce specific deletion of *S1pr1* in CMs following with apical resection (AR) at postnatal day 3 (P3). DMSO or rapamycin was administered to *S1pr1^{CMKO}* mice every day (2 mg/kg body weight, *i.p.*) post AR operation. Hearts tissues were harvested from wild-type (*WT*) and *S1pr1^{CMKO}* mice at designated post-AR time points (7-day and 21-day). **B.** Quantitative assessment of left ventricle ejection fraction (LVEF%) in wild-type (*WT*) and *S1pr1^{CMKO}* mice which underwent the AR operation at postnatal day 3 (P3) with or without rapamycin treatment were performed at 21-day post AR using echocardiography (n = 7-8). **C-D.** Representative images of Masson's Trichrome staining in *WT* and *S1pr1^{CMKO}* mice which underwent the AR operation at postnatal day 3 (P3) with or without rapamycin treatment. Hearts sections were collected from these mice at 21-day post AR (**C**), and quantification of the percentage of cardiac scars in left ventricle (n = 7-8) (**D**). **E-F.** Representative immunostaining images on heart sections for Ki67 (**E**) or PH3 (**F**) and α-SA positive cardiomyocytes within the border zone of hearts from *WT* and *S1pr1^{CMKO}* mice which underwent the AR operation at postnatal day 3 (P3) with or without rapamycin treatment. Hearts sections were collected from these mice at 7-day post AR. The arrows indicate α-SA (green) cardiomyocytes positive for Ki67 (magenta) or PH3 (magenta). DAPI, nuclear staining (blue). Quantification of the percentage of Ki67^{+}α-SA^{+} or PH3^{+}α-SA^{+} cardiomyocytes on the right (n = 4-5). α-SA, α-sacromeric actinin. PH3, phospho-histone H3. Data are represented as means ± S.E.M. $P < 0.05$ indicates significant statistical differences. n.s. indicates no statistical significance. One-way ANOVA (**B**, **D**, and **E-G**). Scale bars: **C**, 2 mm; **E-G**, 15 μm.

Supplementary Figure 11

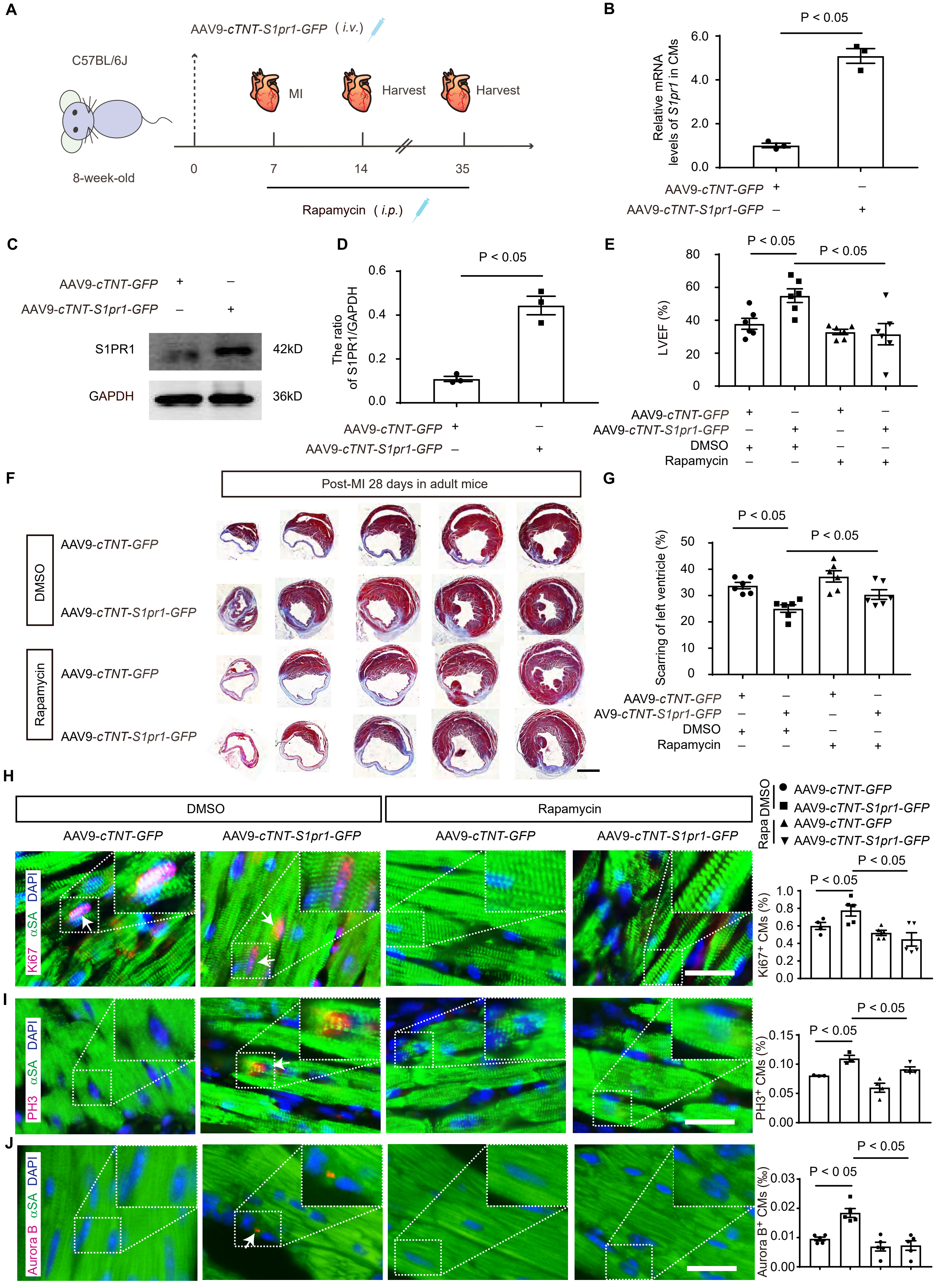

Supplementary Figure 11 (Continued)

Supplementary Figure 11. S1PR1 overexpression increases cardiomyocyte proliferation via mTORC1 signaling pathways in adult mice. A. Schematic diagram for experimental procedure, AAV9-*cTNT-S1pr1-GFP* driven by *cTnT* promoter were administered to 8-week-old mice to achieve cardiomyocyte (CM)-specific S1PR1 overexpression, following by myocardial infarction (MI) operation at 7-day after AAV administration. Rapamycin was administered every day (2 mg/kg bodyweight, *i.p.*) post MI operation and EdU was administered daily for 3 consecutive days beginning from 4-day post-MI. Hearts tissues from sham-operated and MI-operated mice were harvested at designated post-MI time points (7-day, 28-day). **B**. Relative mRNA expression levels of S1pr1 in CMs of the indicated groups from the adult mice infected with AAV9-*cTnT-GFP* or AAV9-*cTNT-S1pr1-GFP* (4 × 10^{11} viral genome particles per mouse, *i.p.*) (n = 3). **C-D**. Western blot analysis of S1PR1 protein levels in CMs from the adult mice infected with AAV9-cTnT-GFP or AAV9-cTnT-S1pr1-GFP (n = 3). **E**. Quantitative assessment of left ventricle ejection fraction (LVEF%) in AAV9-*cTNT-S1pr1-GFP* and AAV9-*cTnT-GFP* adult mice which underwent the MI operation at age of 8 weeks with or without rapamycin treatment were performed at 28-day post MI using echocardiography. (n = 6). **F-G**. Representative images of Masson's Trichrome staining in AAV9-cTnT-S1pr1-GFP and AAV9-cTnT-GFP mice which underwent the MI operation at age of 8 weeks. Hearts sections were collected from these mice at 28-day post MI (**F**), and quantification of the percentage of cardiac scar area in left ventricles on the right (n = 6) (**G**). **H-J**. Representative immunostaining images on peri-infarct sections for Ki67 (**H**), PH3 (**I**) or Aurora B (**J**) and α-SA positive cardiomyocytes of hearts from AAV9-*cTNT-S1pr1-GFP* or AAV9-*cTnT-GFP* mice which underwent the MI operation at age of 8 weeks. Hearts sections were collected from these mice at 7-day post MI. The arrows indicate α-SA (green) cardiomyocytes positive for Ki67 (magenta), PH3 (magenta) or Aurora B (magenta). DAPI, nuclear staining (blue). Quantification of the percentage of $Ki67^{+}\alpha\text{-}SA^{+}$, $PH3^{+}\alpha\text{-}SA^{+}$, $EdU^{+}\alpha\text{-}SA^{+}$, Aurora $B^{+}\alpha\text{-}SA^{+}$ and $TUNEL^{+}\alpha\text{-}SA^{+}$ cardiomyocytes on the right. α-SA, α-sacromeric actinin. PH3, phospho-histone H3. Rapamycin (Rapa), mTOR inhibitor. Data are represented as means ± S.E.M. $P < 0.05$ indicates significant statistical differences. One-way ANOVA (**E** and **G-J**). Unpaired Student's t-test (**B** and **D**). Scale bars: **C**: 2 mm; **H-J**, 25 μm.

Supplementary Figure 12

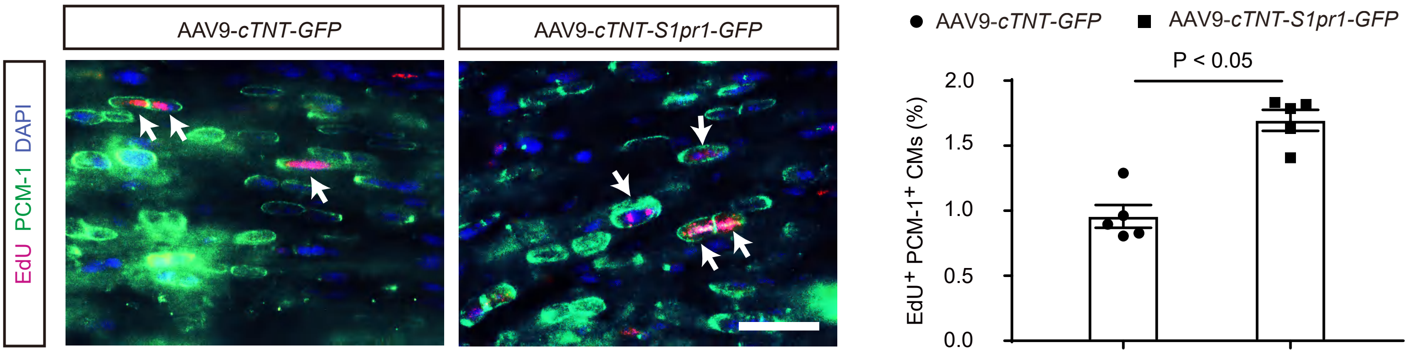

Supplementary Figure 12. Overexpression of S1PR1 in cardiomyocytes promotes cardiac proliferation after MI in adult mice. Representative immunostaining images on peri-infarct sections for EdU and PCM1 positive cardiomyocytes of injured hearts from *AAV9-cTnT-S1pr-GFP* and *AAV9-cTnT-GFP* mice which underwent the MI operation at age of 8 weeks. Hearts sections were collected from these mice at 7-day post MI. The arrows indicate PCM1 (green) cardiomyocytes positive for EdU (magenta). DAPI, nuclear staining (blue). Quantification of the percentage of $EdU^{+}PCM1^{+}$ on the right (n = 5). PCM1, Pericentriolar Material 1. EdU, 5-ethynyl-2'-deoxyuridine. Data are represented as means ± S.E.M. $P < 0.05$ indicates significant statistical differences. Unpaired Student's t-test. Scale bars: 25 μm.

Supplementary Figure 13

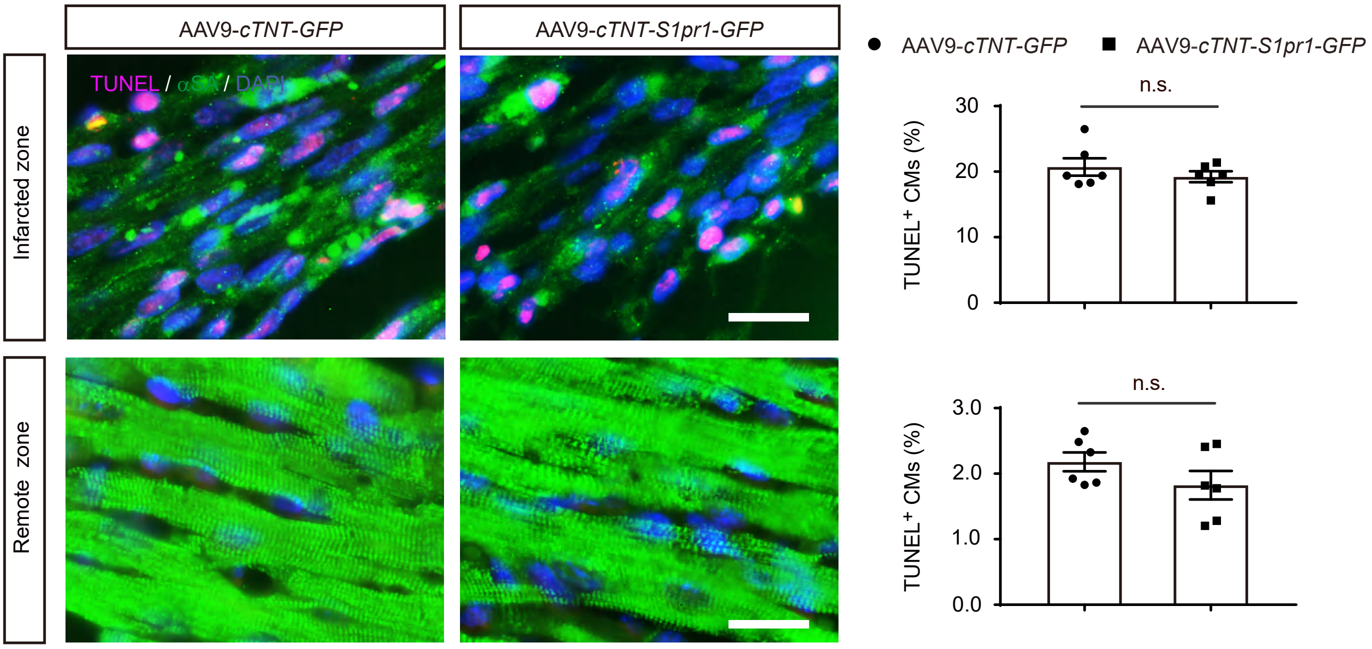

Supplementary Figure 13. Overexpression of S1PR1 in cardiomyocytes didn't influence cardiac apoptosis in infarcted zone or remote zone of heart from adult mice post-MI. Representative immunostaining images on sections for TUNEL and α-SA positive cardiomyocytes of infarcted zone or remote zone of injured hearts from AAV9-*cTnT-S1pr1-GFP* and AAV9-*cTnT-GFP* mice which underwent the MI operation at age of 8 weeks. Hearts sections were collected from these mice at 7-day post MI (n = 6). The arrows indicate α-SA (green) cardiomyocytes positive for TUNEL (magenta). DAPI, nuclear staining (blue). Quantification of the percentage of $TUNEL^{+}\alpha\text{-}SA^{+}$ cardiomyocytes on the right. α-SA, α-sacromeric actinin. Data are represented as means ± S.E.M. $P < 0.05$ indicates significant statistical differences. n.s., no statistical significance. Unpaired Student's t-test. Scale bars: 25 μm.

Supplementary Figure 14

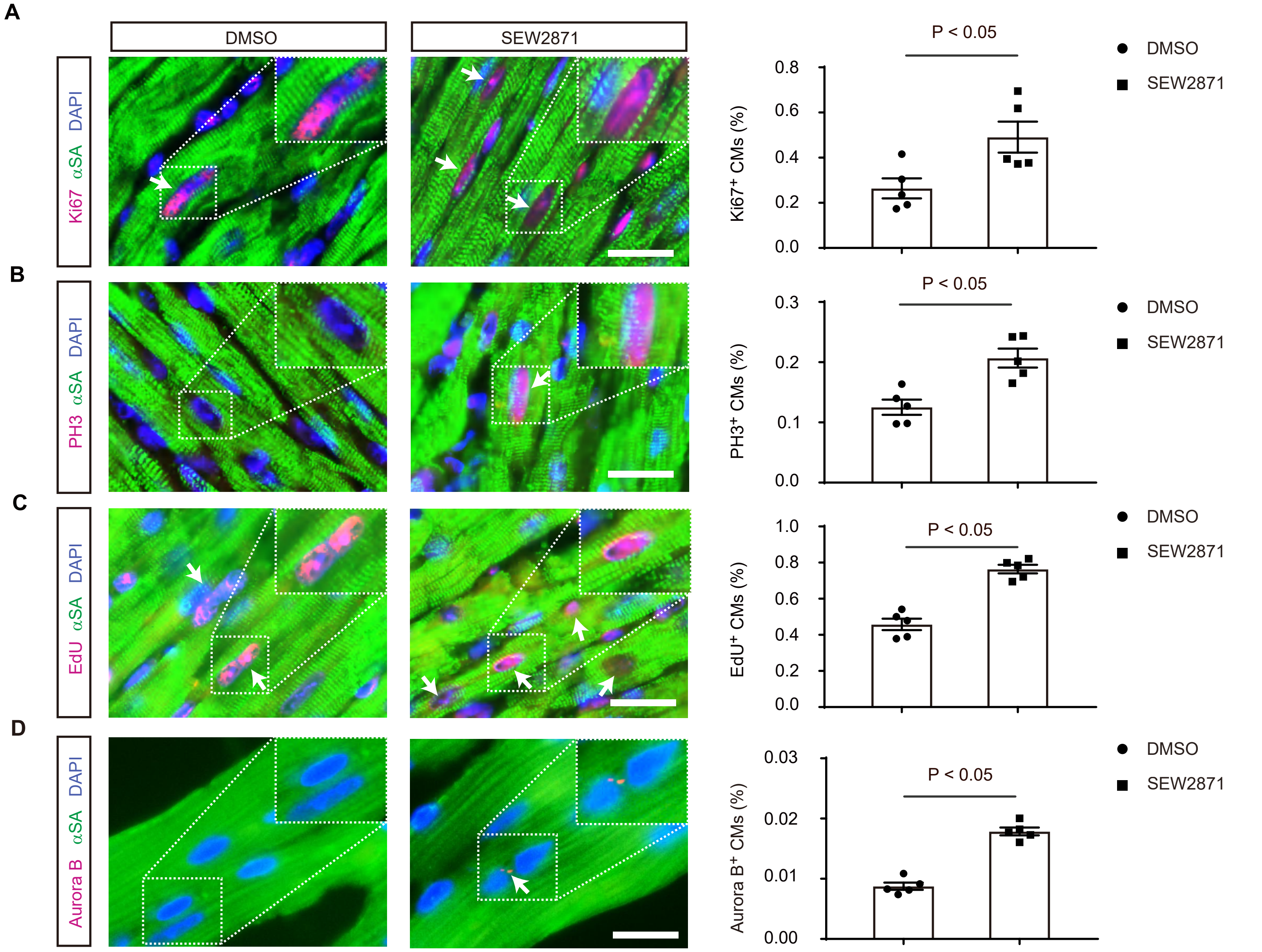

Supplementary Figure 14. SEW2871 promotes cardiac proliferation after MI in adult mice. A-D. Representativeimmunostaining images on peri-infarct sections for Ki67 (**A**), PH3 (**B**), EdU (**C**), Aurora B (**D**) and α-SA positive cardiomyocytes of injured hearts from mice treated with SEW2871 after MI operation at postnatal day 56 (P56). Hearts sections were collected from these mice at 7-day post MI. The arrows indicate α-SA (green) cardiomyocytes positive for Ki67 (magenta), PH3 (magenta), EdU (magenta) or Aurora B (magenta). DAPI, nuclear staining (blue). Quantification of the percentage of $Ki67^{+}\alpha\text{-}SA^{+}$, $PH3^{+}\alpha\text{-}SA^{+}$, $EdU^{+}\alpha\text{-}SA^{+}$ and Aurora $B^{+}\alpha\text{-}SA^{+}$ cardiomyocytes on the right (n = 5). α-SA, α-sacromeric actinin. PH3, phospho-histone H3. EdU, 5-ethynyl-2'-deoxyuridine. Data are represented as means ± S.E.M. P < 0.05 indicates significant statistical differences. Unpaired Student's t-test (**A-D**). Scale bars: **A-D**, 25 μm.

Supplemental Tables

Supplementary Table 1. Antibodies used for western blot and immunostaining.

Antibodies	Company	Catalog Number
Anti-mouse α-sacromeric actinin (αSA)	Abcam	Ab9465
Anti-rabbit Tomm20	Abcam	Ab56783
Anti-rabbit PH3	Abcam	Ab80612
Anti-rabbit Ki67	Abcam	Ab15580
Anti-mouse Ki67	CST	9449S
Anti-rabbit cTNT	Abcam	Ab115134
Anti-rabbit Aurora B	Abcam	Ab315206
Anti-rabbit p-AKT (Ser473)	CST	4060T
Anti-rabbit t-AKT	CST	4060S
Anti-rabbit BCL2	CST	3498T
Anti-rabbit p-DRP1 (Ser616)	CST	4494T
Anti-rabbit DRP1	CST	8570T
Anti-rabbit S1PR1	Invitrogen	PA1-1040
Anti-rabbit S1PR2	Proteintech	21180-1-AP
Anti-rabbit S1PR3	ABclonal	A15664
Anti-rabbit p-S6K (Thr389)	ABclonal	AP0564
Anti-rabbit t-S6K	ABclonal	A2190
Anti-rabbit p-4EBP1 (Thr37/46)	ABclonal	AP1363
Anti-rabbit t-4EBP1	ABclonal	A24691
Anti-rabbit RAPTOR	ABclonal	A21755
Anti-rabbit GAPDH	Servicebio	GB11002
Anti-rabbit ACTB	Servicebio	GB11001
Alexa Fluor 488-conjugated donkey anti-mouse secondary antibodies	Abcam	Ab150113
Alexa Fluor 488-conjugated donkey anti-rabbit secondary antibodies	Abcam	Ab150077
Alexa Fluor 594-conjugated donkey anti-rabbit secondary antibodies	Abcam	Ab150116
Alexa Fluor 594-conjugated donkey anti-mouse secondary antibodies	Abcam	Ab150108
IRDye 800CW Goat Anti-Mouse IgG H&L	Bioss	bs-40296G-IRDye8
IRDye 800CW Goat Anti-Rabbit IgG H&L	Bioss	bs-40295G-IRDye8

Supplementary Table 2. Primers sequences for siRNA.

Gene Name	Primer Sequences
m-*Bcl2*	Sense: 5'-AUGAAUUACAAUUUUUCAGUCTT-3'<br>Anti-sense: 5'-CUGAAAAAUUGUAAUUCAUCUTT-3'
m-*Tsc-1*	Sense: 5'-AUACUCAUUAAUUUUGUCCAATT-3'<br>Anti-sense: 5'-GGACAAAAUUAAUGAGUAUGUTT-3'
m-*S1pr1*	Sense: 5'-ACUAUGAUAUCAUAGUUCCCATT-3'<br>Anti-sense: 5'-GGAACUAUGAUAUCAUAGUCC-3'
m-*Raptor*	Sense: 5'-GGAAGUCUUUGAACAGAAATT-3'<br>Anti-sense: 5'-UUUCUGUUCAAAGACUUCC-3'

Supplementary Table 3. Primers sequences for RT-qPCR.

Gene Name	Primer Sequences
m-*Gapdh*	Forward: 5'-AGCTTCGGCACATATTTCATCTG-3'<br>Reverse: 5'-CGTTCACTCCCATGACAAACA-3'
m-*Sphk1*	Forward: 5'-GGTGCTGGAACTGAACCTG-3'<br>Reverse: 5'-ACATGGGGCTGGAGAGAG-3'
m-*Sphk2*	Forward: 5'-GTTTGCCCTCACCCTCAC-3'<br>Reverse: 5'-AGCCCGAGACCTCATCC-3'
m-*S1pr1*	Forward: 5'-TCGTCCGGCTTGAGCGAG-3'<br>Reverse: 5'-GAGCTTTTCCTTGGCTGGAG-3'
m-*S1pr2*	Forward: 5'-ACAGCAAGTTCCACTCAGCAA-3'<br>Reverse: 5'-CTGCACGGGAGTTAAGGACAG-3'
m-*S1pr3*	Forward: 5'-CCATTGCCATTGAGCGACAC-3'<br>Reverse: 5'-TTAGCCAGCACATCCCAATCA-3'
m-*Sgpp1*	Forward: 5'-GATGCAGAGACCGAGGTTCG-3'<br>Reverse: 5'-CGGCAAGTTGCTCACTTTGAC-3'
m-*Sgpp2*	Forward: 5'-CACCCACTGGAATATCGACCC-3'<br>Reverse: 5'-AAGTCTCACAACGGGAGGAAA-3'
m-*Spl*	Forward: 5'-CTGAAGGACTTCGAGCCTTATTT-3'
	Reverse: 5'-GACACTCCACGCAATGAGC-3'
m-Cyclin d1	Forward: 5'-AGAACAAGCAGACCATCCGC-3'
	Reverse: 5'-GTCCTTGTTTAGCCAGAGGC-3
m-Raptor	Forward: 5'-ATGTGCACAGCCCATTCTT-3'
	Reverse: 5'- CGACAGGGCCAAGCTCA-3

Supplementary Table 4. Echocardiology analysis of wild-type (*WT*) and *S1pr1*^{CMKO} mice which underwent the sham operation or the AR operation at postnatal day 3 (P3), hearts were measured at 21-day post AR by echocardiography in the indicated

groups.

	Sham		Post-AR	
	WT (N = 9)	*S1pr1*^{CMKO} (N = 10)	*WT* (N = 9)	*S1pr1*^{CMKO} (N = 10)
HR (beats/min)	404.53 ± 13.03	410.48 ± 13.39	409.15 ± 15.74	422.79 ± 17.34
IVS; d (mm)	0.59 ± 0.05	0.59 ± 0.05	0.63 ± 0.03	0.67 ± 0.07
IVS; s (mm)	1.04 ± 0.05	0.98 ± 0.07	1.07 ± 0.03	0.93 ± 0.08
LVID; d (mm)	2.85 ± 0.12	2.78 ± 0.19	3.41 ± 0.07	3.30 ± 0.15
LVID; s (mm)	1.73 ± 0.16	1.80 ± 0.14	2.19 ± 0.11	2.52 ± 0.13
LVPW; d (mm)	0.61 ± 0.04	0.70 ± 0.04	0.75 ± 0.05	0.86 ± 0.06
LVPW; s (mm)	0.87 ± 0.05	1.03 ± 0.06	1.08 ± 0.06	0.93 ± 0.05
LVEF (%)	70.95 ± 4.35	66.21 ± 2.26	63.14 ± 3.00	46.76 ± 5.17*
LVFS (%)	39.97 ± 3.47	35.28 ± 1.74	33.69 ± 2.01	23.40 ± 2.99*
LV Mass (mg)	38.04 ± 4.71	41.35 ± 6.68	59.13 ± 1.87	65.52 ± 5.93

Data are means ± SEM. *P < 0.05 indicates significant statistical differences, compared with wild-type (*WT)* post AR groups by One-way ANOVA for multiple comparisons. HR, Heart Rate; IVS, Interventricular Septum; LVID, Left Ventricular Internal Dimension; LVPW, Left Ventricular Posterior Wall; LVEF, Left Ventricular Ejection Fraction; LVFS, Left Ventricular Fractional Shortening; LV Mass, Left Ventricular Mass.

Supplementary Table 5. Echocardiology analysis of wild-type (*WT*) and *S1pr1*^{CMKO} 8-week-old mice subjected to MI operation, and hearts were measured at 28-day post MI by echocardiography in the indicated groups.

	Sham		Post-MI	
	WT (N = 6)	*S1pr1*^{CMKO} (N = 6)	*WT* (N = 7)	*S1pr1*^{CMKO} (N = 7)
HR (beats/min)	579.18 ± 19.11	568.86 ± 16.64	417.7 ± 20.55	420.51 ± 36.98
IVS; d (mm)	0.80 ± 0.06	0.91 ± 0.05	0.81 ± 0.06	0.87 ± 0.05
IVS; s (mm)	1.41 ± 0.06	1.33 ± 0.09	1.16 ± 0.10	1.12 ± 0.11
LVID; d (mm)	3.91 ± 0.20	3.57 ± 0.20	4.59 ± 0.23	4.34 ± 0.20
LVID; s (mm)	2.69 ± 0.22	2.42 ± 0.22	3.60 ± 0.24	3.62 ± 0.15
LVPW; d (mm)	0.84 ± 0.08	0.87 ± 0.06	0.80 ± 0.07	0.72 ± 0.06
LVPW; s (mm)	1.38 ± 0.10	1.33 ± 0.07	0.97 ± 0.06	0.85 ± 0.11
LVEF (%)	61.43 ± 3.18	61.41 ± 4.26	43.93 ± 3.26	32.88 ± 3.00*
LVFS (%)	32.52 ± 2.30	32.61 ± 2.89	21.81 ± 1.81	14.96 ± 1.64*
LV Mass (mg)	93.03 ± 6.14	93.45 ± 11.84	123.33 ± 16.8	107.57 ± 9.94

Data are means ± SEM. *P < 0.05 indicates significant statistical differences,

compared with wild-type (*WT)* post-MI groups by One-way ANOVA for multiple comparisons. HR, Heart Rate; IVS, Interventricular Septum; LVID, Left Ventricular Internal Dimension; LVPW, Left Ventricular Posterior Wall; LVEF, Left Ventricular Ejection Fraction; LVFS, Left Ventricular Fractional Shortening; LV Mass, Left Ventricular Mass.

Supplementary Table 6. Echocardiology analysis of neonatal AAV9-*cTNT-S1pr1-GFP* and AAV9-*cTNT-GFP* mice which underwent the sham operation or the AR operation at postnatal day 3 (P3), and hearts were measured at 21-day post AR in the indicated groups.

	Sham		Post-AR	
	AAV9-*cTnT-GFP* (N = 4)	AAV9-*cTnT-S1pr1-GFP* (N = 4)	AAV9-*cTnT-GFP* (N = 5)	AAV9-*cTnT-S1pr1-GFP* (N = 6)
HR (beats/min)	404.07 ± 32.86	428.01 ± 19.20	408.27 ± 14.87	415.72 ± 15.9
IVS; d (mm)	0.62 ± 0.10	0.77 ± 0.11	0.70 ± 0.08	0.78 ± 1.10
IVS; s (mm)	0.91 ± 0.13	0.99 ± 0.19	1.10 ± 0.11	1.19 ± 0.08
LVID; d(mm)	3.73 ± 0.16	3.35 ± 0.18	3.25 ± 0.11	3.32 ± 0.16
LVID; s (mm)	2.62 ± 0.11	2.35 ± 0.31	2.21 ± 0.05	2.29 ± 0.17
LVPW; d (mm)	0.78 ± 0.09	0.87 ± 0.15	0.81 ± 0.06	0.92 ± 0.13
LVPW; s (mm)	1.00 ± 0.02	1.04 ± 0.24	1.15 ± 0.06	1.33 ± 0.09
LVEF (%)	56.59 ± 5.59	58.03 ± 8.31	57.08 ± 1.31	65.36 ± 2.46*
LVFS (%)	29.45 ± 3.79	30.63 ± 5.52	25.86 ± 2.82	34.41 ± 1.43*
LV Mass (mg)	70.14 ± 7.64	73.34 ± 12.19	65.34 ± 9.85	76.41 ± 6.91

Data are means ± SEM. *P < 0.05 indicates significant statistical differences, compared with AAV9-*cTNT-GFP* post AR groups by One-way ANOVA for multiple comparisons. HR, Heart Rate; IVS, Interventricular Septum; LVID, Left Ventricular Internal Dimension; LVPW, Left Ventricular Posterior Wall; LVEF, Left Ventricular Ejection Fraction; LVFS, Left Ventricular Fractional Shortening; LV Mass, Left Ventricular Mass.

Supplementary Table 7. Echocardiology analysis of neonatal AAV9-*cTnT-S1pr1-GFP* and AAV9-*cTnT-GFP* mice which underwent the AR operation at postnatal day 3 (P3), and hearts were measured at 21-day post AR with or without rapamycin treatment in the indicated groups.

	DMSO		Rapamycin
	AAV9-*cTnT-GFP* (N = 4)	AAV9-*cTnT-S1pr1-GFP*	AAV9-*cTnT-S1pr1-GFP*

		(N = 4)	(N = 6)
HR (beats/min)	406.84 ± 30.97	408.68 ± 24.70	414.39 ± 17.86
IVS; d (mm)	0.68 ± 0.11	0.79 ± 0.18	0.68 ± 0.08
IVS; s (mm)	1.03 ± 0.15	1.06 ± 0.17	1.02 ± 0.08
LVID; d (mm)	3.35 ± 0.20	3.36 ± 0.27	3.28 ± 0.11
LVID; s (mm)	2.28 ± 0.15	2.12 ± 0.26	2.37 ± 0.10
LVPW; d (mm)	0.76 ± 0.08	0.75 ± 0.11	0.71 ± 0.08
LVPW; s (mm)	1.12 ± 0.11	1.26 ± 0.06	0.95 ± 0.07
LVEF (%)	58.49 ± 1.74	68.19 ± 4.54*	55.09 ± 1.91#
LVFS (%)	27.64 ± 2.60	37.26 ± 3.48*	27.81 ± 1.22#
LV Mass (mg)	62.45 ± 12.12	68.74 ± 14.93	57.21 ± 7.60

Data are means ± SEM. *P < 0.05 indicates significant statistical differences, compared with AAV9-*cTNT-GFP* mice treated with DMSO groups by One-way ANOVA for multiple comparisons. #P < 0.05 indicates significant statistical differences, compared with AAV9-*cTnT-S1pr1-GFP* mice treated with DMSO groups by One-way ANOVA for multiple comparisons. HR, Heart Rate; IVS, Interventricular Septum; LVID, Left Ventricular Internal Dimension; LVPW, Left Ventricular Posterior Wall; LVEF, Left Ventricular Ejection Fraction; LVFS, Left Ventricular Fractional Shortening; LV Mass, Left Ventricular Mass.

Supplementary Table 8. Echocardiology analysis of wild-type (*WT)* and *S1pr1*^{CMKO} mice subjected to AR operation at postnatal day 3 with or without rapamycin treatment, hearts were measured at 21-day post AR by echocardiography in the indicated groups.

	DMSO		Rapamycin
	WT (N = 7)	*S1pr1*^{CMKO} (N = 8)	*S1pr1*^{CMKO} (N = 7)
HR (beats/min)	434.69 ± 23.34	458.54 ± 15.12	463.26 ± 22.25
IVS; d (mm)	0.64 ± 0.05	0.72 ± 0.04	0.67 ± 0.09
IVS; s (mm)	1.06 ± 0.03	1.03 ± 0.05	1.14 ± 0.12
LVID; d (mm)	3.17 ± 0.16	3.24 ± 0.14	3.10 ± 0.20
LVID; s (mm)	1.94 ± 0.14	2.22 ± 0.13	2.18 ± 0.27
LVPW; d (mm)	0.78 ± 0.09	0.83 ± 0.08	0.76 ± 0.10
LVPW; s (mm)	1.13 ± 0.08	1.08 ± 0.06	1.12 ± 0.12
LVEF (%)	60.14 ± 3.11	50.19 ± 2.89*	48.72 ± 3.31
LVFS (%)	38.94 ± 2.70	31.36 ± 1.95*	31.14 ± 5.91
LV Mass (mg)	55.99 ± 6.73	64.91 ± 6.08	54.65 ± 6.64

Data are means ± SEM. *P < 0.05 indicates significant statistical differences in *S1pr1*^{CMKO} mice compared with wild-type (*WT)* mice treated with DMSO by One-way ANOVA for multiple comparisons, while no statistical significance in these mice compared with *S1pr1*^{CMKO} mice treated with rapamycin by One-way ANOVA for multiple comparisons. HR, Heart Rate; IVS, Interventricular Septum; LVID, Left

Ventricular Internal Dimension; LVPW, Left Ventricular Posterior Wall; LVEF, Left Ventricular Ejection Fraction; LVFS, Left Ventricular Fractional Shortening; LV Mass, Left Ventricular Mass.

Supplementary Table 9. Echocardiology analysis of AAV9-*cTnT-S1pr1-GFP* and AAV9-*cTnT-GFP* adult mice with MI operation treated with or without rapamycin, hearts were measured at 28-day post MI by echocardiography in the indicated groups.

	Sham		Post-MI	
	AAV9-*cTnT-GFP* (N = 3)	AAV9-*cTnT-S1pr1-GFP* (N = 3)	AAV9-*cTnT-GFP* (N = 5)	AAV9-*cTnT-S1pr1-GFP* (N = 5)
HR (beats/min)	510.65 ± 25.47	463.59 ± 33.77	421.57 ± 18.7	419.42 ± 34.64
IVS; d (mm)	0.76 ± 0.02	1.01 ± 0.13	0.65 ± 0.07	0.77 ± 0.05
IVS; s (mm)	1.37 ± 0.09	1.56 ± 0.04	0.77 ± 0.07	1.14 ± 0.15
LVID; d (mm)	3.21 ± 0.23	3.26 ± 0.04	4.25 ± 0.35	3.94 ± 0.29
LVID; s (mm)	2.08 ± 0.18	1.94 ± 0.23	3.69 ± 0.37	2.82 ± 0.32
LVPW; d (mm)	1.03 ± 0.12	1.03 ± 0.13	0.84 ± 0.17	0.97 ± 0.11
LVPW; s (mm)	1.27 ± 0.04	1.55 ± 0.11	1.24 ± 0.11	1.46 ± 0.18
LVEF (%)	65.15 ± 6.61	71.69 ± 7.12	29.06 ± 4.87	55.52 ± 6.64*
LVFS (%)	35.14 ± 4.85	40.77 ± 6.32	13.59 ± 2.45	29.09 ± 4.21*
LV Mass (mg)	78.08 ± 8.92	97.59 ± 17.36	94.18 ± 13.11	106.36 ± 17.57

Data are means ± SEM. *P < 0.05 indicates significant statistical differences, compared with AAV9-*cTNT-GFP* post-MI groups by One-way ANOVA for multiple comparisons. HR, Heart Rate; IVS, Interventricular Septum; LVID, Left Ventricular Internal Dimension; LVPW, Left Ventricular Posterior Wall; LVEF, Left Ventricular Ejection Fraction; LVFS, Left Ventricular Fractional Shortening; LV Mass, Left Ventricular Mass.

Supplementary Table 10. Echocardiology analysis of AAV9-*cTnT-S1pr1-GFP* and AAV9-*cTnT-GFP* 8-week-old mice which underwent MI operation in the indicated groups treated with or without rapamycin, and hearts were measured at 28-day post MI by echocardiography in the indicated groups.

	DMSO		Rapamycin	
	AAV9-*cTnT-GFP* (N = 6)	AAV9-*cTnT-S1pr1-GFP* (N = 6)	AAV9-*cTnT-GFP* (N = 6)	AAV9-*cTnT-S1pr1-GFP* (N = 6)
HR (beats/min)	422.18 ± 63.82	470.55 ± 29.99	420.28 ± 53.52	490.91 ± 21.97

IVS; d (mm)	0.84 ± 0.11	0.79 ± 0.12	0.79 ± 0.31	0.82 ± 0.09
IVS; s (mm)	1.19 ± 0.15	1.31 ± 0.08	1.21 ± 0.11	1.34 ± 0.06
LVID; d (mm)	3.82 ± 0.23	3.98 ± 0.33	3.62 ± 0.33	4.61 ± 0.21
LVID; s (mm)	3.15 ± 0.24	2.88 ± 0.32	3.21 ± 0.11	3.58 ± 0.29
LVPW; d (mm)	1.03 ± 0.13	0.91 ± 0.10	1.10 ± 0.12	1.07 ± 0.12
LVPW; s (mm)	1.46 ± 0.14	1.37 ± 0.11	1.52 ± 0.11	1.29 ± 0.14
LVEF (%)	37.88 ± 3.33	54.97 ± 4.15*	32.93± 1.64	31.58 ± 6.46$^{\#}$
LVFS (%)	18.02 ± 1.78	28.39 ± 2.56*	19.21 ± 1.53	15.28 ± 3.42$^{\#}$
LV Mass (mg)	107.12 ± 11.57	99.81 ± 10.21	114.13 ± 12.54	116.28 ± 4.05

Data are means ± SEM. *P < 0.05 indicates significant statistical differences, compared with AAV9-*cTNT-GFP* mice treated with DMSO groups by One-way ANOVA for multiple comparisons. $^{\#}$P < 0.05 indicates significant statistical differences, compared with AAV9-*cTnT-S1pr1-GFP* mice treated with DMSO groups by One-way ANOVA for multiple comparisons. HR, Heart Rate; IVS, Interventricular Septum; LVID, Left Ventricular Internal Dimension; LVPW, Left Ventricular Posterior Wall; LVEF, Left Ventricular Ejection Fraction; LVFS, Left Ventricular Fractional Shortening; LV Mass, Left Ventricular Mass.
